# Relative contributions of neutral and non-neutral genetic differentiation to inform conservation of steelhead trout across highly variable landscapes

**DOI:** 10.1111/eva.12174

**Published:** 2014-06-27

**Authors:** Andrew P Matala, Michael W Ackerman, Matthew R Campbell, Shawn R Narum

**Affiliations:** 1 Columbia River Inter-Tribal Fish CommissionHagerman, ID, USA; 2 Eagle Fish Genetic Laboratory, Pacific States Marine Fisheries CommissionEagle, ID, USA; 3 Eagle Fish Genetic Laboratory, Idaho Department of Fish and GameEagle, ID, USA

**Keywords:** association tests, Columbia River Basin, conservation, selection and adaptation, steelhead trout

## Abstract

Mounting evidence of climatic effects on riverine environments and adaptive responses of fishes have elicited growing conservation concerns. Measures to rectify population declines include assessment of local extinction risk, population ecology, viability, and genetic differentiation. While conservation planning has been largely informed by neutral genetic structure, there has been a dearth of critical information regarding the role of non-neutral or functional genetic variation. We evaluated genetic variation among steelhead trout of the Columbia River Basin, which supports diverse populations distributed among dynamic landscapes. We categorized 188 SNP loci as either putatively neutral or candidates for divergent selection (non-neutral) using a multitest association approach. Neutral variation distinguished lineages and defined broad-scale population structure consistent with previous studies, but fine-scale resolution was also detected at levels not previously observed. Within distinct coastal and inland lineages, we identified nine and 22 candidate loci commonly associated with precipitation or temperature variables and putatively under divergent selection. Observed patterns of non-neutral variation suggest overall climate is likely to shape local adaptation (e.g., potential rapid evolution) of steelhead trout in the Columbia River region. Broad geographic patterns of neutral and non-neutral variation demonstrated here can be used to accommodate priorities for regional management and inform long-term conservation of this species.

## Introduction

Management strategies implemented for species conservation are highly contingent on a host of correlated life history and demographic information. In concert with these data, genetic structure is vital for characterizing population distinctions and limitations on productivity related to the decline of many species (e.g., conifer *Cathaya argyrophylla pineceae*, Ge et al. 1998; Chinese cobra *Naja atra*, Lin et al. 2012; gopher tortoise *Gopherus polyphemus*, Clostio et al. 2012; Chinook salmon, Moran et al. 2013). The genetic differentiation of populations across a species range is often determined on the basis of phylogenetic origins (Wagner et al. 2005), and historical and contemporary demography (Ruokonen et al. 2004). More recently, genetic variation has been viewed from the perspective of physical landscapes or environmental variation (Manel et al. 2003; Schoville et al. 2012). A landscape genetics approach reveals population variation relative to the influences or features in an organism's environment (Segelbacher et al. 2010; Sepulveda-Villet and Stepien 2012), including natural or human erected barriers and local climate. Most often it has been described on the basis of neutral divergence (Dionne et al. 2008; Narum et al. 2008), where restricted gene flow is explained in the context of a heterogeneous, patchwork environment (Latch et al. 2011).

Conservation units, such as a distinct population segment (DPS), are established based on a core set of criteria including population ecology and viability, ancestry and descent, reproductive isolation, and local adaptation (Fraser and Bernatchez 2001; Fraser et al. 2011). Local adaptation may be inferred from neutral genetic structure coincident with habitat or life-history variability (Olsen et al. 2011; Blankenship et al. 2011). However, direct evaluations of non-neutral population differentiation (i.e., putatively adaptive divergence) are likely to reveal stronger, more correlative relationships (Limborg et al. 2011). Nevertheless, conservation is not commonly informed by non-neutral variation, and inferences on adaptation based exclusively on neutral differentiation risk incorrectly identifying the underlying processes affecting population distinctions (Funk et al. 2012; Landguth and Balkenhol 2012).

Although full genome sequence data are typically not available for nonmodel species, evaluations of adaptive variation have recently been addressed using analyses of single nucleotide polymorphism (SNP) loci (Willing et al. 2010; Matala et al. 2011; Hohenlohe et al. 2010). Because SNP loci are commonly found within or adjacent to coding and regulatory regions of a genome, their allele frequencies may be influenced by selection (i.e., non-neutral). Techniques, such as association testing and detection of outlier loci, allow evaluation of differentiation that provides an improved understanding (over neutral loci) of the relationship between signatures of adaptive variation and the physical environment, even without direct interpretations of phenotypic variation, or interrogation of specific functional genes (Narum et al. 2010b; Matala et al. 2011; Ackerman et al. 2012; Bourret et al. 2013).

Understanding the distribution of adaptive variation across landscapes will be crucial in establishing conservation priorities (see Crandall et al. 2000; Fraser and Bernatchez 2001), and for anticipating how populations might be affected by local and regional changes in climate (Holderegger and Wagner 2008; Isaak et al. 2012a). The effects of global climate changes (e.g., rising temperatures) have increasingly altered habitats of myriad species, garnering the attention of a broad spectrum of researchers (Hickling et al. 2005; Milner et al. 2008; Winfield et al. 2010). Climate changes can prompt organisms to alter their behavior through range expansions (Loarie et al. 2009), or adapt over short time periods (rapid evolution; Hoffmann and Sgrò 2011; Barrett et al. 2010). Some of the most profound examples are found among organisms limited by confined habitats such as those of fishes, whose habitats are prescribed by water routes (e.g., networks of streams and lakes). Because of this limitation, they are particularly susceptible to environmental changes including water quality and temperature (Hari et al. 2006; Rieman et al. 2007; Wenger et al. 2011). Fish adapt variably to thermal conditions in their migratory environment (thermal optimum for aerobic scope), and even populations within the same subbasin may be affected disproportionately by dramatic thermal shifts (Farrell et al. 2008).

In the Columbia River Basin (CRB), steelhead trout (*Oncorhynchus mykiss*) occur as two evolutionarily divergent lineages, delineated east (inland) and west (coastal) of the Cascade Mountain Crest. The inland redband trout (*O. m. gairdneri*) are typically a stream maturing, summer-run type, while coastal rainbow trout (*O. m. irideus*) are dominated by an ocean maturing, winter-run type (Busby et al. 1996; Behnke 2002; Currens et al. 2009; Blankenship et al. 2011). Some populations of *O. m. irideus* also have a summer-run life history, though not necessarily genetically differentiated from sympatric winter-run populations (Busby et al. 1996). Owing to persistent steelhead trout population declines throughout the region, managers have implemented extensive monitoring and evaluation efforts (Busby et al. 1996; Chilcote 1998; ICTRT 2003; Scott 2008; Fryer et al. 2012). Five steelhead trout DPSs have been delineated within the CRB, and each is currently recognized for protection under the Endangered Species Act (ESA): the Upper Willamette River, Lower Columbia River, Middle Columbia River, Upper Columbia River, and the Snake River Basin (U.S. Office of the Federal Register 2006). Rivers in proximity to the Columbia River estuary lie within the Southwest Washington DPS. These conservation demarcations are largely contingent on adjacency of watersheds within stream networks, coupled with life history distinctions, and neutral genetic population structure. To date, there has been little to no direct interpretation of non-neutral variation for conservation assessment (Beacham et al. 1999; ICTRT 2003; Good et al. 2005; USOFR 2006; Nielsen et al. 2009).

Studies that describe distinctions among particular steelhead trout populations are plentiful (Chilcote et al. 1986; Zimmerman and Reeves 2000; Hendry et al. 2002; Matala et al. 2008), and address some local conservation concerns. However, more extensive evaluations are necessary to accommodate broad conservation management priorities in a regional context (Beacham et al. 1999; Winans et al. 2004; Currens et al. 2009; Blankenship et al. 2011). In this study, we investigate patterns of neutral genetic variation in contrast to non-neutral (putatively adaptive) genetic variation. We employed a multi-phased test approach to categorize non-neutrality (selection candidacy) of loci that was procedurally similar to several previous studies (Narum et al. 2010b; Hess and Narum 2011; Matala et al. 2011). Our primary objective was to provide an extensive characterization of genetic variation of steelhead trout throughout the entire Columbia River drainage to inform conservation management of this species. Study questions are threefold: (i) Is neutral population structure using SNPs consistent with previous studies that utilized other marker types [Winans et al. 2004; Currens et al. 2009; Blankenship et al. 2011]?, (ii) Is there significant evidence for candidate SNPs under selection and indications of putative adaptive variation?, and (iii) How do patterns of neutral and non-neutral genetic variation compare and contrast among populations that occupy highly variable environments across the landscape. Lastly, we discuss how non-neutral differentiation in relation to climate change may improve our understanding of population viability, and promote informed conservation that will complement existing methods implemented for the practical management of many diverse and often imperiled species.

## Methods

### Sampling, genotyping, and descriptive statistics

**Figure 1:**
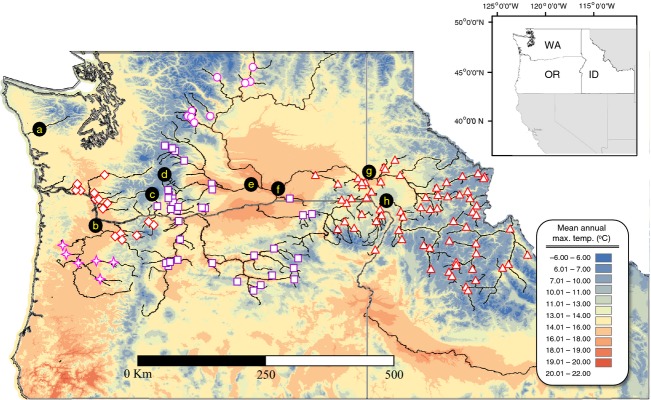
Map of the Columbia River Basin identifying locations of steelhead populations. Shading of the map reflects mean annual temperature maximums across the region from the period 1971–2000 (http://www.prism.oregonstate.edu/docs/meta/tmax_30s_meta.htm#7). Populations by DPS are: triangles – Snake River, circles – upper Columbia River, squares – middle Columbia River, stars – Upper Willamette River, and diamonds – lower Columbia River. Landmarks are: a – Quinault River, b – confluence of Willamette and Columbia rivers, c – Big White Salmon River, d – Klickitat River, e – Yakima River, f – confluence of Snake and Columbia rivers, g – confluence of Snake and Clearwater rivers, and h – confluence of Snake and Salmon rivers.

Our final data set consisted of 9011 steelhead trout samples, representing 145 collections spanning the sampling years 1996–2011 (Table 1; Table S1). Collections will hereafter be referred to as populations. The data set was primarily comprised of natural-origin populations (*n* = 133), but some hatchery exceptions are included (*n* = 12). Both the coastal and inland lineages were represented but in skewed numbers of populations: coastal (*n* = 24), and inland (*n* = 121; Table S1; Fig. 1). Sample sizes genotyped per population and by major population group (MPG) had minimum numbers ranging from *n* = 18 to *n* = 90, and maximums ranging from *n* = 30 to *n* = 164; Table 1). Genomic DNA was extracted from fin or opercle tissues of juvenile and adult fish preserved dry on Whatman paper (LaHood et al. 2008) or stored in individual vials containing 100% nondenatured ethanol. For DNA extraction, we used a standard Qiagen® DNeasy™ protocol, or Nexttec™ Genomic DNA Isolation Kits from XpressBio (Thurmont, MD, USA) following the manufacturer's standard protocol.

**Table 1:** Summary of *O. mykiss* populations, sample size, and characteristics by distinct population segment (DPS) and MPG in the Columbia River Basin. All listed DPSs have an ESA status listing of threatened with the exception of the Southwest Washington DPS, which also includes the Quinault River population from the coast of Washington State

						Genotyped (*n*)			
						
DPS	Tributary/Region	MPG	# pops	Lineage	Run	Min	Max	Mean	Total
Southwest WA	Columbia Estuary	n/a	4	Coastal	Winter	43	164	85.8	343
Lower Columbia	Clackamas	Cascade	4	Coastal	Mixed	43	92	60.3	241
Lower Columbia	Other	Cascade; Gorge	9	Coastal	Mixed	28	94	68.1	613
Upper Willamette	Willamette	Willamette	3	Coastal	Mixed	39	93	60.7	182
Upper Willamette	Willamette/West Side	Willamette	3	Coastal	Winter	25	30	27.0	81
Middle Columbia	Klickitat	Cascade East Slope	10	Inland	Summer	33	48	43.4	434
Middle Columbia	Deschutes	Cascade East Slope	5	Inland	Summer	31	63	51.4	257
Middle Columbia	John Day	John Day	10	Inland	Summer	18	107	36.3	363
Middle Columbia	Yakima	Yakima	7	Inland	Summer	21	59	36.9	258
Middle Columbia	Other	Mixed	7	Inland	Mixed	34	148	100.1	701
Upper Columbia	Wenatchee	Upper Columbia/East Slope Cascades	6	Inland	Summer	19	99	40.8	245
Upper Columbia	Other	Upper Columbia/East Slope Cascades	5	Inland	Summer	90	99	94.3	475
Snake	Lower Snake	Lower Snake	6	Inland	Summer	49	105	83.5	501
Snake	Lower Clearwater	Clearwater	5	Inland	Summer	49	156	107.8	539
Snake	M. F. Clearwater	Clearwater	14	Inland	Summer	35	99	56.4	789
Snake	S. F. Clearwater	Clearwater	4	Inland	Summer	36	104	57.8	231
Snake	Grande Ronde	Grande Ronde	7	Inland	Summer	45	95	62.7	439
Snake	Imnaha	Imnaha	4	Inland	Summer	23	61	41.5	166
Snake	S. F. Salmon	Salmon	4	Inland	Summer	39	45	43.5	174
Snake	M. F. Salmon	Salmon	8	Inland	Summer	23	84	48.3	386
Snake	Upper Salmon	Salmon	7	Inland	Summer	37	117	83.1	582
Snake	Other Salmon	Salmon	7	Inland	Summer	43	99	55.1	386
Snake	Hatchery	Mixed	6	Inland	Summer	89	146	104.2	625
Total			145						9011

A total of 191 SNP loci were genotyped with Taqman assays (Applied Biosystems, Grand Island, NY, USA). Our locus panels were comprised of SNPs developed by multiple sources (Table S2). All loci were ascertained from a broad coast-wide sample of populations, including Alaska, Washington, British Columbia, California, and Russia. Most SNPs were developed in a process of mining expressed sequence tags (ESTs) from GeneBank (https://www.ncbi.nlm.nih.gov/genbank/), followed by sequencing via primers developed for EST sequence flanking regions. Generally, the coding or noncoding nature of specific ESTs, and associated functions were unknown (see references for additional information, Tables S2 and S3). Forward and reverse primer/probe sequences for Taqman assays are available in Ackerman and Campbell (2012). Polymerase chain reaction (PCR) amplification of loci followed the protocol of Hess et al. (2012) and included an initial preamplification step. Successful genotyping for a given sample was defined proportionally as <10% missing data (i.e., fewer than 19 of 191 SNP genotypes per individual). Three species-specific SNP markers (*Ocl_gshpx-357*, *Omy_myclarp404-111*, and *Omy_Omyclmk438-96*) were used to screen for species ID and hybridization between *O. mykiss* and *O. clarkii* subspecies (Hess et al. 2012). All individuals identified as hybrids and congeners (*n* = 15 coastal, *n* = 79 inland) were subsequently removed from the data set prior to analyses. Following this screening exercise, the three species-diagnostic loci were removed from the data set.

Eight SNPs in our pared panel of 188 SNPs have been previously identified as *a priori* candidate loci for selection related to environmental variables. Specifically, two loci are putatively associated with thermal stress-induced mortality (Narum et al. 2013): *Omy_hsp47-86* and *Omy_OmyP9-180*. Five SNP loci (*Omy_aldB-165*, *Omy_gdh-271*, *Omy_Ogo4-212*, *Omy_stat3-273*, and *Omy_tlr5-205*) have been previously identified as associated with temperature variation in desert versus montane environments, and one locus (*Omy_hsf2-146*) was putatively associated with precipitation in the same study (Narum et al. 2010b). Two additional loci have been shown to differentiate anadromous and resident life-history types (Narum et al. 2011): *Omy_ndk-152* and *Omy_LDHB-2_i6*. In the following sections, these 10 loci are referred to as having ‘precedence’ in association tests (Table S3).

Locus-specific allele frequencies (i.e., minor allele frequency; MAF), observed heterozygosity (H_o_) and *F*_ST_ were generated with the program genalex version 6.2 (Peakall and Smouse 2006). Pairwise population *F*_ST_ was calculated in genepop v. 3.3 (Raymond and Rousset 1995), and significant among-group variation was determined at *P* < 0.01 using arlequin version 3.5 (Excoffier et al. 2005). Pairwise stream distances between population pairs were calculated using arcmap and a GIS application developed by D. Graves (CRITFC), available at: http://www.critfc.org/fish-and-watersheds/fishery-science/data-resources-for-scientists/critfc-data-download/. Mantel tests of isolation by distance (IBD) were evaluated in genalex v.6.2 using matrices of pairwise *F*_ST_ and pairwise stream distance. The Markov Chain Monte Carlo (MCMC) approximation of Fisher's exact test implemented in genepop v. 3.3 (1000 batches with 1000 iterations) was used to test for deviations from Hardy–Weinberg equilibrium (HWE) expectations, evaluated across 188 SNP loci and 145 populations. Linkage disequilibrium was tested for all pairs of loci among populations using a simulated exact test in genepop. For all pairs of loci with significant nonrandom association (linkage), the locus with the lower MAF was excluded from further analyses. Statistical significance (*α*) was adjusted for the number of simultaneous tests (initial *α* = 0.05) for both HWE and linkage tests via the B-Y FDR method (Benjamini and Yekutieli 2001) to reduce false-positive tests.

### Population structure within and among lineages

Throughout our methods and analytical approach, the coastal and inland lineages were evaluated separately. First, we verified the resolving power of our SNP markers to discretely differentiate the two steelhead lineages that have been characterized in previous studies based on other genetic markers (e.g., allozymes, Currens et al. 2009; microsatellites, Blankenship et al. 2011), while confirming lineage of origin for each population. This test was conducted irrespective of classification of loci as neutral or non-neutral. The program structure version 2.3.4 (Pritchard et al. 2000) was used to estimate the mean admixture proportions (*Q*), or fractional group fidelity among individuals in each of 145 populations, setting *k* = 2 (two inferred groups; coastal and inland). Default settings were used for the ancestry model (i.e., admixture) and frequency model (i.e., correlated allele frequencies), with a burn-in of 50 000 and 250 000 MCMC repetitions. Locus-specific and global pairwise *F*_ST_ (θ of Weir and Cockerham 1984) were calculated within each lineage using genepop. A multivariate principle coordinates analysis (PCoA) was performed in genalex version 6.2 based on matrices of pairwise *F*_ST_ to quantify amounts of variation.

### Identifying putatively neutral loci

In classifying the nature of SNP loci, we define three possible outcomes or distinctions. A locus may be (i) – unaffected by selection (neutral), (ii) – directly under selection (affecting one or more traits under selection), or (iii) – indirectly affected by the process of selection (selection at a linked locus). In all subsequent discussion, the term ‘non-neutral’ variation will be used to represent the latter two distinctions. We used an outlier approach based on simulation methods initially proposed by Beaumont and Nichols (1996) to identify outlier SNP loci putatively under selection. We conducted outlier tests as implemented in arlequin version 3.5 (Excoffier et al. 2005) to test for excessively higher or lower *F*_ST_ than would be expected under the assumptions of neutrality (Beaumont and Nichols 1996; Beaumont and Balding 2004). Tests were performed using 100 simulated demes and 50,000 coalescent simulations to generate a null distribution under neutral expectations around observed *F*_ST_ values (with confidence intervals). Due to the potential high error rate of the hierarchical island model (Narum and Hess 2011), a finite island model was assumed for outlier tests. Average locus heterozygosity versus *F*_ST_ was plotted to represent the 1% and 99% quantiles, and loci lying below or above these quantiles were outliers putatively under balancing or directional selection, respectively.

Outlier loci can be neutral or non-neutral and therefore relying solely on outlier tests to characterize the selection candidacy of loci risks a resulting high rate of false positives. For example, populations may be highly differentiated because of demography, or skewed patterns of isolation by distance (Akey 2009; Hermisson 2009; Narum and Hess 2011). In fractal landscapes, such as stream networks, patterns of genetic structure can be coincident with patterns or complexity of stream branching, likely resulting in elevated variance around neutral *F*_ST_ (i.e., false-positive outliers; Fourcade et al. 2013). For our exploration of non-neutral differentiation, outlier methods were used in combination with regression models with control variables to test environmental associations. This reduces the risk of false-positive conclusions (Matala et al. 2011), and aids in identifying the influences of environmental or habitat heterogeneity on the spatial distribution of genetic diversity (inferred selection gradients).

### Defining physical and control variables in an association test framework

Physical habitat variables used to gauge environmental heterogeneity were chosen to represent regional and local climate regimes. Latitude and longitude coordinates for each population were obtained from field data or were estimated using arcmap version 10 (copyright © 2010 ESRI) and were used to gather all physical variable measurements (Tables S4 and S5). Migration distance from the Columbia River estuary and pairwise stream distances (kilometers) were calculated using the GIS application previously described. Elevation was obtained using Google Earth (Image © 2012 TerraMetrics) and verified for accuracy in arcmap version 10. Monthly averages for temperature and total precipitation measurements (rain and melted snow) were generated using parameter-elevation regressions on independent slopes model (PRISM) of the Oregon Climate Service; http://www.prism.oregonstate.edu/. Monthly average maximum and minimum air temperatures were simulated at 800-m cell resolution from a model based on climate normals from a 30-year period (1971–2000) in PRISM. Water temperature readings were unavailable, and therefore, we used air temperature readings as a proxy for in-stream temperature. In the Pacific Northwest region, stream temperature trends closely with air temperature, particularly on temporal scales (Isaak et al. 2012b). However, changes in stream temperatures occur more slowly and are affected by groundwater and riparian buffering, glaciation, and complex topography. In total, five physical variables were used to characterize local and regional climate: precipitation, temperature, elevation, geographic coordinates, and migration distance. We expected autocorrelation between all five variables. The precipitation and temperature variables we evaluated on the basis of annual and seasonal measurements. Geographic coordinates and migration distance add directional elements related to regional weather patterns.

The highly dependent nature of *F*_ST_ on demographic history may confound the ability to accurately identify selection candidate loci (Beaumont 2005). When isolating forces appear correlated with variation across the landscape (Narum and Hess 2011), it can be difficult to confidently differentiate demographic influences from non-neutral (putatively adaptive) associations. Prior to conducting association tests, we assembled panels of putatively neutral SNP loci (defined by exclusion of outliers) for each lineage, and as a conservative measure, we established control variables for underlying neutral population structure. Using structure version 2.3.4 (Pritchard et al. 2000), we evaluated *k* ranging from 1–8 clusters for each lineage, and the most likely number of distinct populations (*k*) was selected based on five iterations for each potential *k* value. We used program default settings for the Monte Carlo Markov Chain procedure, with 30 000 burn-in followed by 150 000 MCMC repetitions. The (Δ*k)* statistic derived from the second order rate of change of the likelihood function (Evanno et al. 2005) provided an improved estimate of the mode of true (*k),* and the mean *Q* values for each population were established using the full search algorithm in CLUMPP (Jakobsson and Rosenberg 2007). Secondly, a pairwise population *F*_ST_ matrix was generated in genepop to conduct PCoA in genalex version 6.2. The resulting (*Q*) inferred admixture proportions from structure and the first three Eigen vectors (EV) from PCoA analysis were used to control for underlying neutral population differentiation in subsequent association tests.

### Identifying candidate loci: association with environment

We used two regression methods to identify potential candidate markers in association with the previously described environmental or climate-related variables. First, population MAF at each locus was plotted against each of five physical variables (Table S2) in univariate linear regression analysis. The *P*-values for the correlation coefficient were calculated in Microsoft Excel. Statistical significance (*α* = 0.05) was adjusted for the number of simultaneous tests as a conservative measure using a Bonferroni correction to exclude false-positive results (Rice 1989). Eighteen populations from the genetically distinct Clearwater River are located in a geographic region characterized by high precipitation. All 18 ranked in the top 20 for greatest amount of spring and summer precipitation among 121 inland populations (variable nonindependence). In initial univariate regression analyses, 86 loci (48%) showed significant correlation with either spring or summer precipitation. Therefore, as a precautionary measure to guard against what appeared to be a spurious geographical influence, regression coefficients were recalculated in the absence of the Clearwater River group. Then, of the original 86 loci only those that maintained a significant correlation with precipitation were considered ‘true’ correlations.

Second, we used DISTLM *forward* (McArdle and Anderson 2001) to perform multivariate multiple regression with permutation tests on locus-specific pairwise *F*_ST_ distance matrices versus pairwise matrices of values for physical variables (Carmichael et al. 2007; Olsen et al. 2010; Hess et al. 2011; Matala et al. 2011). Conditional tests that permute residuals under a reduced model (Anderson and ter Braak 2003) were performed in DISTLM *forward* using a stepwise forward selection procedure that fits individual variables or sets of variables sequentially in the linear model. Once the most informative variable or set of variables (explaining greatest amount of variation) is established, the remaining variables are fit to the model, while those selected in previous steps are held as constants. Specific associations were determined on the basis of highest rank (*P* < 5%) variable for a given locus, and the method accounts for correlations that are likely present between the variables used to characterize climates (Tables S4–S7). The temperature, precipitation, and neutral control (*Q* and EV) variables were evaluated in respective ‘sets’ as demonstrated by Anderson et al. (2004), where seasonal averages for the former two were defined as: winter (January–March), spring (April–June), summer (July–September), and fall (October–December).

In summary, the first two criteria used to flag loci for further consideration as candidates in our multitest process of categorization were as follows: (i) significant *F*_ST_ outliers at a 99% confidence threshold or (ii) loci with precedence of association [see Table S3]. All 180 loci were tested for correlation based on linear regression (criterion #3). Lastly, all loci flagged as candidates based on the first three criteria were scrutinized on the basis of multivariate multiple regression with permutation tests (criterion #4). Note that as the final discriminatory step, when multivariate regression tests ranked a neutral control variable (*Q* or EV) as highest in significance among all tested variables, the locus in question was precluded from categorization as non-neutral (selection candidacy) regardless of results based on the other three criteria (e.g., linear correlation with environment). This approach resulted in three subpanels of SNP loci for genetic analysis: candidate loci – those meeting stipulations outlined previously, neutral loci – those showing strongest correlation with neutral structure, and ‘ambiguous’ loci. The latter category of SNP was primarily comprised of F_*ST*_ outlier loci or loci having precedence of association which ultimately failed to reach non-neutral status based on regression analysis. Ambiguous loci (by definition) could not be confidently characterized as neutral or non-neutral given conflicting test results and were therefore excluded from subsequent evaluations of genetic differentiation. Candidate loci putatively under selection were subsequently used to evaluate non-neutral or adaptive variation. Putatively neutral loci were used to evaluate underlying neutral (demographic) population structure.

### Comparing neutral and putatively adaptive variation

Using the newly established neutral and candidate SNP panels, comparisons were drawn based on multiple analyses to show the corresponding amount of variation described by both neutral and non-neutral variation (Nosil et al. 2007). Nonparametric Mantel tests for isolation as a function of environment were conducted using 9999 permutations of pairwise matrices of *F*_ST_ (within lineages) against absolute pairwise difference in values for environmental variables. Nei's standard genetic distance (Nei 1972) was calculated for each lineage, and distance was displayed in the topology of an un-rooted neighbor-joining (NJ) tree using the analysis program phylip version 3.68 (Felsenstein 2008). The SEQBOOT option was implemented to generate 1000 simulated data sets, and a consensus topology with bootstrap support was generated using the CONSENSE option. The program treeview version 1.6.6 (Page 1996) was used to graphically display the trees. Different trees were generated using either neutral or candidate SNP panels for each lineage.

A ranking method was employed based on averages for five climate variables to compare climate similarities that occurred in the clustering of populations within tree topologies. Specifically, populations were ranked in descending order (warmest to coolest) using corresponding mean maximum temperatures for both spring and summer independently. Elevation was ranked in ascending order assuming lowest elevation equals warmest climate. Lastly, populations were ranked in ascending order for mean precipitation (least equal to driest/warmest climate) in both spring and summer independently. Following independent ranking of populations for the five variables, the average rank across all five was used to order populations from most hot and dry to most cold and wet, and to compare relative climates in the tree topologies.

## Results

### Descriptive statistics and population differentiation

Data quality analyses indicated only minor issues concerning locus scoring accuracy, nonrandom association of loci, and population admixture. We observed departures from expected genotypic proportions in 208 of 27 260 tests (188 loci × 145 populations) at an adjusted significance threshold of *P* = 0.0046. Generally, the HWE deviations were not specific to any population or locus, spanning 100 of 145 populations, and 109 of 188 loci. Exceptions occurred in both Abernathy Creek (Ref. #12) and Canyon Creek (Ref. #9), each with 10 population-specific departures. There were also 12 HWE departures at locus *OMS00087*, which was therefore removed from all subsequent analyses. Tests for linkage disequilibrium revealed five pairs and one trio of loci that remained significantly out of equilibrium in at least 10% of populations after adjustment for multiple tests (*P*-value < 0.0001). Linked SNP pairs were as follows: (*OMS00133* and *Omy_rapd-167*), (*Omy_CRBF1-1* and *Omy_crb-106*), (*Omy_Il-1b_.028* and *Omy_Il1b-198*), (*Omy_SECC22b-88* and *OMS00169*), (*Omy_ndk-152* and *Omy_u09-52.284*), and the trio (*Omy_GHSR-121*, *OMS00176* and *Omy_mapK3-103*). In each pair, only the locus with the highest MAF was retained in the data set (Table S2).

Following paring of eight loci for HWE and linkage disequilibrium, the final data set included 180 loci for use in subsequent analyses. No SNP locus exhibited fixed allele frequencies, and variability ranged widely both within and between steelhead trout lineages. Among 24 coastal lineage populations, the mean observed heterozygosity ranged from H_o_ = 0.002 at locus *Omy_pad-196* to H_o_ = 0.531 at locus *OMS00101* (overall mean H_o_ = 0.315). Among 121 inland lineage populations, the range was H_o_ = 0.027 at locus *Omy_nach-200* to H_o_ = 0.513 at locus *Omy_IL17-185* (overall mean H_o_ = 0.302; Table S2). Among coastal lineage populations, we observed a mean locus MAF of 0.246 ranging from 0.001 at *Omy_impa1-55* to 0.498 at *Omy_arp-630*. The mean MAF across inland lineage populations was 0.236, ranging from 0.024 at *Omy_nach-200* to 0.497 at *OMS00070*. Generally, large MAFs among populations within lineages are indicative of highly differentiated populations. The overall mean pairwise F_*ST*_ between all coastal populations was 0.044. Population-specific mean values among 24 populations ranged from pairwise *F*_ST_ = 0.029 (ref.#'s 17) to pairwise *F*_ST_ = 0.079 (ref.#'s 11). With the exception of one pair of populations (ref.#'s 3 & 4; Eagle Creek, North Fork Eagle Creek), all comparisons indicated significant among-group variation (*P* < 0.001). The overall mean pairwise *F*_ST_ between all inland populations was 0.045. Population-specific mean values among 121 populations ranged from pairwise *F*_ST_ = 0.025 (ref.#'s 33) to pairwise *F*_ST_ = 0.148 (ref.#'s 77), and all pairwise population comparisons indicated significant among-group variation (*P* < 0.001). Significant isolation by distance (IBD) was observed in both lineages: coastal (*R*^2^ = 0.245, *P* < 0.0001) and inland (*R*^2^ = 0.083, *P* < 0.0001).

### Differentiating populations by lineage

**Figure 2:**
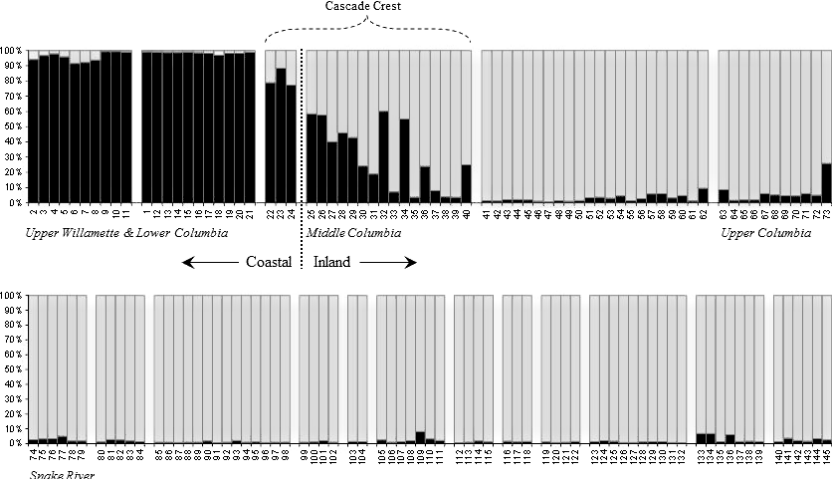
Mean admixture proportion plot of structure version 2.3.4 (Pritchard et al. 2000) generated from 180 SNP loci. The histogram shows admixture proportions for *k* = 2 inferred groups represented by black bars (coastal proportion) and gray bars (inland proportion). Populations correspond to reference numbers (Table S1) in ascending order from left to right (except Ref.#1 Quinault in the 12th position), and are arranged by DPS. Populations at the western extreme of the inland range, and eastern extreme of the coastal range are identified in relation to (adjacent) the Cascade Mountain crest.

When all coastal lineage populations were combined and all inland lineage populations were combined to form two groups (corresponding to lineage), we observed distinct allele frequency differences over the panel of 180 loci. Allele frequencies ranged from a low of 0.002 at *Omy_104519-624* to a high of 0.719 at *Omy_ndk-152*. At 41 different (bi-allelic) loci the minor allele in one lineage was the opposite (major allele) for the other lineage. The overall mean pairwise F_*ST*_ for comparisons between coastal and inland populations was 0.145. Population-specific means for interlineage comparisons ranged from pairwise *F*_ST_ = 0.053 (ref.#'s 25: Bowman Creek) in the inland lineage, to pairwise *F*_ST_ = 0.228 (ref.#'s 33; Upper Trout Creek) also an inland lineage population (data not shown). With the exception of 15 populations along the crest of the Cascade Mountains, the 145 populations in our analyses formed defined clusters according to lineage, where the first PCoA plot axis separated lineages and explained 63.4% of the total variation in the data. In Bayesian clustering analyses, the mean admixture proportion (*Q*) for two inferred populations was 95.3% in *Q*1 for coastal populations, and conversely, 93.8% for *Q*2 among inland populations (Fig. 2). Mean values would likely have been higher, but populations adjacent to the crest of the Cascade Mountains (demarcating range limits of coastal and inland types) appeared admixed between lineages to varying degrees, substantially lowering inferred group fidelity. The genetic characterization of the Big White Salmon River population (Fig. 1; c) was significantly more similar to the coastal lineage despite its location among the middle Columbia River DPS and was therefore evaluated throughout these analyses as a coastal population.

### Controlling for underlying neutral differentiation in association testing

Outlier tests revealed eight loci putatively under directional selection in the coastal lineage and 10 in the inland lineage (Tables S3, S6, and S7). No influence of balancing selection was observed. The remaining putative neutral loci in the inland lineage (*n* = 170) and coastal lineage (*n* = 172) were used in PCoA and Bayesian cluster analyses to establish neutral control variables. For both lineages, the most likely number of inferred populations was *k* = 2. However, this was likely a result of the relatively deep divergence between the Willamette River DPS and remaining lower Columbia River populations in the coastal lineage (ICTRT 2003), and similar divergence between the genetically distinct Klickitat River subbasin and remaining inland populations. To evaluate structure at a finer scale, we chose the appropriate number of inferred groups from the next peak in the second order rate of change (Δ*k*) for log_*e*_[Pr{*K*}], which occurred at *k* = 6 (*Q*1–*Q*6) within each lineage independently (Table S4).

For the coastal lineage, mean *Q* partially distinguished summer-run populations, a less common life-history type among this lineage (*Q*5 = 65%), two groups in the upper Willamette DPS (*Q*2 = 58% and *Q*4 = 61%), the Washington coast population from the Quinault River (*Q*1 = 89%), and the Big White Salmon population on the Cascade Crest (*Q6* = 81%). Most of the remaining lower Columbia populations had greatest proportions in *Q*3, ranging from 34% to 77%. For the inland lineage, the six inferred populations partially distinguished major subbasins, where the middle and south forks of the Salmon River were both dominant in *Q*1 (74%), the south and middle forks of the Clearwater River in *Q*4 (53%) and *Q*3 (76%) respectively, the Klickitat River in *Q*2 (68%), and the upper Salmon River in *Q*5 (56%). Regions with highest mean admixture proportion in *Q*6 included the majority from the upper and middle Columbia, and the Grande Ronde and Imnaha rivers in the Snake River Basin. Most individual populations within these regions did not exhibit definitive or strong fidelity in *Q*6 (ranging 27–68%, with a mean of 44%), and the highest admixture proportion by watershed occurred in the Yakima and John Day Rivers, with mean *Q*6 of 59% and 54%, respectively (Table S4). Differentiation was slightly higher within the inland lineage, with a larger range of pairwise population F_*ST*_ (0.0002–0.1889, mean 0.0421) compared to values within the coastal lineage (range 0.0001–0.1098, mean 0.0413). Results of PCoA based on pairwise *F*_ST_ corroborate primary distinctions revealed from structure analyses. Among the inland lineage, observed distinctions coincide well with several of the MPGs within the Snake River DPS.

### Association tests to identify non-neutral loci: putatively adaptive variation

The adjusted probability threshold for determining significant association using linear regression was *P* < 0.00029. On the basis of linear regression alone, we identified 12 loci in the coastal lineage that were significantly correlated with one or more environmental variables (Table 2; Table S3). In the inland lineage, 59 loci were identified as significantly correlated, including 17 loci for spring precipitation, and 21 loci for summer precipitation. From combined results using outlier tests, association precedence, and linear regression, we ultimately flagged 26 loci in the coastal lineage and 62 loci in the inland lineage for further examination of environmental correlation. Following the final ranking phase based on multivariate regression, the pared locus classifications included nine candidate loci putatively under selection in the coastal lineage and 22 candidates in the inland lineage (Table 2). All remaining loci were considered either neutral or ambiguous (Table S3). The group of loci ultimately classified as ambiguous were typically comprised of outlier loci for which the highest ranked variable was one of our control variables for neutral differentiation (Q or EV; Table S4). The SNPs deemed ambiguous may indeed be under selection, but we failed to identify associations with the suite of environmental variables tested here.

**Table 2:** Candidate SNP markers associated with climate for *O. mykiss* in the Columbia River Basin

SNP locus	Notes	Inland lineage	Climate associations			Coastal lineage	Climate associations		
Omy_hsc715-80		Candidate	P			Neutral			
Omy_SECC22b-88		Candidate	P			Neutral			
OMS00014		Candidate	P			Neutral			
OMS00062		Candidate	P			Neutral			
OMS00151		Candidate	T	P		Neutral			
Omy_97660-230		Candidate	T	D		Neutral			
Omy_CRBF1-1		Candidate	P	D		Neutral			
Omy_e1-147		Candidate	D	T		Neutral			
Omy_GHSR-121		Candidate	P			Neutral			
Omy_IL6-320		Candidate	T	P		Neutral			
Omy_metA-161		Candidate	T	P		Neutral			
Omy_nkef-241		Candidate	D			Neutral			
Omy_ntl-27		Candidate	D	P		Neutral			
Omy_u09-53.469	O_i_	Candidate	T	P	D	Neutral			
Omy_UT16_2-173	O_i_	Candidate	D			Neutral			
OMY1011SNP		Candidate	P			Neutral			
Omy_hsp47-86	(*)	Candidate	T	D		Ambiguous			
Omy_tlr5-205	(†)	Candidate	T	P	D	Ambiguous			
Omy_gdh-271	(†)	Candidate	E			Ambiguous			
Omy_97954-618	O_c_	Candidate	P	D		Ambiguous			
Omy_OmyP9-180	(*)	Candidate	T	P		Candidate	T		
Omy_stat3-273	(†)	Candidate	P			Candidate	P		
Omy_aldB-165	(†)	Ambiguous				Candidate	T		
Omy_hsf2-146	(†)	Ambiguous				Candidate	P	D	
OMS00008		Neutral				Candidate	P		
OMS00058		Neutral				Candidate	T	D	
OMS00111		Neutral				Candidate	P	D	
Omy_bcAKala-380rd	O_c_	Neutral				Candidate	T	D	
Omy_cox1-221		Neutral				Candidate	D	T	P

Notes include: inland ‘O_i,_’ and coastal outlier loci ‘O_c,_’ and reference numbered ‘precedence’ loci: ‘*’. Thermal stress association (Narum et al. 2013); ‘†’. Temperature or precipitation association (Narum et al. 2010b).

Climate associations are: P, precipitation; T, temperature; E, elevation; D, distance.

**Figure 3:**
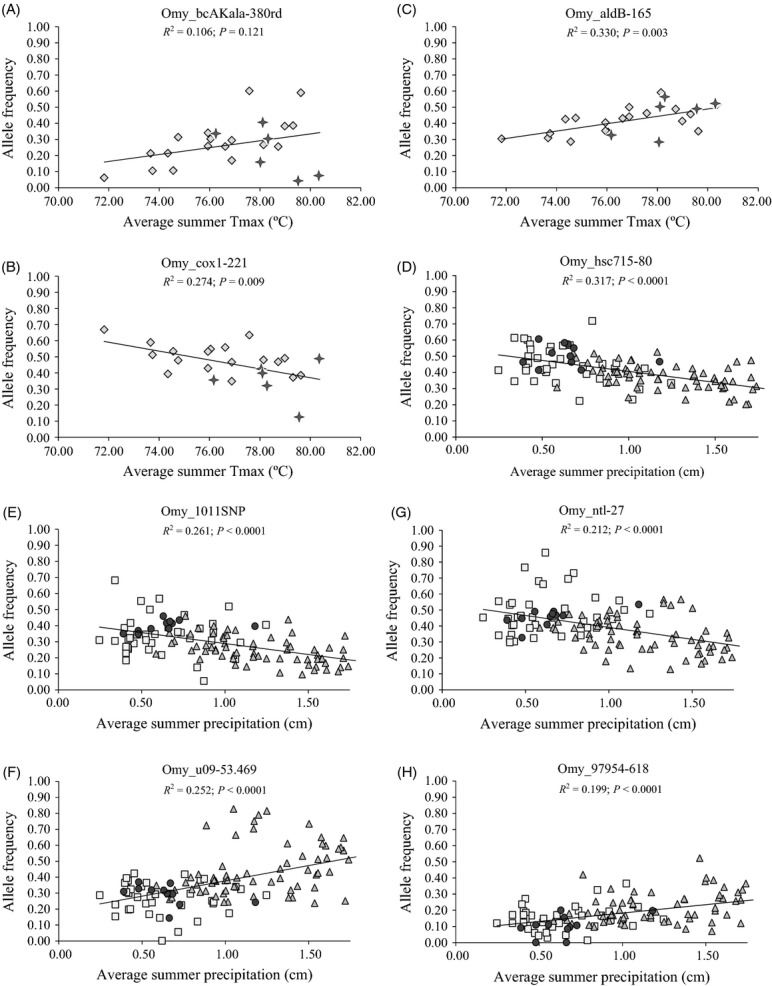
Linear regression plots, representing results for eight of the most highly correlated SNP loci (see Table 2). Plots A–C are temperature associations in the coastal lineage, while D–H are precipitation associations in the inland lineage. Symbols correspond with DPS: diamond – lower Columbia and Southwest Washington, star – upper Willamette, square – middle Columbia, circle – upper Columbia, and triangle – Snake.

Of the eight SNP loci showing precedence as candidates for local climate from previous studies, seven exhibited significant association with climate variables in at least one lineage in our broad-scale evaluation across many populations. Two loci previously identified as associated with survival under thermal stress (*Omy_hsp47-86* and *Omy-P9-180*; Narum et al. 2013), were significantly associated with either temperature or precipitation (Fig. 3). Two loci previously identified as candidates for temperature (*Omy_stat3-273 and Omy-gdh-271;* Narum et al. 2010b) were found to be highly correlated with precipitation in at least one lineage (Tables 2 and S7), but not for temperature. Previous associations of locus *Omy_hsf2-146* with precipitation, and locus *Omy_aldB-165* with temperature (Narum et al. 2010b) were corroborated within the coastal lineage. Finally, locus *Omy_tlr5-205* previously associated with temperature (Narum et al. 2010b) was significant for temperature and precipitation in the inland lineage. Notably, two of these loci (*Omy-P9-180*, *Omy_stat3-273*) were candidates for climate association in both lineages. Additional novel candidate loci were detected that have not previously been identified as loci putatively under selection.

The two loci that were previously determined to be associated with differentiating resident from anadromous life-history forms (*Omy_ndk-152* and *Omy_LDHB-2_i6*; Narum et al. 2011) could not be directly tested for association with life-history forms because most populations in our analyses were field identified as anadromous (e.g., juvenile smolt or adult steelhead phenotypes). However, *Omy_ndk-152* was a significant *F*_ST_ outlier. A single locus (*Omy_Ogo4-212*) with precedence of putative association for climate or life history had no confirmed associations for any habitat variable in our analyses and was deemed ambiguous as it may be a candidate at smaller, more local scales.

Among coastal lineage populations, precipitation and distance from the ocean (i.e., migration distance, and lat/long coordinates) were equally the most commonly correlated environmental factors. To clarify, the common point of origin for all measured migration distances was the Columbia River estuary, and therefore, distance associations were not necessarily an example of IBD gene flow which relates to direct distance between populations. In the inland lineage, environmental correlations were dominated by precipitation, then temperature. Specifically, spring, summer and total annual precipitation were the physical variables most often associated with genetic variation, and several significantly correlated loci spanned both lineages (Fig. 3). In some cases, the ranking of variables in multivariate regression was inconsistent between tests of individual variables versus sets of variables. For example, while an individual variable (e.g., *Q*1, summer precipitation) may have ranked highest for a given locus, the corresponding variable sets (e.g., *Q*1–*Q*6, total precipitation) may have ranked low for that same locus (Table S6 and S7). An explanation for this outcome, centered on differential environmental influences among life-history stages, follows in the discussion.

### Comparing neutral and non-neutral differentiation

**Figure 4:**
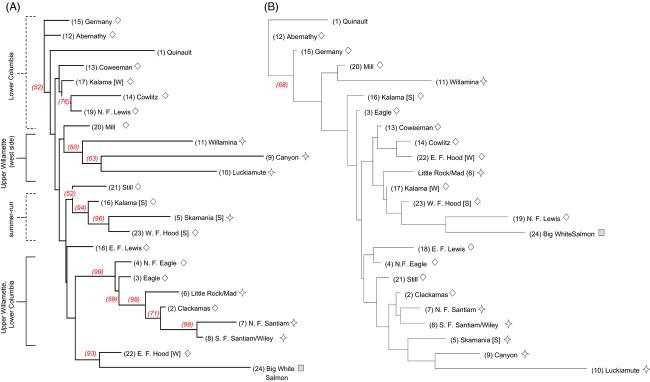
Neighbor-joining trees depicting Nei's genetic distances among coastal lineage populations based on (A) neutral variation – 158 SNPs, and (B) non-neutral variation – nine SNPs. Bootstrap support exceeding 50% appears at nodes. Diamonds represent the Lower Columbia DPS, while stars represent the Upper Willamette DPS. Numbers in parentheses correspond to population reference numbers (Table S1).

**Figure 5:**
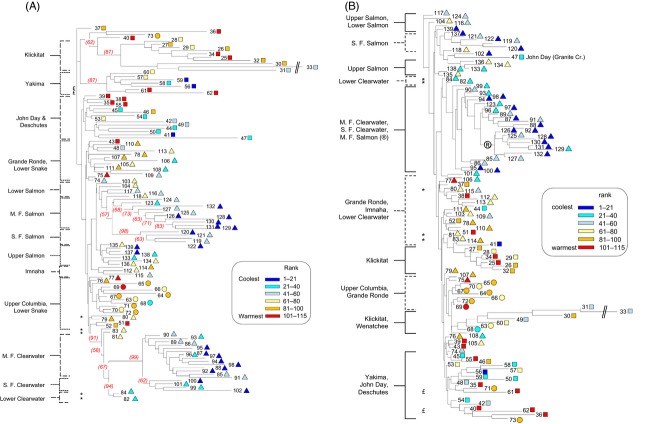
Neighbor-joining trees depicting Nei's genetic distances among inland lineage populations. Numbers at branch ends correspond to population reference numbers (Table S1) Trees are based on (A) neutral variation – 146 SNPs, and (B) non-neutral variation – 22 SNPs. Bootstrap support exceeding 50% appears at nodes. Population symbols correspond with DPS: triangles – Snake River, circles – upper Columbia River, squares – middle Columbia River. Major drainage sub-basins are labeled on the left. The symbol (*) represents climate related distinction among Lower Clearwater River populations and (£) represents differences among Yakima River populations based on climate. The symbol (®) identifies the branch adjoining Clearwater River and M. F. Salmon River populations. The color scale indicates population ranking by climate (1 = coldest, 115 = warmest) for 115 inland populations identified by reference number (Table S1).

The NJ tree topologies based on neutral SNP panels generally showed genetic distance relationships among populations that accurately aligned with the five DPS delineated under the ESA (Figs 4A and 5A). However, in the coastal lineage, the Clackamas River populations (Lower Columbia River DPS) grouped closely with populations in the upper Willamette River DPS, while populations in the upper Willamette River west side tributaries were distinctly partitioned from upper Willamette east slope populations (Fig. 4A). The known summer-run populations in the coastal lineage cluster together with significant bootstrap support, rather than clustering with winter-run populations from the same tributaries (i.e., Kalama and Hood rivers). Middle and Upper Columbia MPGs and five MPGs in the Snake River (Ford 2011) are well differentiated within the inland lineage, although bootstrap support was minimal in some instances. Finer scale definition observed in the Salmon and Clearwater rivers indicates significant genetic distinctions between middle and south fork populations from both subbasins (within MPGs), and between each of those groups and corresponding populations in the lower sections of both subbasins (Fig. 5A). These within-watershed distinctions in both the Clearwater River and Salmon River subbasins are in agreement with previous reports (Nielsen et al. 2009), but the level of resolution that differentiates the Clearwater River subbasin from the Salmon River subbasin has not been previously reported. In the tree topology for neutral structure in the inland lineage, populations within MPGs or subbasins were also frequently characterized by climate similarity. However, deviations from ESA- or regionally based (e.g., distance) clustering were rare regardless of differences or similarities in climate.

The NJ tree topologies based on non-neutral structure were comprised of all candidate SNPs in the coastal (*n* = 9 loci) and inland (*n* = 22 loci) lineages. Trees presented population clustering patterns that often did not correspond with delineations of DPS or MPG (Figs 4B and 5B), albeit with limited bootstrap support. This was presumably a function of topologies based on small numbers of loci. Climate based clustering patterns were more apparent in the inland lineage, where several of the relationships depicted in the non-neutral tree topology conformed to warm or cool climate similarity irrespective of subbasin or MPG distinction. For example, the neutrally distinct Klickitat River and Yakima River groups were each split to show some association to climate, most Clearwater and Salmon river groups with similar climates were combined on the same basal node or primary branch of the tree, and the five lower Clearwater River populations were differentiated by climate distinctions. Overall, NJ results based on non-neutral SNPs for the coastal lineage make it difficult to discern any basis for population similarities within branching patterns. In particular, climate rankings for coastal populations were noninformative for understanding genetic distance relationships associated with physical variables (Fig. 4A; climate ranks not shown).

Allele frequencies were most commonly correlated with the precipitation variables in association tests. This was further verified with Mantel tests of isolation by precipitation (IBP). Tests were based on a five-locus subset of nine candidate loci in the coastal lineage and a 15-locus subset of 22 candidate loci in the inland lineage; subsets were chosen based on significant associated with precipitation specifically (Table 2; Table S6 and S7). For the coastal lineage, a test based on all five loci indicated significant IBP for mean annual precipitation (*R*^2^ = 0.235; *P* = 0.0009), mean spring precipitation (*R*^2^ = 0.201; *P* = 0.0006), and mean summer precipitation (*R*^2^ = 0.296; *P* = 0.0004). The panel of 158 neutral loci for the coastal lineage was also tested to account for potentially confounding neutral patterns of IBP, but none were observed. Mantel tests of IBP based on 15 precipitation-associated loci in the inland lineage (pairwise comparisons between 121 populations) indicated significant IBP for mean spring precipitation (*R*^2^ = 0.039; *P* = 0.0001), and for mean summer precipitation (*R*^2^ = 0.037; *P* = 0.0001); no correlations were observed between seasonal precipitation and the panel of inland lineage neutral SNPs (*n* = 146).

## Discussion

This study demonstrates patterns of neutral and non-neutral variation in *O. mykiss* at a broad geographic scale that will be a valuable contribution to improved conservation management of this species in the CRB. Neutral structure was complementary to preceding studies (Winans et al. 2004; Currens et al. 2009; Blankenship et al. 2011) and confirms the existence of two deeply divergent steelhead trout lineages (i.e., coastal and inland) across the species range, along with finer scale population structure. We identified neutral divergence among steelhead trout populations that coincides substantially with current DPS delineations among lineages, as well as MPGs (ICTRT 2003; Good et al. 2005; Ford 2011) demarcated within the Snake River Basin (five MPGs), Middle Columbia River (four MPGs) and Upper Columbia River (one MPG). However, using our SNP panel, fine-scale resolution of neutral differentiation was detected in the Snake River DPS at a level previously unreported based on other marker types. Similar to other studies (Nielsen et al. 2009; Campbell et al. 2012), we found no evidence for multiple evolutionary lineages in the Snake River, but local environments may influence their differentiation. For reference, six Snake River hatchery stocks were genetically similar to their natural-origin counterparts that distinguish the Snake River DPS. In the coastal lineage, neutral differentiation among populations from east and west side tributaries in the upper Willamette River does not appear to fit DPS unit distinctions and may warrant further investigation to define conservation boundaries. From our analyses, the Big White Salmon river population is currently the only population within the Middle Columbia River DPS that is more consistent with a coastal lineage origin, suggesting further evaluation of its classification may be necessary.

Additionally, we show clear evidence for non-neutral (putatively adaptive) variation that is significantly associated with climate in the region. Specifically, several candidate markers were primarily associated with precipitation and temperature. This study demonstrates that candidate markers can be applied at broad geographic scales to describe the extent of potential local adaptations across highly variable climates. To evaluate non-neutral differentiation, our designation of candidate SNP loci was applied conservatively to reduce false-positive results. Conclusions of climate association were based on a large and diverse number of populations and supported by a framework of multiple test criteria and strict likelihood thresholds (Balkenhol et al. 2009; Schoville et al. 2012). De Mita et al. (2013) suggest using multiple robust methods, and emphasize numbers of populations over numbers of individuals per population for improved confidence in determining selection candidacy of loci. A greater number of candidate loci were discovered in the inland lineage, represented by a larger number of populations than were observed in the coastal lineage. In addition, the interior region of the CRB is larger, characterized by a highly variable environment relative to the coastal environment (greater range of wet/dry and warm/cool conditions). Several loci were identified in association with tested environment or landscape variables, but precipitation and temperature proved to be the most common (# loci) and strongest factors in non-neutral population differentiation that spanned both lineages (e.g., at SNP *Omy_stat3-273*, *Omy_OmyP9-180*).

Although our association tests indicated relationships between environmental variation and genetic heterogeneity (i.e., allele frequency variation), it is challenging to decipher the biological relevance of those correlations. Climate variables such as temperature may affect behaviors and phenotypes alike (Perry et al. 2001; Zydlewski et al. 2005), sometimes relatively rapidly in response to perturbations (Kovach et al. 2012). Previous landscape genetics studies in salmonids have shown significant allele frequency correlation with precipitation that have been described as neutral influences on population structure (Narum et al. 2008; Blankenship et al. 2011; Olsen et al. 2011). Olsen et al. (2011) for example, present a compelling discussion on possible correlations between precipitation and gene flow. This may occur if increased flooding results in decreased stream stability, which in turn may affect fish dispersal. Alternatively, the similarity of outcomes across study locales may suggest that the distribution of precipitation across the landscape elicits, or is indicative of common adaptive responses. For example, streambed scour related to rain-on-snow events may have lesser impact on fish that adapt by burying eggs at deeper depth (Goode et al. 2013). Thus, climatic variables such as precipitation may impart either ‘neutral’ landscape effects, ‘non-neutral’ (putatively adaptive) landscape effects, or both. In either case, one could argue that adaptation and selection play a key role in shaping the genetic landscape, which is more apt to be revealed through non-neutral genetic variation (Limborg et al. 2011).

The genetic population structure we observed differed markedly depending on whether our evaluations were based on neutral or non-neutral differentiation. Neutral differentiation generally reflected a pattern of distance-restricted gene flow, and in the context of demographic factors, population clustering was relatively intuitive within and among regions (clustering by subbasins, DPS, etc.). In contrast, population-clustering patterns identified using candidate loci (non-neutral differentiation) were presumably based upon environmental variation and frequently did not align with current steelhead trout DPS delineations. Moreover, clustering similarities based on non-neutral variation did not necessarily coincide with geographic proximity, nor were patterns among populations always transparent in regard to biology (e.g., coastal lineage migration timing). Thus, the juxtaposition of genetic signals show that neutrally dissimilar populations may exhibit non-neutral similarity (and vice versa) related to environment, and irrespective of geographic distance. Compared with neutral variation, candidate loci revealed some novel distinctions between populations, presumably reflective of environmental differences between regions and locales, particularly for the inland lineage. For the coastal lineage, less variable environments may produce moderate selective pressure for local adaptation among surveyed portions of the species range. More extreme environments in the southern portion of the coastal lineage range (e.g., the Sacramento River system) might be expected to provide stronger selective pressure.

The relative influences of climate or environment on genetic variation (e.g., putative adaptive responses) may occur during particular life-history stages of an organism, which can be difficult to discern. Temperature for example has been shown to have variable effect on different life stages of fish (Fowler et al. 2009). In our multivariate regression analysis, we noted inconsistencies between tests on individual physical variables and corresponding sets of seasonal variables. Seasonal environmental variation may impart a disproportionate selective influence coincident with age related behaviors (e.g., emergence time or outmigration time; Coleman and Fausch 2007). Our results of non-neutral differentiation indicate that precipitation during specific juvenile rearing or adult spawning periods may be more effectual or correlative than average annual fluctuations in precipitation.

If correlations between loci and physical variables are indicative of an adaptive influence (Bonin et al. 2009), they are not necessarily representative of direct causal relationships (e.g., selection for specific phenotypes). Note that from among our panel, the locus *Omy_stat3-273* was previously identified among desert and montane resident *O. mykiss* populations as being associated with temperature (Narum et al. 2010c). We demonstrated in our evaluation that this locus was correlated with precipitation but not with temperature, yet in actuality it is likely associated with overall climate and thus affected by myriad aspects of habitat variability. We identified patterns of putatively adaptive variation associated with climate, but corresponding phenotypic trait variation was not measured. However, distinguishing whether phenotypic changes are genetically based or the result of phenotypic plasticity has proven difficult (Merila and Hendry 2014). Often many genes are involved in adaptive responses to specific environments and/or climates (Kassahn et al. 2007) and controlled experiments would be necessary to make direct inferences on interactions between environments, phenotypes, and specific genes. Rather than providing unequivocal evidence of adaptation on the basis of phenotypic attributes, our study identifies locus associations that can be seen as indicators of related but undetermined causative environmental forces, such as early seasonal onset (Bradshaw and Holzapfel 2008). For example, distinct populations of steelhead trout in Oregon's Hood River occupy either glacial fed or spring fed tributaries (Underwood et al. 2003; Matala et al. 2009). Distinctions likely arose due in part to selection in variable environments, characterized by in-stream flow rate, thermal stability, and other stressors (Lytle and Poff 2004). When altered by the forces of climate change (e.g., lengthening seasons) those environments may in turn affect phenotypes such as spawn timing or migration timing (Crozier et al. 2011; Reed et al. 2011). Nevertheless, it cannot be stated unequivocally that climate associations are indicative of loci under direct selection.

Global climate change and effects of climate on ecosystems has earned the attention of the scientific community and freshwater fish are expected to be negatively impacted (McCullough et al. 2009). There is growing consensus that habitats occupied by salmonid species in North America and Europe will or have already experienced climate related alterations (Crozier and Zabel 2006; Battin et al. 2007; Winfield et al. 2010; Nielsen et al. 2013). Most of the CRB has been identified as habitat at high risk of thermal stress in salmonids (Wu et al. 2012), and conservation of many populations is already warranted (Busby et al. 1996; ICTRT 2003; USOFR 2006). Climate-altered habitats may lead to rapid evolution (Barrett et al. 2010) or shifting range margins of myriad species (Hickling et al. 2005; Chen et al. 2011). In fishes, this may manifest as range contractions in cold environments or expansions in warm environments, and adaptive versus neutral genetic divergence is likely to occur at differing spatial and temporal scales (Conover et al. 2006).

Therefore, conservation strategies based heavily on neutral genetic variation, with an under-emphasis on the distribution of non-neutral variation, risk detrimental impacts to locally adapted population segments and should be scrutinized (Pearman 2001; Schwartz et al. 2009). We concur with Funk et al. (2012) that neutral and non-neutral elements of differentiation are not mutually exclusive and should be used in concert to provide a cautious and conscientious description of species diversity in a management framework. Monitoring trends between neutral and non-neutral differentiation and the corresponding degree of disparity observed over time may be fundamentally important for addressing impacts of climate change (i.e., adaptive responses). This will conceivably have a potential role in reshaping conservation boundaries to safeguard species diversity. The design of our steelhead trout study follows this perspective; identifying selection candidate loci and environmental associations, then drawing comparisons with patterns of neutral population divergence. We observed non-neutral genetic heterogeneity of populations in association with environment. However, in our results, neutral diversity encapsulated overall steelhead trout diversity with better clarity and finer resolution across established DPSs. Nevertheless, monitoring of historical and/or contemporary non-neutral differentiation of populations adds a unique dimension to the characterization of these populations (Willing et al. 2010). The candidate markers identified in our study are expected to be useful for modeling population level responses to climate change, and future population genomics approaches with thousands of steelhead SNPs should provide improved estimation and resolution of adaptive differentiation in the CRB.

There is a palpable consensus among researchers and managers cautioning against broad scale, rigid approaches to conservation, and acknowledging the need to address productivity limitations of myriad organisms in need of protection (Clostio et al. 2012). However, conflicting opinions on the role of genetics in conservation still pervade management agendas (Waples 1995; Fraser and Bernatchez 2001; Garcia de Leaniz et al. 2007; Allendorf et al. 2010). Maintaining overall conservation unit viability must necessarily account for the viability of all demographically important population components within those units (Crandall et al. 2000; Latch et al. 2011), particularly where demographic instability (e.g., genetic drift in small populations) may reduce overall adaptive variation or adaptive potential within regions (Kawecki and Ebert 2004; Schoville et al. 2012). It is likely that the relevance and contribution of non-neutral variation is frequently overlooked or underutilized in conservation planning, but given recent calls for the incorporation of climate science in application of the ESA (e.g., McClure et al. 2013), the utility of such information should be highlighted. In the absence of efforts to regularly evaluate putatively adaptive population differences, there is presumably a greater risk of the loss of genetic diversity as climates and habitats continue to change through time. Over the long-term, the adaptive potential of many species across taxa will need to be further explored and considered in conservation planning. The real effects of a changing environment, including shifting ranges, may not be uniformly realized or fit tightly into predefined units (e.g., ESU, DPS, MPG).

## Data archiving statement

Data for this study are available from the Dryad Digital Repository: http://dx.doi.org/10.5061/dryad.ds6c4

## Supporting Information

**Table S1.** Collection names and reference numbers, DPS, and MPG, for 145 steelhead trout populations. **Table S2**. List of 191 SNP markers assayed for *O. mykiss* in the Columbia River Basin. **Table S3**. List of 191 SNP markers assayed for *O. mykiss* in the Columbia River Basin. **Table S4**. Values for predictor variables (by population) to control for underlying population structure in association tests (i.e., neutral variation). **Table S5**. Values for environmental predictor variables used in association tests to evaluate landscape genetics (see Table S6 and S7). **Table S6**. List of final locus classifications based on significance in association tests. **Table S7**. List of final locus classifications based on significance in association tests. The list includes only loci that met at least one of the four candidate criteria (see methods section), and neutral loci listed are only those that were initially flagged as possible candidates.

## References

[b1] Abadía-Cardoso A, Clemento AJ, Garza JC (2011). Discovery and characterization of single-nucleotide polymorphisms in steelhead/rainbow trout *Oncorhynchus mykiss*. Molecular Ecology Resources.

[b2] Ackerman M, Campbell M (2012). https://pisces.bpa.gov/release/documents/documentviewer.aspx?doc=P128035.

[b3] Ackerman MW, Templin WD, Seeb JE, Seeb LW (2012). Landscape heterogeneity and local adaptation define the spatial genetic structure of Pacific salmon in a pristine environment. Conservation Genetics.

[b4] Aguilar A, Garza JC (2008). Isolation of 15 single nucleotide polymorphisms from coastal steelhead, *Oncorhynchus mykiss* (Salmonidae). Molecular Ecology Resources.

[b5] Akey JM (2009). Constructing genomic maps of positive selection in humans: where do we go from here?. Genome Research.

[b6] Allendorf FW, Hohenlohe PA, Luikart G (2010). Genomics and the future of conservation genetics. Nature Reviews Genetics.

[b7] Anderson MJ, ter Braak CJF (2003). Permutation tests for multi-factorial analysis of variance. Journal of Statistical Computation and Simulation.

[b8] Anderson MJ, Ford RB, Feary DA, Honeywill C (2004). Quantitative measures of sedimentation in an estuarine system and its relationship with intertidal soft-sediment infauna. Marine Ecological-Progress Series.

[b9] Balkenhol N, Waits LP, Dezzani RJ (2009). Statistical approaches in landscape genetics: an evaluation of methods for linking landscape and genetic data. Ecography.

[b10] Barrett RD, Paccard A, Healy TM, Bergek S, Schulte PM, Schluter D, Rogers SM (2010). Rapid evolution of cold tolerance in stickleback. Proceedings of the Royal Society B: Biological Sciences.

[b11] Battin J, Wiley MW, Ruckelshaus MH, Palmer RN, Korb E, Bartz KK, Imaki H (2007). Projected impacts of climate change on salmon habitat restoration. Proceedings of the National Academy of Sciences.

[b12] Beacham TD, Pollard S, Le KD (1999). Population structure and stock identification of steelhead in southern British Columbia, Washington, and the Columbia River based on microsatellite DNA variation. Transactions of the American Fisheries Society.

[b13] Beaumont MA (2005). Adaptation and speciation: what can F_st_ tell us?. Trends in Ecology and Evolution.

[b14] Beaumont MA, Balding DJ (2004). Identifying adaptive genetic divergence among populations from genome scans. Molecular Ecology.

[b15] Beaumont MA, Nichols RA (1996). Evaluating loci for use in the genetic analysis of population structure. Proceedings of the Royal Society Biological Sciences.

[b16] Behnke RJ (2002). Trout and Salmon of North America.

[b17] Benjamini Y, Yekutieli D (2001). The control of false discovery rate under dependency. Annals of Statistics.

[b18] Blankenship SM, Campbell MR, Hess JE, Hess MA, Kassler TW, Kozfkay CC, Matala AP (2011). Major lineages and metapopulations in Columbia River *Oncorhynchus mykiss* are structured by dynamic landscape features and environments. Transactions of the American Fisheries Society.

[b20] Bonin A, Taberlet P, Miaud C, Ponpanon F (2009). Exploratory genome scan to detect candidate loci for adaptation along a gradient of altitude in the common frog (*Rana temporaria*. Molecular Biology and Evolution.

[b21] Bourret V, Dionne M, Kent MP, Lien S, Bernatchez L (2013). Landscape genomics in Atlantic salmon (*Salmo saloar*): searching for gene-environment interactions driving local adaptation. Evolution.

[b22] Bradshaw WE, Holzapfel CM (2008). Genetic responses to rapid climate change: it's seasonal timing that matters. Molecular Ecology.

[b23] Brunelli JP, Wertzler KJ, Sundin K, Thorgaard GH (2008). Y-specific sequences and polymorphisms in rainbow trout and Chinook salmon. Genome.

[b24] Busby PJ, Wainwright TC, Bryant GJ, Lierheimer LJ, Waples RS, Waknitz FW, Lagomarsino IV (1996). http://www.nwr.noaa.gov/1salmon/salmesa/pubs.htm.

[b26] Campbell NR, Narum SR (2009). Identification and characterization of heat shock response-reltaed single-nucleotide polymorphisms in *O. mykiss* and *O. tshawytscha*. Molecular Ecology Resources.

[b27] Campbell NR, Overturf K, Narum SR (2009). Characterization of 22 novel single nucleotide polymorphism markers in steelhead and rainbow trout. Molecular Ecology Resources.

[b28] Campbell NR, Amish SJ, Pritchard VL, MCKelvey KM, Young MK, Schwartz MK, Garza JC (2012). Development and evaluation of 200 novel SNP assays for population genetic studies of westslope cutthroat trout and genetic identification of related taxa. Molecular Ecology Resources.

[b29] Carmichael LE, Krizan J, Nagy JA, Fuglei E, Dumond M, Johnson D, Veitch A (2007). Historical and ecological determinants of genetic structure in arctic candies. Molecular Ecology.

[b30] Chen I-C, Hill JK, Ohlemüller R, Roy DB, Thomas CD (2011). Rapid range shifts of species associated with high levels of climate warming. Science.

[b31] Chilcote MW (1998). Conservation Status of Steelhead in Oregon.

[b32] Chilcote MW, Leider SA, Loch JJ (1986). Differential reproductive success of hatchery and wild summer-run steelhead under natural conditions. Transactions of the American Fisheries Society.

[b33] Clostio RW, Martinez AM, LeBlanc KE, Anthony NM (2012). Population genetic structure of a threatened tortoise across the south-eastern United States: implications for conservation management. Animal Conservation.

[b34] Coleman MA, Fausch KD (2007). Cold summer temperature limits recruitment of age-0 cutthroat trout in high-elevation Colorado streams. Transactions of the American Fisheries Society.

[b35] Conover DO, Clarke LM, Munch SB, Wagner GN (2006). Spatial and temporal scales of adaptive divergence in marine fishes and the implications for conservation. Journal of Fish Biology.

[b200] Crandall KA, Binindaemonds ORP, Mace GM, Wayne RK (2000). Considering evolutionary processes in conservation biology. Trends in Ecology and Evolution.

[b36] Crozier LG, Zabel RW (2006). Climate impacts at multiple scales: evidence for differential population responses in juvenile Chinook salmon. Journal of Animal Ecology.

[b37] Crozier LG, Scheuerell MD, Zabel RW (2011). Using time series analysis to characterize evolutionary and plastic responses to environmental change: a case study of a shift toward earlier migration date in sockeye salmon. American Naturalist.

[b38] Currens KP, Schreck CB, Li HW (2009). Evolutionary ecology of redband trout. Transactions of the American Fisheries Society.

[b39] Dionne M, Caron F, Dodson JJ, Bernatchez L (2008). Landscape genetics and hierarchical genetic structure in Atlantic salmon: the interaction of gene flow and local adaptation. Molecular Ecology.

[b40] Evanno G, Regnaut S, Goudet J (2005). Detecting the number of clusters of individuals using the software STRUCTURE: a simulation study. Molecular Ecology.

[b41] Excoffier L, Laval G, Schneider S (2005). Arlequin ver. 3.0: an integrated software package for population genetics data analysis. Evolutionary Bioinformatics Online.

[b42] Farrell AP, Hinch SG, Cooke SJ, Patterson DA, Crossin GT, Lapointe M, Mathes MT (2008). Pacific salmon in hot water: applying aerobic scope models and biotelemetry to predict the success of spawning migrations. Physiological and Biochemical Zoology.

[b43] Felsenstein J (2008). PHYLIP (Phylogeny Inference Package).

[b44] Ford MJ (2011).

[b300] Fourcade Y, Chaput-Bardy A, Secondi J, Fleurant C, Lemaire C (2013). Is local selection so widespread in river systems? Fractal geometry of river networks leads to high bias in outlier detection. Molecular Ecology.

[b45] Fowler SL, Hamilton D, Currie S (2009). A comparison of the heat shock response in juvenile and adult rainbow trout (*Oncorhynchus mykiss*) – implications for increased thermal sensitivity with age. Canadian Journal of Fisheries and Aquatic Sciences.

[b46] Fraser DJ, Bernatchez L (2001). Adaptive evolutionary conservation: towards a unified concept for defining conservation units. Molecular Ecology.

[b47] Fraser DJ, Weir LK, Bernatchez L, Hansen MM, Taylor EB (2011). Extent and scale of local adaptation in salmonid fishes: review and meta-analysis. Heredity.

[b48] Fryer JK, Whiteaker J, Kelsey D (2012).

[b49] Funk WC, McKay JK, Hohenlohe PA, Allendorf FW (2012). Harnessing genomics for delineating conservation units. Trends in Ecology and Evolution.

[b50] Garcia de Leaniz C, Fleming IA, Einum S, Verspoor E, Jordan WC, Consuegra S, Aubin-Horth N (2007). A critical review of adaptive genetic variation in Atlantic salmon: implications for conservation. Biological Reviews.

[b51] Ge S, Hong D-Y, Wang H-Q, Liu Z-Y, Zhang C-M (1998). Population genetic structure and conservation of an endangered conifer, *Cathaya argyrophylla* (Pinaceae). International Journal of Plant Sciences.

[b52] Good TP, Waples RS, Adams P (2005).

[b53] Goode JR, Buffington JM, Tonina D, Isaak DJ, Thurow RF, Wenger S, Nagel D (2013). Potential effects of climate change on streambed scour and risks to salmonid survival in snow-dominated mountain basins. Hydrological Processes.

[b54] Hansen MH, Young S, Jorgensen HBH, Pascal C, Henryon M, Seeb J (2011). Assembling a dual purpose TaqMan-based panel of single-nucleotide polymorphism markers in rainbow trout and steelhead (*Oncorhynchus mykiss*) for association mapping and population genetics analysis. Molecular Ecology Resources.

[b55] Hari RE, Livingstone DM, Siber R, Burkhardt-Holm P, Guttinger H (2006). Consequences of climatic change for water temperature and brown trout populations in Alpine rivers and streams. Global Change Biology.

[b56] Hendry MA, Wenburg JK, Myers KW, Hendry AP (2002). Genetic and phenotypic variation through the migratory season provides evidence for multiple populations of wild steelhead in the Dean River, British Columbia. Transactions of the American Fisheries Society.

[b57] Hermisson J (2009). Who believes in whole-genome scans for selection?. Heredity.

[b58] Hess JE, Campbell NR, Matala AP, Narum SR (2011).

[b59] Hess JE, Campbell NR, Matala AP, Narum SR (2012).

[b400] Hess JE, Narum SR (2011). Single-nucleotide polymorhphism (SNP) loci correlated with run timing in adult Chinook salmon from the Columbia River Basin. Transactions of the American Fisheries Society.

[b60] Hickling R, Roy DB, Hill JK, Thomas CD (2005). A northward shift of range margins in British Odonata. Global Change Biology.

[b61] Hoffmann AA, Sgrò CM (2011). Climate change and evolutionary adaptation. Nature.

[b500] Hohenlohe PA, Bassham S, Etter PD, Stiffler N, Johnson EA, Cresko WA (2010). Population genomics of parallel adaptation in threespine stickleback using sequenced RAD tags. PLoS genetics.

[b62] Holderegger R, Wagner HH (2008). Landscape genetics. BioScience.

[b63] ICTRT (Interior Columbia Technical Recovery Team) (2003).

[b64] Isaak DJ, Muhlfeld CC, Todd AS, Al-Chokhachy R, Roberts J, Kershner JL, Fausch KD (2012a). The past as prelude to the future for understanding 21st-century climate effects on Rocky Mountain trout. Fisheries.

[b65] Isaak DJ, Wollrab S, Horan D, Chandler G (2012b). Climate change effects on stream and river temperatures across the northwest U.S. from 1980-2009 and implications for salmonid fishes. Climate Change.

[b66] Jakobsson M, Rosenberg NA (2007). CLUMPP: a cluster matching and permutation program for dealing with label switching and multimodality in analysis of population structure. Bioinformatics.

[b67] Kassahn KS, Crozier RH, Ward AC, Stone G, Caley MJ (2007). From transcriptome to biological function: environmental stress in an ectothermic vertebrate, the coral reef fish *Pomacentrus moluccensis*. BMC Genomics.

[b68] Kawecki TJ, Ebert D (2004). Conceptual issues in local adaptation. Ecology Letters.

[b69] Kovach RP, Gharrett AJ, Tallmon DA (2012). Genetic change for earlier migration timing in a pink salmon population. Proceedings of the Royal Society.

[b70] LaHood ES, Miller JJ, Apland C, Ford MJ (2008). A rapid, ethanol-free fish tissue collection method for molecular genetic analyses. Transactions of the American Fisheries Society.

[b71] Landguth EL, Balkenhol N (2012). Relative sensitivity of neutral versus adaptive genetic data for assessing population differentiation. Conservation Genetics.

[b72] Latch EK, Boarman WI, Walde A, Fleischer RC (2011). Fine-scale analysis reveals cryptic landscape genetic structure in desert tortoises. PLoS One.

[b73] Limborg MT, Blankenship SM, Young SF, Utter FM, Seeb LW, Hansen MHH, Seeb JE (2011). Signatures of natural selection among lineages and habitats in *Oncorhynchus mykiss*. Ecology and Evolution.

[b74] Lin L-H, Qu Y-F, Li H, Zhou K-Y, Ji X (2012). Genetic structure and demographic history should inform conservation: Chinese cobras currently treated as homogenous show population divergence. PLoS One.

[b75] Loarie SR, Duffy PB, Hamilton H, Asner GP, Field CB, Ackerly DD (2009). The velocity of climate change. Nature.

[b76] Lytle DA, Poff NL (2004). Adaptation to natural flow regimes. Trends in Ecology and Evolution.

[b77] Manel S, Schwartz MK, Luikart G, Taberlet P (2003). Landscape genetics: combining landscape ecology and population genetics. Trends in Ecology and Evolution.

[b78] Matala AP, Marx S, Wise TG (2008). A genetically distinct wild redband trout (*Oncorhynchus mykiss gairdneri*) population in Crane Prairie Reservoir, Oregon, persists despite extensive stocking of hatchery rainbow trout (*O. m. irideus*. Conservation Genetics.

[b79] Matala AP, French R, Olsen E, Ardren WR (2009). Ecotype distinctions among steelhead in Hood River, Oregon, allow real-time genetic assignment of conservation broodstocks. Transactions of the American Fisheries Society.

[b80] Matala AP, Hess JE, Narum SR (2011). Resolving adaptive and demographic divergence among Chinook salmon populations in the Columbia River basin. Transactions of the American Fisheries Society.

[b81] McArdle BH, Anderson MJ (2001). Fitting multivariate models to community data: a comment on distance-based redundancy analysis. Ecology.

[b82] McClure MM, Alexander M, Borggaard D, Boughton D, Crozier L, Griffis R, Jorgensen JC (2013). Incorporating climate science in applications of the U.S. Endangered Species Act for aquatic species. Conservation Biology.

[b83] McCullough DA, Bartholow JM, Jager HI, Beschta RL, Cheslak EF, Deas ML, Ebersole JL (2009). Research in thermal biology: burning questions for coldwater stream fishes. Reviews in Fisheries Science.

[b84] Merila J, Hendry A (2014). Climate change, adaptation, and phenotypic plasticity: the problem and the evidence. Evolutionary Applications.

[b85] Milner A, Robertson AL, Monaghan KA, Veal AJ, Flory EA (2008). Colonization and development of an Alaskan stream community over 28 years. The Ecological Society of America.

[b600] De Mita S, Celine Thuillet A, Gay L, Ahmadi N, Manel S, Ronfort J, Vigouroux Y (2013). Detecting selection along environmental gradients: analysis of eight methods and their effectiveness for outbreeding and selfing populations. Molecular Ecology.

[b700] Moran P, Teel DJ, Banks MA, Beacham TD, Bellinger MR, Blankenship SM, Candy JR (2013). Divergent life-history races do not represent Chinook salmon coast-wide: the importance of scale in Quaternary biogeography. Canadian Journal of Fisheries and Aquatic Sciences.

[b86] Narum SR, Hess J (2011). Comparison of FST outlier tests for SNP loci under selection. Molecular Ecology Resources.

[b87] Narum SR, Zendt J, Graves D, Sharp B (2008). Influence of landscape on resident and anadromous life history types of *Oncorhynchus mykiss*. Canadian Journal of Fisheries and Aquatic Sciences.

[b88] Narum SR, Campbell NR, Kozfkay CC, Meyer KA (2010a). Adaptation of redband trout in desert and montane environments. Molecular Ecology.

[b89] Narum SR, Campbell N, Matala AP, Hess JE (2010b). https://pisces.bpa.gov/release/documents/documentviewer.aspx?doc=P115281.

[b90] Narum SR, Hess JE, Matala AP (2010c). Examining genetic lineages of Chinook salmon in the Columbia River Basin. Transactions of the American Fisheries Society.

[b91] Narum SR, Zendt JS, Frederiksen C, Campbell N, Matala A, Sharp W (2011). Candidate genetic markers associated with anadromy in *Oncorhynchus mykiss* of the Klickitat River. Transactions of the American Fisheries Society.

[b92] Narum SR, Campbell NR, Meyer KA, Miller MR, Hardy RW (2013). Thermal adaptation and acclimation of ectotherms from differing aquatic climates. Molecular Ecology.

[b93] Nei M (1972). Genetic distance between populations. The American Naturalist.

[b94] Nielsen JL, Byrne A, Graziano SL, Kozfkay CC (2009). Steelhead genetic diversity at multiple spatial scales in a managed basin: Snake River, Idaho. North American Journal of Fisheries Management.

[b95] Nielsen JL, Ruggerone GT, Zimmerman CE (2013). Adaptive strategies and life cycle characteristics in a warming climate: salmon in the Arctic?. Environmental Biology of Fishes.

[b96] Nosil P, Egan SP, Funk DJ (2007). Heterogeneous genomic differentiation between walking-stick ecotypes: “isolation by adaptation” and multiple roles for divergent selection. Evolution.

[b97] Olsen JB, Beacham TD, Wetklo M, Seeb LW, Smith CT, Flannery BG, Wenburg JK (2010). The influence of hydrology and waterway distance on population structure of Chinook salmon *Oncorhynchus tshawytscha* in a large river. Journal of Fish Biology.

[b98] Olsen JB, Crane PA, Flannery BG, Dunmall K, Templin WD, Wenburg JK (2011). Comparative landscape genetic analysis of three Pacific salmon species from subarctic North America. Conservation Genetics.

[b99] Page RDM (1996). TREEVIEW: an application to display phylogenetic trees on personal computers. Computer Applications in the Biosciences.

[b100] Peakall R, Smouse PE (2006). GENALEX 6: genetic analysis in Excel. Population genetic software for teaching and research. Molecular Ecology Notes.

[b101] Pearman PB (2001). Conservation value of independently evolving units: sacred cow or testable hypothesis?. Conservation Biology.

[b102] Perry GML, Danzmann RG, Ferguson MM, Gibson JP (2001). Quantitative trait loci for upper thermal tolerance in outbred strains of rainbow trout (*Oncorhynchus mykiss*. Heredity.

[b103] Pritchard J, Stephens M, Donnelly P (2000). Inference of population structure using multilocus genotype data. Genetics.

[b105] Raymond M, Rousset F (1995). GENEPOP (version 1.2): population genetics software for exact tests and ecumenicism. Journal of Heredity.

[b106] Reed TE, Schindler D, Hague M, Patterson D, Meir E, Waples RS, Hinch S (2011). Time to evolve? Potential evolutionary responses of Fraser River sockeye salmon to climate change and effects on persistence. PLoS One.

[b107] Rice WR (1989). Analyzing tables of statistical tests. Evolution.

[b108] Rieman BE, Isaak D, Adams S, Horan D, Nagel D, Luce C, Myers D (2007). Anticipated climate warming effects on bull trout habitats and populations across the interior Columbia River Basin. Transactions of the American Fisheries Society.

[b109] Ruokonen M, Kvist L, Aarvak T, Markkola J, Morozov VV, Oien IJ, Syroechkovsky EE (2004). Population genetic structure and conservation of the lesser white-fronted goose *Anser erythropus*. Conservation Genetics.

[b110] Sanchez CC, Smith TPL, Wiedmann RT, Vallejo RL, Salem M, Yao J, Rexroad CE (2009). Single nucleotide polymorphism discovery in rainbow trout by deep sequencing of a reduced representation library. BMC Genomics.

[b111] Schoville SD, Bonin A, Francois O, Lobreaux S, Melodelima C, Manel S (2012). Adaptive genetic variation on the landscape: methods and cases. Annual Reviews of Ecology Evolution and Systematics.

[b112] Schwartz MK, Luikart G, McKelvey KS, Cushman SA, Cushman SA, Huettmann F (2009). Landscape genomics: a brief perspective. Spatial Complexity, Informatics, and Wildlife Conservation.

[b113] Scott JB, Gill WT (2008). Oncorhynchus mykiss: Assessment of Washington State's Anadromous Populations and Programs.

[b114] Segelbacher G, Cushman SA, Epperson BK, Fortin MJ, Francois O, Hardy OJ, Holderegger R (2010). Applications of landscape genetics in conservation biology: concepts and challenges. Conservation Genetics.

[b115] Sepulveda-Villet OJ, Stepien CA (2012). Waterscape genetics of the yellow perch (*Perca flavescens*): patterns across large connected ecosystems and isolated relict populations. Molecular Ecology.

[b116] Sprowles AE, Stephens MR, Clipperton NW, May BP (2006). Fishing for SNPs: a targeted locus approach for single nucleotide polymorphism discovery in rainbow trout. Transactions of the American Fisheries Society.

[b117] Underwood KD, Ackerman NK, Chapman CG, Witty KL, Cramer SP, Hughes ML (2003). Hood River Production Program Review 1991–2001.

[b118] USOFR (U.S. Office of the Federal Register) (2006). Endangered and threatened species: final listing determinations for 10 distinct population segments of West Coast steelhead. Federal Register.

[b119] Wagner RS, Miller MP, Crisafulli CM, Halg SM (2005). Geographic variation, genetic structure, and conservation unit designation in the Larch Mountain salamander (*Plethodon larselli*. Canadian Journal of Zoology.

[b120] Waples RS (1995). Evolutionarily significant units and the conservation of biological diversity under the Endangered Species Act. American Fisheries Society Symposium.

[b121] Weir BS, Cockerham CC (1984). Estimating F-statistics for the analysis of population structure. Evolution.

[b122] Wenger SJ, Isaak DJ, Luce CH, Neville HM, Fausch KD, Dunham JB, Dauwalter DC (2011). Flow regime, temperature, and biotic interactions drive differential declines of trout species under climate change. Proceedings of the National Academy of Sciences.

[b123] Willing E-M, Bentzen P, Van Oosterhout C, Hoffmann M, Cable J, Breden F, Weigel D (2010). Genome-wide single nucleotide polymorphisms reveal population history and adaptive divergence in wild guppies. Molecular Ecology.

[b124] Winans GA, Paquin MM, Van Doornik DM (2004). Genetic stock identification of steelhead in the Columbia River basin: an evaluation of different molecular markers. North American Journal of Fisheries Management.

[b125] Winfield IJ, Hateley J, Fletcher JM, James JB, Bean CW, Clabburn P (2010). Clabburn. Population trends of Arctic charr (*Salvelinus alpinus*) in the UK: assessing the evidence for a widespread decline in response to climate change. Hydrobiologia.

[b126] Wu H, Kimball JS, Elsner MM, Mantua N, Adler RF, Stanford J (2012). Projected climate change impacts on the hydrology and temperature of Pacific Northwest rivers. Water Resources Research.

[b127] Zimmerman CE, Reeves GH (2000). Population structure of sympatric anadromous and nonanadromous *Oncorhynchus mykiss*: evidence from spawning surveys and otolith microchemistry. Canadian Journal of Fisheries and Aquatic Sciences.

[b128] Zydlewski GB, Haro A, McCormick SD (2005). Evidence for cumulative temperature as an initiating and terminating factor in downstream migratory behavior of Atlantic salmon (*Salmo salar*) smolts. Canadian Journal of Fisheries and Aquatic Sciences.

