# Supplementary material for: Relative contributions of neutral and non-neutral genetic differentiation to inform conservation of steelhead trout across highly variable landscapes
Source: Evol Appl. 2014 Jun 27;7(6):682–701. doi: 10.1111/eva.12174 (PMC4105918; doi:10.1111/eva.12174)
Supplement: Supplementary file 1 — Table S1. Collection names and reference numbers, DPS, and MPG, for 145 steelhead trout populations. Table S2. List of 191 SNP markers assayed for O. mykiss in the Columbia River Basin. Table S3. List of 191 SNP markers assayed for O. mykiss in the Columbia River Basin. Table S4. Values for predictor variables (by population) to control for underlying population structure in association tests (i.e., neutral variation). Table S5. Values for environmental predictor variables used in association tests to evaluate landscape genetics (see Table S6 and S7). Table S6. List of final locus classifications based on significance in association tests. Table S7. List of final locus classifications based on significance in association tests. The list includes only loci that met at least one of the four candidate criteria (see methods section), and neutral loci listed are only those that were initially flagged as possible candidates. [file eva0007-0682-SD1.doc]

Table S1. Collection names and reference numbers, DPS, and MPG, for 145 steelhead trout populations. Lineage is coastal (C) or inland (In). Origin is natural/wild (N), and hatchery (H). Run type is winter-run (W), summer-run (S), or unknown. Stage refers to life history stage: adult (A), juvenile (J), and smolts (S). One population from the Washington coast was included for comparison (*). The Big White Salmon populations is currently listed in the Middle Columbia River DPS; however based on these analyses it was revealed to have greater similarity to the coastal lineage (‡), and was grouped with the lower Columbia River DPS populations. The pairwise F_ST_ and observed heterozygosity (H_o_) results are the mean values over 180 loci and specific to population.

|  |  |  |  |  |  |  |  |  |  |  |  |
| --- | --- | --- | --- | --- | --- | --- | --- | --- | --- | --- | --- |
| Collection | |  |  |  |  |  |  |  |  |  |  |
| Ref. # | Name | tributary/ region | DPS | **MPG | (n) | lineage | Origin | Run | Stage | H_o_ | F_ST_ |
|  |  |  |  |  |  |  |  |  |  |  |  |
| 1 | *Quinault R. | Quinault | Southwest WA | n/a | 89 | C | H | W | A | 0.286 | 0.065 |
| 2 | Clackamas R. | Clackamas | Lower Columbia | C | 92 | C | N | W | J/A | 0.324 | 0.042 |
| 3 | Eagle Cr. | Clackamas | Lower Columbia | C | 47 | C | N | W | A | 0.310 | 0.031 |
| 4 | N. F. Eagle Cr. | Clackamas | Lower Columbia | C | 43 | C | N | W | J/S | 0.324 | 0.031 |
| 5 | Skamania Stock | Clackamas | Lower Columbia | C | 59 | C | H | S | A | 0.297 | 0.052 |
| 6 | Little Rock/Mad Cr. | Willamette | Upper Willamette | W | 50 | C | N | U | J | 0.334 | 0.046 |
| 7 | N. F. Santiam/ Mad Cr. | Willamette | Upper Willamette | W | 39 | C | N | W | A | 0.314 | 0.060 |
| 8 | S. F. Santiam/ Wiley Cr. | Willamette | Upper Willamette | W | 93 | C | N | W | J/A | 0.311 | 0.053 |
| 9 | Canyon Cr. | Willamette/ West Side | Upper Willamette | W | 25 | C | N | U | J | 0.335 | 0.079 |
| 10 | Luckiamute Cr. | Willamette/ West Side | Upper Willamette | W | 26 | C | N | U | J | 0.301 | 0.068 |
| 11 | Willamina Cr. | Willamette/ West Side | Upper Willamette | W | 30 | C | N | U | J | 0.307 | 0.056 |
| 12 | Abernathy Cr. | Abernathy | Southwest WA | n/a | 164 | C | N | W | A | 0.306 | 0.035 |
| 13 | Coweeman R. | Coweeman | Lower Columbia | C | 45 | C | N | W | A | 0.312 | 0.032 |
| 14 | Cowlitz R. | Cowlitz | Lower Columbia | C | 94 | C | N | W | A | 0.302 | 0.047 |
| 15 | Germany Cr. | Germany | Southwest WA | n/a | 47 | C | N | W | S | 0.312 | 0.033 |
| 16 | Kalama R. | Kalama | Lower Columbia | C | 94 | C | N | S | A | 0.317 | 0.032 |
| 17 | Kalama R. | Kalama | Lower Columbia | C | 94 | C | N | W | A | 0.310 | 0.029 |
| 18 | E. F. Lewis R. | Lewis | Lower Columbia | C | 77 | C | N | W | A | 0.307 | 0.031 |
| 19 | N. F. Lewis R. | Lewis | Lower Columbia | C | 94 | C | N | W | A | 0.311 | 0.034 |
| 20 | Mill Cr. | Mill | Southwest WA | n/a | 43 | C | N | W | S | 0.307 | 0.031 |
| 21 | Still Cr. | Sandy | Lower Columbia | C | 28 | C | N | U | J | 0.311 | 0.030 |
| 22 | East Fork Hood R. | Hood | Lower Columbia | G | 52 | C | N | W | S | 0.338 | 0.033 |
| 23 | West Fork Hood R. | Hood | Lower Columbia | G | 35 | C | N | S | S | 0.322 | 0.034 |
| 24 | ‡Big White Salmon R. | Big White Salmon | Middle Columbia | CES | 78 | C | N | U | J/A | 0.353 | 0.073 |
|  |  |  |  |  |  |  |  |  |  |  |  |
| 25 | Bowman Cr. | Klickitat | Middle Columbia | CES | 48 | IN | N | S | J | 0.330 | 0.069 |
| 26 | Deadcanyon Cr. | Klickitat | Middle Columbia | CES | 34 | IN | N | S | J | 0.339 | 0.068 |
| 27 | Lower Summit Cr. | Klickitat | Middle Columbia | CES | 45 | IN | N | S | J | 0.338 | 0.048 |
| 28 | Lower Trout Cr. | Klickitat | Middle Columbia | CES | 48 | IN | N | S | J | 0.324 | 0.052 |
| 29 | Lower White Cr. | Klickitat | Middle Columbia | CES | 33 | IN | N | S | J | 0.342 | 0.053 |
| 30 | Snyder Cr. | Klickitat | Middle Columbia | CES | 47 | IN | N | S | J | 0.284 | 0.089 |
| 31 | Surveyor Cr. | Klickitat | Middle Columbia | CES | 39 | IN | N | S | J | 0.283 | 0.098 |
| 32 | Swale Cr. | Klickitat | Middle Columbia | CES | 48 | IN | N | S | J | 0.352 | 0.075 |
| 33 | Upper Trout Cr. | Klickitat | Middle Columbia | CES | 46 | IN | N | S | J | 0.229 | 0.148 |
| 34 | Lower Little Klickitat R. | Little Klickitat | Middle Columbia | CES | 46 | IN | N | S | J | 0.337 | 0.063 |
| 35 | Buckhollow Cr. | Deschutes | Middle Columbia | CES | 63 | IN | N | S | J | 0.315 | 0.028 |
| 36 | Mainstem Deschutes R. | Deschutes | Middle Columbia | CES | 61 | IN | N | S | J | 0.324 | 0.069 |
| 37 | Pelton Dam Trap | Deschutes | Middle Columbia | CES | 45 | IN | N | S | A | 0.314 | 0.025 |
| 38 | Shitike Cr. | Deschutes | Middle Columbia | CES | 31 | IN | N | S | A | 0.308 | 0.028 |
| 39 | Trout Cr. | Deschutes | Middle Columbia | CES | 57 | IN | N | S | J | 0.309 | 0.026 |
| 40 | Fifteen Cr. | Fifteen | Middle Columbia | CES | 91 | IN | N | W | J | 0.334 | 0.036 |
| 41 | Baldy Cr. | John Day | Middle Columbia | JD | 25 | IN | N | S | J | 0.282 | 0.050 |
| 42 | Beech Cr. | John Day | Middle Columbia | JD | 21 | IN | N | S | J | 0.312 | 0.043 |
| 43 | Lower Mainstem J. D. | John Day | Middle Columbia | JD | 44 | IN | N | S | J | 0.308 | 0.027 |
| 44 | Upper Mainstem J. D. | John Day | Middle Columbia | JD | 34 | IN | N | S | J | 0.314 | 0.049 |
| 45 | Upper M. F. J. D. | M. F. John Day | Middle Columbia | JD | 107 | IN | N | S | J | 0.301 | 0.034 |
| 46 | Big Wall Cr. | N. F. John Day | Middle Columbia | JD | 22 | IN | N | S | J | 0.292 | 0.039 |
| 47 | Granite Cr. | N. F. John Day | Middle Columbia | JD | 18 | IN | N | S | J | 0.292 | 0.073 |
| 48 | Middle N. F. J. D. | N. F. John Day | Middle Columbia | JD | 56 | IN | N | S | J | 0.297 | 0.032 |
| 49 | Deer Cr. | S. F. John Day | Middle Columbia | JD | 18 | IN | N | S | J | 0.294 | 0.050 |
| 50 | Murderers Cr. | S. F. John Day | Middle Columbia | JD | 18 | IN | N | S | J | 0.303 | 0.041 |
| 51 | Rock Cr. | Rock | Middle Columbia | CES | 126 | IN | N | S | J | 0.317 | 0.029 |
| 52 | Squaw Cr. | Rock | Middle Columbia | CES | 138 | IN | N | S | J | 0.310 | 0.027 |
| 53 | Iskuulpa Cr. | Umatilla | Middle Columbia | UWW | 148 | IN | N | S | A/J | 0.312 | 0.028 |
| 54 | Umatilla R. | Umatilla | Middle Columbia | UWW | 34 | IN | N | S | J | 0.311 | 0.037 |
| 55 | Touchet R. | Walla Walla | Middle Columbia | UWW | 86 | IN | N | S | U | 0.293 | 0.036 |
| 56 | N. F. Little Naches R. | Yakima | Middle Columbia | Y | 21 | IN | N | S | J | 0.305 | 0.053 |
| 57 | Nile/ Naches R. | Yakima | Middle Columbia | Y | 59 | IN | N | S | J | 0.326 | 0.044 |
| 58 | Pileup/ Naches R. | Yakima | Middle Columbia | Y | 26 | IN | N | S | J | 0.306 | 0.050 |
| 59 | Quartz/ Naches R. | Yakima | Middle Columbia | Y | 26 | IN | N | S | J | 0.302 | 0.053 |
| 60 | Rattlesnake/ Naches R. | Yakima | Middle Columbia | Y | 36 | IN | N | S | J | 0.311 | 0.045 |
| 61 | Satus Cr. | Yakima | Middle Columbia | Y | 46 | IN | N | S | J | 0.298 | 0.046 |
| 62 | Toppenish Cr. | Yakima | Middle Columbia | Y | 44 | IN | N | S | J | 0.283 | 0.068 |
| 63 | Chiwaukum R. | Wenatchee | Upper Columbia | UCESC | 54 | IN | N | S | J | 0.313 | 0.032 |
| 64 | Iclicle Cr./ Wells Stock | Wenatchee | Upper Columbia | UCESC | 23 | IN | H | S | J | 0.313 | 0.038 |
| 65 | Leavenworth-NFH | Wenatchee | Upper Columbia | UCESC | 19 | IN | H | S | A | 0.307 | 0.032 |
| 66 | Nason Cr. | Wenatchee | Upper Columbia | UCESC | 21 | IN | N | S | J | 0.306 | 0.038 |
| 67 | Peshastin R. | Wenatchee | Upper Columbia | UCESC | 99 | IN | N | S | J | 0.304 | 0.027 |
| 68 | Upper Chiwaukum R. | Wenatchee | Upper Columbia | UCESC | 29 | IN | N | S | J | 0.310 | 0.032 |
| 69 | Entiat R. | Entiat | Upper Columbia | UCESC | 94 | IN | U | S | J | 0.311 | 0.029 |
| 70 | Methow R. | Methow | Upper Columbia | UCESC | 90 | IN | N | S | S | 0.309 | 0.029 |
| 71 | Bonaparte Cr. | Okanogan | Upper Columbia | UCESC | 99 | IN | U | S | J | 0.321 | 0.033 |
| 72 | Omak Cr. | Okanogan | Upper Columbia | UCESC | 94 | IN | N/H | S | A | 0.313 | 0.035 |
| 73 | Salmon Cr. | Okanogan | Upper Columbia | UCESC | 98 | IN | N/H | S | A | 0.336 | 0.043 |
| 74 | Tucannon R. | lower Snake | Snake | LS | 105 | IN | N | S | A | 0.307 | 0.025 |
| 75 | Alpowa Cr. | lower Snake | Snake | LS | 98 | IN | N | S | A | 0.306 | 0.026 |
| 76 | Asotin Cr. | lower Snake | Snake | LS | 98 | IN | N | S | A | 0.309 | 0.025 |
| 77 | Asotin Cr. | lower Snake | Snake | LS | 49 | IN | N | S | J | 0.313 | 0.025 |
| 78 | Captain John Cr. | lower Snake | Snake | LS | 56 | IN | N | S | J | 0.306 | 0.036 |
| 79 | George Cr. | lower Snake | Snake | LS | 95 | IN | N | S | A | 0.311 | 0.026 |
| 80 | Mission Cr. | Lapwai/ Clearwater | Snake | CL | 49 | IN | N | S | J | 0.302 | 0.031 |
| 81 | Big Bear Cr. | Lower Clearwater | Snake | CL | 98 | IN | N | S | A | 0.311 | 0.028 |
| 82 | E. F. Potlatch R. | Lower Clearwater | Snake | CL | 156 | IN | N | S | A | 0.300 | 0.034 |
| 83 | Little Bear Cr. | Lower Clearwater | Snake | CL | 151 | IN | N | S | A | 0.304 | 0.030 |
| 84 | W. F. Potlatch R. | Lower Clearwater | Snake | CL | 85 | IN | N | S | A | 0.301 | 0.032 |
| 85 | Bear/ Selway R. | M. F. Clearwater | Snake | CL | 35 | IN | N | S | J | 0.298 | 0.058 |
| 86 | Gedney/ Selway R. | M. F. Clearwater | Snake | CL | 45 | IN | N | S | J | 0.293 | 0.047 |
| 87 | Little Clearwater/ Selway R. | M. F. Clearwater | Snake | CL | 59 | IN | N | S | J | 0.288 | 0.055 |
| 88 | Mainstem Selway R. | M. F. Clearwater | Snake | CL | 76 | IN | N | S | J | 0.288 | 0.062 |
| 89 | N. F. Moose/ Selway R. | M. F. Clearwater | Snake | CL | 92 | IN | N | S | J | 0.285 | 0.049 |
| 90 | OHara/ Selway R. | M. F. Clearwater | Snake | CL | 47 | IN | N | S | J | 0.290 | 0.042 |
| 91 | Three Links/ Selway R. | M. F. Clearwater | Snake | CL | 47 | IN | N | S | J | 0.276 | 0.066 |
| 92 | Whitecap/ Selway R. | M. F. Clearwater | Snake | CL | 76 | IN | N | S | J | 0.295 | 0.060 |
| 93 | Canyon/ Lochsa R. | M. F. Clearwater | Snake | CL | 46 | IN | N | S | J | 0.285 | 0.052 |
| 94 | Colt/ Lochsa R. | M. F. Clearwater | Snake | CL | 38 | IN | N | S | J | 0.280 | 0.058 |
| 95 | Crooked Fork/Lochsa R. | M. F. Clearwater | Snake | CL | 44 | IN | N | S | J | 0.282 | 0.055 |
| 96 | Fish/ Lochsa R. | M. F. Clearwater | Snake | CL | 99 | IN | N | S | A | 0.279 | 0.053 |
| 97 | Lake/ Lochsa R. | M. F. Clearwater | Snake | CL | 47 | IN | N | S | J | 0.279 | 0.060 |
| 98 | Storm/ Lochsa R. | M. F. Clearwater | Snake | CL | 38 | IN | N | S | J | 0.281 | 0.064 |
| 99 | Clear Cr. | S. F. Clearwater | Snake | CL | 45 | IN | N | S | J | 0.276 | 0.051 |
| 100 | Crooked R. | S. F. Clearwater | Snake | CL | 104 | IN | N | S | A | 0.274 | 0.057 |
| 101 | Johns Cr. | S. F. Clearwater | Snake | CL | 36 | IN | N | S | J | 0.283 | 0.045 |
| 102 | Tenmile Cr. | S. F. Clearwater | Snake | CL | 46 | IN | N | S | J | 0.286 | 0.066 |
| 103 | Slate Cr. | Lower Salmon | Snake | S | 46 | IN | N | S | J | 0.304 | 0.030 |
| 104 | Whitebird Cr. | Lower Salmon | Snake | S | 59 | IN | N | S | J | 0.295 | 0.032 |
| 105 | Crooked Cr. | Grande Ronde | Snake | GR | 95 | IN | N | S | J | 0.298 | 0.031 |
| 106 | Elk Cr. | Grande Ronde | Snake | GR | 45 | IN | N | S | J | 0.289 | 0.047 |
| 107 | Joseph Cr. | Grande Ronde | Snake | GR | 45 | IN | N | S | A | 0.306 | 0.029 |
| 108 | Little Minam R. | Grande Ronde | Snake | GR | 48 | IN | N | S | J | 0.300 | 0.044 |
| 109 | Lostine R. | Grande Ronde | Snake | GR | 45 | IN | N | S | J | 0.306 | 0.040 |
| 110 | Menatchee R. | Grande Ronde | Snake | GR | 68 | IN | N | S | J | 0.319 | 0.032 |
| 111 | Wenaha R. | Grande Ronde | Snake | GR | 93 | IN | N | S | J | 0.292 | 0.030 |
| 112 | Big Sheep Cr. | Imnaha | Snake | I | 61 | IN | N | S | J | 0.292 | 0.036 |
| 113 | Camp Cr. | Imnaha | Snake | I | 23 | IN | N | S | J | 0.297 | 0.042 |
| 114 | Cow Cr. | Imnaha | Snake | I | 44 | IN | N | S | J | 0.298 | 0.032 |
| 115 | Lightning Cr. | Imnaha | Snake | I | 38 | IN | N | S | J | 0.288 | 0.035 |
| 116 | Boulder Cr. | Little Salmon | Snake | S | 47 | IN | N | S | J | 0.293 | 0.034 |
| 117 | Hazard Cr. | Little Salmon | Snake | S | 43 | IN | N | S | J | 0.309 | 0.028 |
| 118 | Rapid R. | Little Salmon | Snake | S | 99 | IN | H | S | A | 0.301 | 0.037 |
| 119 | East Fork S. F. Salmon R. | S. F. Salmon | Snake | S | 45 | IN | N | S | J | 0.302 | 0.057 |
| 120 | Lick Cr. | S. F. Salmon | Snake | S | 39 | IN | N | S | J | 0.284 | 0.051 |
| 121 | Secesh R. | S. F. Salmon | Snake | S | 45 | IN | N | S | J | 0.293 | 0.053 |
| 122 | Stolle Meadows | S. F. Salmon | Snake | S | 45 | IN | N | S | J | 0.285 | 0.055 |
| 123 | Chamberlain Cr. | Chamberlain | Snake | S | 46 | IN | N | S | J | 0.294 | 0.038 |
| 124 | Bargamin Cr. | Bargamin | Snake | S | 46 | IN | N | S | J | 0.309 | 0.042 |
| 125 | Camas Cr. | M. F. Salmon | Snake | S | 56 | IN | N | S | J | 0.287 | 0.048 |
| 126 | Loon Cr. | M. F. Salmon | Snake | S | 84 | IN | N | S | J | 0.285 | 0.051 |
| 127 | Lower Big Cr. | M. F. Salmon | Snake | S | 46 | IN | N | S | J | 0.288 | 0.049 |
| 128 | Marsh Cr. | M. F. Salmon | Snake | S | 59 | IN | N | S | J | 0.291 | 0.065 |
| 129 | Pistol Cr. | M. F. Salmon | Snake | S | 23 | IN | N | S | J | 0.304 | 0.064 |
| 130 | Rapid R. | M. F. Salmon | Snake | S | 31 | IN | N | S | J | 0.292 | 0.061 |
| 131 | Sulphur Cr. | M. F. Salmon | Snake | S | 42 | IN | N | S | J | 0.290 | 0.061 |
| 132 | Upper Big Cr. | M. F. Salmon | Snake | S | 45 | IN | N | S | J | 0.298 | 0.063 |
| 133 | Hayden Cr. | Upper Salmon | Snake | S | 84 | IN | N | S | J | 0.320 | 0.031 |
| 134 | Morgan Cr. | Upper Salmon | Snake | S | 37 | IN | N | S | J | 0.321 | 0.037 |
| 135 | N. F. Salmon R. | Upper Salmon | Snake | S | 99 | IN | N | S | A | 0.308 | 0.028 |
| 136 | Pahsimeroi Weir | Upper Salmon | Snake | S | 96 | IN | N | S | A | 0.307 | 0.034 |
| 137 | Sawtooth Weir | Upper Salmon | Snake | S | 105 | IN | N | S | A | 0.301 | 0.036 |
| 138 | Valley Cr. | Upper Salmon | Snake | S | 44 | IN | N | S | J | 0.307 | 0.035 |
| 139 | W. F. Yankee Fork R. | Upper Salmon | Snake | S | 117 | IN | N | S | J | 0.304 | 0.034 |
|  |  |  |  |  |  |  |  |  |  |  |  |
| 140 | Dworshak | Hatchery | Snake | CL | 114 | IN | H | S | A | 0.279 | 0.056 |
| 141 | Oxbow/ Hells Canyon | Hatchery | Snake | LS | 90 | IN | H | S | A | 0.304 | 0.039 |
| 142 | Pahsimeroi | Hatchery | Snake | S | 146 | IN | H | S | A | 0.320 | 0.035 |
| 143 | Sawtooth | Hatchery | Snake | S | 93 | IN | H | S | A | 0.298 | 0.039 |
| 144 | Tucannon/Lyons Ferry | Hatchery | Snake | LS | 89 | IN | H | S | A | 0.308 | 0.028 |
| 145 | Wallowa | Hatchery | Snake | GR | 93 | IN | H | S | A | 0.302 | 0.032 |
|  |  |  |  |  |  |  |  |  |  |  |  |
|  |  |  |  |  |  |  |  |  |  |  |  |

***Major Population Groups (MPG) are: W – Willamette, C – Cascade, G – Gorge, CES – Cascade Eastern Slope, JD – John Day, UWW – Umatilla and Walla Walla, Y – Yakima, UCESC - Upper Columbia / East Slope Cascades, LS – Lower Snake, S – Salmon,*

*CL – Clearwater, GR – Grande Ronde, I - Imnaha*

Table S2. List of 191 SNP markers assayed for *O. mykiss* in the Columbia River Basin. The minor allele frequency (MAF), locus-specific F_ST_, and observed heterozygosity (H_o_) are the mean values among all populations, with respect to lineage-of-origin.

|  |  |  |  |  |  |  |  |  |  |  |  |
| --- | --- | --- | --- | --- | --- | --- | --- | --- | --- | --- | --- |
|  | coastal MAF | |  | inland MAF | |  | coastal | inland |  | coastal | inland |
| SNP locus | mean | range |  | mean | range |  | F_ST_ | F_ST_ |  | H_o_ | H_o_ |
|  |  |  |  |  |  |  |  |  |  |  |  |
| Omy_97954-618 | 0.069 | 0.483 |  | 0.181 | 0.509 |  | 0.155 | 0.061 |  | 0.097 | 0.265 |
| Omy_gdh-271 | 0.173 | 0.313 |  | 0.104 | 0.390 |  | 0.035 | 0.052 |  | 0.270 | 0.172 |
| OMS00014 | 0.035 | 0.234 |  | 0.036 | 0.186 |  | 0.071 | 0.041 |  | 0.062 | 0.042 |
| OMS00062 | 0.280 | 0.240 |  | 0.256 | 0.426 |  | 0.022 | 0.033 |  | 0.402 | 0.381 |
| OMS00151 | 0.102 | 0.270 |  | 0.183 | 0.391 |  | 0.043 | 0.038 |  | 0.182 | 0.289 |
| Omy_97660-230 | 0.176 | 0.422 |  | 0.362 | 0.641 |  | 0.093 | 0.043 |  | 0.257 | 0.442 |
| Omy_CRBF1-1 | 0.241 | 0.410 |  | 0.083 | 0.297 |  | 0.058 | 0.050 |  | 0.324 | 0.129 |
| Omy_e1-147 | 0.204 | 0.470 |  | 0.076 | 0.264 |  | 0.069 | 0.054 |  | 0.292 | 0.128 |
| Omy_GHSR-121 | 0.142 | 0.462 |  | 0.081 | 0.317 |  | 0.069 | 0.063 |  | 0.228 | 0.114 |
| Omy_hsc715-80 | 0.386 | 0.309 |  | 0.413 | 0.517 |  | 0.030 | 0.039 |  | 0.493 | 0.461 |
| Omy_IL6-320 | 0.201 | 0.323 |  | 0.229 | 0.465 |  | 0.032 | 0.043 |  | 0.322 | 0.344 |
| Omy_metA-161 | 0.487 | 0.399 |  | 0.281 | 0.535 |  | 0.043 | 0.052 |  | 0.474 | 0.392 |
| Omy_nkef-241 | 0.328 | 0.311 |  | 0.455 | 0.614 |  | 0.033 | 0.051 |  | 0.418 | 0.475 |
| Omy_ntl-27 | 0.253 | 0.458 |  | 0.393 | 0.731 |  | 0.073 | 0.072 |  | 0.367 | 0.445 |
| Omy_SECC22b-88 | 0.051 | 0.301 |  | 0.045 | 0.204 |  | 0.103 | 0.063 |  | 0.092 | 0.040 |
| Omy_u09-53.469 | 0.251 | 0.275 |  | 0.372 | 0.771 |  | 0.037 | 0.099 |  | 0.388 | 0.418 |
| Omy_UT16_2-173 | 0.177 | 0.270 |  | 0.085 | 0.625 |  | 0.036 | 0.098 |  | 0.279 | 0.135 |
| OMY1011SNP | 0.465 | 0.332 |  | 0.293 | 0.626 |  | 0.018 | 0.055 |  | 0.516 | 0.383 |
| Omy_hsp47-86 | 0.366 | 0.342 |  | 0.234 | 0.364 |  | 0.025 | 0.030 |  | 0.458 | 0.350 |
| Omy_OmyP9-180 | 0.311 | 0.312 |  | 0.105 | 0.281 |  | 0.044 | 0.044 |  | 0.390 | 0.171 |
| Omy_stat3-273 | 0.368 | 0.288 |  | 0.236 | 0.434 |  | 0.015 | 0.038 |  | 0.487 | 0.350 |
| Omy_tlr5-205 | 0.236 | 0.460 |  | 0.085 | 0.265 |  | 0.041 | 0.050 |  | 0.344 | 0.145 |
| Omy_aldB-165 | 0.417 | 0.311 |  | 0.321 | 0.542 |  | 0.031 | 0.039 |  | 0.480 | 0.428 |
| Omy_hsf2-146 | 0.028 | 0.096 |  | 0.310 | 0.610 |  | 0.027 | 0.076 |  | 0.054 | 0.384 |
| OMS00008 | 0.032 | 0.128 |  | 0.179 | 0.456 |  | 0.024 | 0.067 |  | 0.060 | 0.268 |
| OMS00058 | 0.323 | 0.283 |  | 0.419 | 0.598 |  | 0.025 | 0.065 |  | 0.447 | 0.468 |
| OMS00111 | 0.118 | 0.213 |  | 0.188 | 0.508 |  | 0.025 | 0.062 |  | 0.205 | 0.281 |
| Omy_bcAKala-380rd | 0.267 | 0.561 |  | 0.341 | 0.618 |  | 0.104 | 0.060 |  | 0.328 | 0.425 |
| Omy_cox1-221 | 0.457 | 0.553 |  | 0.367 | 0.579 |  | 0.052 | 0.061 |  | 0.474 | 0.439 |
| Omy_anp-17 | 0.267 | 0.341 |  | 0.354 | 0.735 |  | 0.035 | 0.098 |  | 0.388 | 0.419 |
| Omy_b9-164 | 0.036 | 0.128 |  | 0.106 | 0.449 |  | 0.027 | 0.110 |  | 0.056 | 0.143 |
| Omy_IL1b-163 | 0.197 | 0.539 |  | 0.143 | 0.601 |  | 0.088 | 0.170 |  | 0.272 | 0.168 |
| Omy_nxt2-273 | 0.127 | 0.285 |  | 0.096 | 0.581 |  | 0.042 | 0.111 |  | 0.220 | 0.135 |
| Omy_star-206 | 0.467 | 0.475 |  | 0.097 | 0.601 |  | 0.068 | 0.090 |  | 0.484 | 0.143 |
| Omy_u09-56.119 | 0.475 | 0.431 |  | 0.149 | 0.654 |  | 0.054 | 0.142 |  | 0.471 | 0.224 |
| Omy_vatf-406 | 0.352 | 0.343 |  | 0.401 | 0.841 |  | 0.038 | 0.089 |  | 0.456 | 0.442 |
| Omy_LDHB-2_i6 | 0.092 | 0.214 |  | 0.032 | 0.223 |  | 0.036 | 0.051 |  | 0.161 | 0.034 |
| Omy_ndk-152 | 0.229 | 0.509 |  | 0.077 | 0.547 |  | 0.076 | 0.193 |  | 0.326 | 0.088 |
| Omy_Ogo4-212 | 0.319 | 0.641 |  | 0.442 | 0.639 |  | 0.108 | 0.050 |  | 0.404 | 0.472 |
| OMS00064 | 0.468 | 0.594 |  | 0.394 | 0.607 |  | 0.099 | 0.062 |  | 0.450 | 0.449 |
| OMS00096 | 0.268 | 0.614 |  | 0.218 | 0.461 |  | 0.116 | 0.042 |  | 0.351 | 0.327 |
| OMS00118 | 0.197 | 0.521 |  | 0.398 | 0.552 |  | 0.121 | 0.072 |  | 0.281 | 0.433 |
| OMS00174 | 0.049 | 0.237 |  | 0.048 | 0.167 |  | 0.112 | 0.027 |  | 0.073 | 0.078 |
| Omy_cd59-206 | 0.340 | 0.600 |  | 0.282 | 0.553 |  | 0.138 | 0.045 |  | 0.376 | 0.398 |
| Omy_metB-138 | 0.098 | 0.468 |  | 0.170 | 0.462 |  | 0.104 | 0.047 |  | 0.162 | 0.265 |
| M09AAC.055 | 0.033 | 0.096 |  | 0.085 | 0.322 |  | 0.028 | 0.055 |  | 0.065 | 0.137 |
| M09AAD.076 | 0.122 | 0.404 |  | 0.474 | 0.522 |  | 0.065 | 0.051 |  | 0.207 | 0.477 |
| M09AAE.082 | 0.394 | 0.433 |  | 0.252 | 0.656 |  | 0.049 | 0.086 |  | 0.465 | 0.343 |
| M09AAJ.163 | 0.435 | 0.262 |  | 0.303 | 0.547 |  | 0.029 | 0.053 |  | 0.510 | 0.400 |
| OMGH1PROM1-SNP1 | 0.099 | 0.375 |  | 0.114 | 0.375 |  | 0.063 | 0.075 |  | 0.190 | 0.180 |
| OMS00002 | 0.341 | 0.520 |  | 0.325 | 0.486 |  | 0.078 | 0.039 |  | 0.427 | 0.428 |
| OMS00003 | 0.316 | 0.378 |  | 0.157 | 0.402 |  | 0.042 | 0.039 |  | 0.424 | 0.255 |
| OMS00006 | 0.438 | 0.397 |  | 0.490 | 0.645 |  | 0.035 | 0.042 |  | 0.469 | 0.493 |
| OMS00013 | 0.479 | 0.388 |  | 0.099 | 0.406 |  | 0.062 | 0.071 |  | 0.493 | 0.154 |
| OMS00015 | 0.145 | 0.316 |  | 0.070 | 0.405 |  | 0.048 | 0.061 |  | 0.240 | 0.113 |
| OMS00017 | 0.315 | 0.452 |  | 0.296 | 0.667 |  | 0.063 | 0.067 |  | 0.411 | 0.380 |
| OMS00018 | 0.185 | 0.503 |  | 0.121 | 0.345 |  | 0.087 | 0.056 |  | 0.246 | 0.204 |
| OMS00024 | 0.265 | 0.478 |  | 0.359 | 0.686 |  | 0.055 | 0.060 |  | 0.409 | 0.435 |
| OMS00030 | 0.094 | 0.269 |  | 0.098 | 0.198 |  | 0.053 | 0.031 |  | 0.173 | 0.161 |
| OMS00039 | 0.369 | 0.438 |  | 0.475 | 0.691 |  | 0.044 | 0.044 |  | 0.494 | 0.497 |
| OMS00048 | 0.432 | 0.324 |  | 0.136 | 0.364 |  | 0.039 | 0.044 |  | 0.420 | 0.225 |
| OMS00052 | 0.170 | 0.287 |  | 0.192 | 0.353 |  | 0.041 | 0.033 |  | 0.277 | 0.290 |
| OMS00053 | 0.171 | 0.338 |  | 0.440 | 0.596 |  | 0.040 | 0.038 |  | 0.283 | 0.477 |
| OMS00056 | 0.291 | 0.415 |  | 0.217 | 0.409 |  | 0.048 | 0.034 |  | 0.399 | 0.336 |
| OMS00057 | 0.388 | 0.384 |  | 0.357 | 0.606 |  | 0.053 | 0.050 |  | 0.439 | 0.448 |
| OMS00061 | 0.289 | 0.305 |  | 0.081 | 0.235 |  | 0.024 | 0.055 |  | 0.409 | 0.121 |
| OMS00068 | 0.195 | 0.514 |  | 0.331 | 0.529 |  | 0.088 | 0.046 |  | 0.297 | 0.423 |
| OMS00070 | 0.395 | 0.376 |  | 0.497 | 0.672 |  | 0.044 | 0.060 |  | 0.439 | 0.479 |
| OMS00071 | 0.346 | 0.280 |  | 0.431 | 0.590 |  | 0.020 | 0.047 |  | 0.463 | 0.471 |
| OMS00072 | 0.493 | 0.346 |  | 0.445 | 0.553 |  | 0.046 | 0.035 |  | 0.487 | 0.473 |
| OMS00074 | 0.396 | 0.453 |  | 0.490 | 0.755 |  | 0.047 | 0.080 |  | 0.496 | 0.458 |
| OMS00077 | 0.333 | 0.429 |  | 0.406 | 0.748 |  | 0.054 | 0.049 |  | 0.443 | 0.469 |
| OMS00078 | 0.187 | 0.331 |  | 0.282 | 0.499 |  | 0.059 | 0.031 |  | 0.282 | 0.393 |
| OMS00079 | 0.429 | 0.310 |  | 0.483 | 0.710 |  | 0.029 | 0.049 |  | 0.466 | 0.481 |
| OMS00089 | 0.265 | 0.423 |  | 0.285 | 0.571 |  | 0.043 | 0.062 |  | 0.386 | 0.385 |
| OMS00090 | 0.334 | 0.462 |  | 0.460 | 0.607 |  | 0.044 | 0.059 |  | 0.421 | 0.471 |
| OMS00092 | 0.230 | 0.397 |  | 0.156 | 0.493 |  | 0.077 | 0.071 |  | 0.327 | 0.244 |
| OMS00095 | 0.002 | 0.029 |  | 0.064 | 0.251 |  | 0.019 | 0.042 |  | 0.004 | 0.105 |
| OMS00101 | 0.450 | 0.392 |  | 0.417 | 0.639 |  | 0.032 | 0.049 |  | 0.531 | 0.475 |
| OMS00105 | 0.323 | 0.327 |  | 0.397 | 0.580 |  | 0.028 | 0.052 |  | 0.431 | 0.468 |
| OMS00106 | 0.359 | 0.564 |  | 0.250 | 0.546 |  | 0.078 | 0.057 |  | 0.441 | 0.359 |
| OMS00112 | 0.109 | 0.343 |  | 0.183 | 0.444 |  | 0.048 | 0.050 |  | 0.196 | 0.288 |
| OMS00114 | 0.016 | 0.110 |  | 0.082 | 0.212 |  | 0.038 | 0.033 |  | 0.032 | 0.144 |
| OMS00119 | 0.230 | 0.446 |  | 0.144 | 0.398 |  | 0.068 | 0.050 |  | 0.325 | 0.236 |
| OMS00120 | 0.412 | 0.365 |  | 0.192 | 0.475 |  | 0.040 | 0.064 |  | 0.456 | 0.284 |
| OMS00121 | 0.400 | 0.377 |  | 0.472 | 0.604 |  | 0.032 | 0.041 |  | 0.508 | 0.489 |
| OMS00129 | 0.067 | 0.385 |  | 0.188 | 0.421 |  | 0.095 | 0.051 |  | 0.115 | 0.253 |
| OMS00132 | 0.419 | 0.499 |  | 0.413 | 0.608 |  | 0.053 | 0.039 |  | 0.457 | 0.484 |
| OMS00133 | 0.301 | 0.430 |  | 0.056 | 0.198 |  | 0.056 | 0.058 |  | 0.391 | 0.072 |
| OMS00138 | 0.059 | 0.214 |  | 0.117 | 0.380 |  | 0.052 | 0.051 |  | 0.110 | 0.197 |
| OMS00143 | 0.031 | 0.103 |  | 0.098 | 0.299 |  | 0.043 | 0.051 |  | 0.062 | 0.168 |
| OMS00149 | 0.122 | 0.376 |  | 0.064 | 0.319 |  | 0.081 | 0.049 |  | 0.197 | 0.101 |
| OMS00154 | 0.442 | 0.282 |  | 0.224 | 0.426 |  | 0.029 | 0.051 |  | 0.497 | 0.338 |
| OMS00173 | 0.045 | 0.138 |  | 0.128 | 0.302 |  | 0.059 | 0.040 |  | 0.084 | 0.218 |
| OMS00175 | 0.388 | 0.307 |  | 0.418 | 0.560 |  | 0.027 | 0.061 |  | 0.463 | 0.471 |
| OMS00179 | 0.497 | 0.529 |  | 0.307 | 0.477 |  | 0.050 | 0.042 |  | 0.471 | 0.407 |
| OMS00180 | 0.244 | 0.264 |  | 0.345 | 0.559 |  | 0.023 | 0.053 |  | 0.367 | 0.439 |
| Omy_101832-195 | 0.434 | 0.633 |  | 0.445 | 0.772 |  | 0.071 | 0.054 |  | 0.472 | 0.477 |
| Omy_101993-189 | 0.163 | 0.287 |  | 0.256 | 0.567 |  | 0.032 | 0.071 |  | 0.266 | 0.354 |
| Omy_102505-102 | 0.320 | 0.530 |  | 0.372 | 0.567 |  | 0.093 | 0.038 |  | 0.362 | 0.461 |
| Omy_103705-558 | 0.286 | 0.294 |  | 0.119 | 0.271 |  | 0.030 | 0.034 |  | 0.395 | 0.200 |
| Omy_104519-624 | 0.299 | 0.489 |  | 0.311 | 0.525 |  | 0.046 | 0.060 |  | 0.373 | 0.416 |
| Omy_105075-162 | 0.302 | 0.387 |  | 0.104 | 0.364 |  | 0.042 | 0.047 |  | 0.401 | 0.176 |
| Omy_105105-448 | 0.314 | 0.374 |  | 0.435 | 0.555 |  | 0.056 | 0.047 |  | 0.397 | 0.474 |
| Omy_105385-406 | 0.408 | 0.482 |  | 0.440 | 0.615 |  | 0.075 | 0.054 |  | 0.455 | 0.468 |
| Omy_105714-265 | 0.314 | 0.609 |  | 0.320 | 0.559 |  | 0.095 | 0.056 |  | 0.393 | 0.421 |
| Omy_107031-704 | 0.182 | 0.305 |  | 0.197 | 0.586 |  | 0.034 | 0.080 |  | 0.284 | 0.300 |
| Omy_107285-69 | 0.147 | 0.236 |  | 0.173 | 0.414 |  | 0.029 | 0.047 |  | 0.246 | 0.279 |
| Omy_107806-34 | 0.494 | 0.493 |  | 0.339 | 0.839 |  | 0.044 | 0.088 |  | 0.472 | 0.401 |
| Omy_108007-193 | 0.136 | 0.370 |  | 0.418 | 0.643 |  | 0.074 | 0.068 |  | 0.203 | 0.452 |
| Omy_109243-222 | 0.320 | 0.501 |  | 0.184 | 0.355 |  | 0.059 | 0.039 |  | 0.403 | 0.285 |
| Omy_109894-185 | 0.265 | 0.470 |  | 0.332 | 0.524 |  | 0.037 | 0.054 |  | 0.366 | 0.408 |
| Omy_110064-419 | 0.121 | 0.320 |  | 0.367 | 0.672 |  | 0.039 | 0.061 |  | 0.201 | 0.435 |
| Omy_110201-359 | 0.457 | 0.580 |  | 0.126 | 0.372 |  | 0.082 | 0.059 |  | 0.449 | 0.207 |
| Omy_111383-51 | 0.428 | 0.453 |  | 0.437 | 0.733 |  | 0.033 | 0.056 |  | 0.526 | 0.475 |
| Omy_113490-159 | 0.370 | 0.573 |  | 0.386 | 0.691 |  | 0.061 | 0.075 |  | 0.442 | 0.449 |
| Omy_114315-438 | 0.179 | 0.269 |  | 0.439 | 0.657 |  | 0.026 | 0.076 |  | 0.300 | 0.446 |
| Omy_114587-480 | 0.132 | 0.244 |  | 0.314 | 0.508 |  | 0.036 | 0.062 |  | 0.226 | 0.410 |
| Omy_116733-349 | 0.150 | 0.306 |  | 0.274 | 0.570 |  | 0.048 | 0.047 |  | 0.234 | 0.385 |
| Omy_128923-433 | 0.183 | 0.340 |  | 0.495 | 0.686 |  | 0.051 | 0.064 |  | 0.288 | 0.478 |
| Omy_128996-481 | 0.418 | 0.321 |  | 0.113 | 0.640 |  | 0.035 | 0.093 |  | 0.476 | 0.154 |
| Omy_129870-756 | 0.135 | 0.424 |  | 0.168 | 0.328 |  | 0.054 | 0.032 |  | 0.219 | 0.267 |
| Omy_130524-160 | 0.423 | 0.300 |  | 0.390 | 0.546 |  | 0.029 | 0.035 |  | 0.470 | 0.458 |
| Omy_97077-73 | 0.087 | 0.298 |  | 0.037 | 0.145 |  | 0.042 | 0.036 |  | 0.164 | 0.046 |
| Omy_97865-196 | 0.022 | 0.090 |  | 0.054 | 0.171 |  | 0.037 | 0.035 |  | 0.040 | 0.076 |
| Omy_99300-202 | 0.173 | 0.257 |  | 0.207 | 0.444 |  | 0.032 | 0.043 |  | 0.271 | 0.314 |
| Omy_ada10-71 | 0.340 | 0.599 |  | 0.209 | 0.427 |  | 0.087 | 0.039 |  | 0.423 | 0.325 |
| Omy_aromat-280 | 0.265 | 0.324 |  | 0.193 | 0.549 |  | 0.036 | 0.040 |  | 0.382 | 0.292 |
| Omy_arp-630 | 0.498 | 0.495 |  | 0.435 | 0.694 |  | 0.059 | 0.053 |  | 0.484 | 0.472 |
| Omy_aspAT-123 | 0.102 | 0.252 |  | 0.273 | 0.681 |  | 0.040 | 0.051 |  | 0.178 | 0.385 |
| Omy_b1-266 | 0.466 | 0.595 |  | 0.286 | 0.363 |  | 0.081 | 0.030 |  | 0.452 | 0.403 |
| Omy_BAC-B4-324 | 0.064 | 0.192 |  | 0.460 | 0.509 |  | 0.052 | 0.046 |  | 0.115 | 0.476 |
| Omy_BAC-F5.284 | 0.103 | 0.324 |  | 0.067 | 0.245 |  | 0.049 | 0.047 |  | 0.180 | 0.099 |
| Omy_BAMBI2.312 | 0.102 | 0.280 |  | 0.119 | 0.632 |  | 0.075 | 0.072 |  | 0.166 | 0.185 |
| Omy_ca050-64 | 0.153 | 0.212 |  | 0.344 | 0.428 |  | 0.027 | 0.034 |  | 0.254 | 0.430 |
| Omy_carban1-264 | 0.004 | 0.058 |  | 0.108 | 0.349 |  | 0.040 | 0.058 |  | 0.006 | 0.172 |
| Omy_cd28-130 | 0.363 | 0.445 |  | 0.045 | 0.308 |  | 0.055 | 0.102 |  | 0.444 | 0.056 |
| Omy_cd59b-112 | 0.147 | 0.224 |  | 0.131 | 0.387 |  | 0.036 | 0.052 |  | 0.238 | 0.202 |
| Omy_cin-172 | 0.233 | 0.301 |  | 0.212 | 0.421 |  | 0.044 | 0.044 |  | 0.342 | 0.325 |
| Omy_colla1-525 | 0.203 | 0.377 |  | 0.305 | 0.412 |  | 0.060 | 0.029 |  | 0.295 | 0.428 |
| Omy_cox2-335 | 0.090 | 0.254 |  | 0.164 | 0.354 |  | 0.044 | 0.042 |  | 0.147 | 0.274 |
| Omy_g1-103 | 0.024 | 0.222 |  | 0.082 | 0.245 |  | 0.079 | 0.051 |  | 0.038 | 0.126 |
| Omy_g12-82 | 0.443 | 0.427 |  | 0.468 | 0.645 |  | 0.046 | 0.054 |  | 0.502 | 0.479 |
| Omy_G3PD_2-371 | 0.163 | 0.281 |  | 0.178 | 0.483 |  | 0.038 | 0.038 |  | 0.243 | 0.276 |
| Omy_gadd45-332 | 0.002 | 0.023 |  | 0.107 | 0.467 |  | 0.014 | 0.084 |  | 0.004 | 0.167 |
| Omy_gh-475 | 0.087 | 0.273 |  | 0.123 | 0.273 |  | 0.065 | 0.037 |  | 0.127 | 0.210 |
| Omy_gluR-79 | 0.488 | 0.347 |  | 0.482 | 0.599 |  | 0.040 | 0.044 |  | 0.479 | 0.485 |
| Omy_hsf1b-241 | 0.195 | 0.410 |  | 0.099 | 0.487 |  | 0.044 | 0.058 |  | 0.299 | 0.163 |
| Omy_hsp70aPro-329 | 0.098 | 0.250 |  | 0.064 | 0.443 |  | 0.066 | 0.076 |  | 0.171 | 0.097 |
| Omy_hus1-52 | 0.370 | 0.411 |  | 0.098 | 0.290 |  | 0.049 | 0.069 |  | 0.353 | 0.135 |
| Omy_IL17-185 | 0.369 | 0.495 |  | 0.462 | 0.645 |  | 0.081 | 0.044 |  | 0.478 | 0.513 |
| Omy_Il-1b_.028 | 0.378 | 0.458 |  | 0.184 | 0.389 |  | 0.061 | 0.044 |  | 0.443 | 0.289 |
| Omy_impa1-55 | 0.001 | 0.011 |  | 0.093 | 0.305 |  | 0.010 | 0.049 |  | 0.002 | 0.148 |
| Omy_inos-97 | 0.030 | 0.130 |  | 0.064 | 0.267 |  | 0.059 | 0.054 |  | 0.057 | 0.097 |
| Omy_LDHB-1_i2 | 0.067 | 0.400 |  | 0.092 | 0.193 |  | 0.098 | 0.030 |  | 0.110 | 0.146 |
| Omy_LDHB-2_e5 | 0.332 | 0.400 |  | 0.169 | 0.478 |  | 0.031 | 0.045 |  | 0.459 | 0.271 |
| Omy_lpl-220 | 0.198 | 0.359 |  | 0.163 | 0.370 |  | 0.044 | 0.030 |  | 0.303 | 0.273 |
| Omy_mcsf-268 | 0.121 | 0.188 |  | 0.045 | 0.224 |  | 0.026 | 0.060 |  | 0.211 | 0.051 |
| Omy_myoD-178 | 0.045 | 0.101 |  | 0.122 | 0.353 |  | 0.016 | 0.050 |  | 0.084 | 0.199 |
| Omy_nach-200 | 0.032 | 0.263 |  | 0.024 | 0.124 |  | 0.091 | 0.030 |  | 0.052 | 0.027 |
| Omy_NaKATPa3-50 | 0.234 | 0.255 |  | 0.312 | 0.480 |  | 0.022 | 0.046 |  | 0.349 | 0.414 |
| Omy_nips-299 | 0.051 | 0.233 |  | 0.064 | 0.213 |  | 0.054 | 0.036 |  | 0.094 | 0.106 |
| Omy_Ots249-227 | 0.379 | 0.642 |  | 0.313 | 0.561 |  | 0.058 | 0.038 |  | 0.435 | 0.417 |
| Omy_oxct-85 | 0.273 | 0.456 |  | 0.115 | 0.329 |  | 0.053 | 0.045 |  | 0.383 | 0.187 |
| Omy_p53-262 | 0.067 | 0.308 |  | 0.190 | 0.495 |  | 0.057 | 0.053 |  | 0.125 | 0.298 |
| Omy_pad-196 | 0.001 | 0.029 |  | 0.049 | 0.199 |  | 0.027 | 0.037 |  | 0.002 | 0.067 |
| Omy_ppie-232 | 0.110 | 0.269 |  | 0.140 | 0.479 |  | 0.032 | 0.044 |  | 0.187 | 0.223 |
| Omy_rbm4b-203 | 0.425 | 0.505 |  | 0.196 | 0.489 |  | 0.078 | 0.050 |  | 0.448 | 0.301 |
| Omy_redd1-410 | 0.222 | 0.247 |  | 0.210 | 0.525 |  | 0.023 | 0.038 |  | 0.335 | 0.325 |
| Omy_sast-264 | 0.389 | 0.687 |  | 0.178 | 0.403 |  | 0.090 | 0.030 |  | 0.426 | 0.289 |
| Omy_srp09-37 | 0.134 | 0.206 |  | 0.321 | 0.476 |  | 0.031 | 0.043 |  | 0.236 | 0.421 |
| Omy_sSOD-1 | 0.318 | 0.481 |  | 0.044 | 0.330 |  | 0.044 | 0.098 |  | 0.434 | 0.050 |
| Omy_sys1-188 | 0.077 | 0.207 |  | 0.113 | 0.385 |  | 0.039 | 0.063 |  | 0.141 | 0.174 |
| Omy_tlr3-377 | 0.120 | 0.290 |  | 0.102 | 0.289 |  | 0.037 | 0.046 |  | 0.196 | 0.168 |
| Omy_txnip-343 | 0.491 | 0.609 |  | 0.238 | 0.523 |  | 0.065 | 0.053 |  | 0.489 | 0.343 |
| Omy_u07-79-166 | 0.480 | 0.353 |  | 0.119 | 0.391 |  | 0.038 | 0.070 |  | 0.484 | 0.184 |
| Omy_u09-54-311 | 0.363 | 0.309 |  | 0.274 | 0.561 |  | 0.025 | 0.065 |  | 0.477 | 0.367 |
| Omy_U11_2b-154 | 0.321 | 0.555 |  | 0.247 | 0.512 |  | 0.073 | 0.046 |  | 0.408 | 0.356 |
| Omy_vamp5-303 | 0.428 | 0.572 |  | 0.255 | 0.606 |  | 0.054 | 0.085 |  | 0.405 | 0.342 |
| Omy_zg57-91 | 0.020 | 0.096 |  | 0.113 | 0.418 |  | 0.030 | 0.054 |  | 0.039 | 0.184 |
| OMS00087 | omitted | omitted |  | omitted | omitted |  | --- | --- |  | --- | --- |
| OMS00169 | omitted | omitted |  | omitted | omitted |  | --- | --- |  | --- | --- |
| OMS00176 | omitted | omitted |  | omitted | omitted |  | --- | --- |  | --- | --- |
| Omy_crb-106 | omitted | omitted |  | omitted | omitted |  | --- | --- |  | --- | --- |
| Omy_Il1b-198 | omitted | omitted |  | omitted | omitted |  | --- | --- |  | --- | --- |
| Omy_mapK3-103 | omitted | omitted |  | omitted | omitted |  | --- | --- |  | --- | --- |
| Omy_rapd-167 | omitted | omitted |  | omitted | omitted |  | --- | --- |  | --- | --- |
| Omy_u09-52.284 | omitted | omitted |  | omitted | omitted |  | --- | --- |  | --- | --- |
| Ocl_gshpx-357 | N/A | N/A |  | N/A | N/A |  | --- | --- |  | --- | --- |
| Omy_myclarp404-111 | N/A | N/A |  | N/A | N/A |  | --- | --- |  | --- | --- |
| Omy_Omyclmk438-96 | N/A | N/A |  | N/A | N/A |  | --- | --- |  | --- | --- |
|  |  |  |  |  |  |  |  |  |  |  |  |
|  |  |  |  |  |  |  |  |  |  |  |  |

(a) Abadía-Cardoso et al. 2011, (b) Aguilar and Garza 2008, (c) Brunelli et al. 2008, (d) Campbell and Narum 2009, (e) Campbell et al. 2009, (f) Hansen et al. 2011, (g) Narum et al. 2010a, (h) Sanchez et al. 2009, (i) Sprowles et al. 2006,

(*JD) J. Dekoning, unpublished: dekoning@comcast.net

(*NC) N. Campbell, unpublished: camn@critfc.org

(*SY) S. Young, unpublished: youngsfy@dfw.wa.gov

Table S3. List of 191 SNP markers assayed for *O. mykiss* in the Columbia River Basin. Final classification of SNPs is: candidate (C), ambiguous (A), and neutral (N). Candidate loci and corresponding environmental associations are: P – precipitation, T – temperature, E – elevation, D – distance. Distance refers to both lat/long coordinates and migration in river kilometers. Notes include hybrid detection markers, HWE deviations, numbered pairs of linked SNPs (link), inland outlier loci “Oi”, coastal outlier loci “Oc”, and “precedence” loci numbered in parentheses. Precedence references are : 1. thermal stress association (Narum et al. 2013); 2. temperature and precipitation association (Narum et al. 2010a); 3. anadromy association (Narum et al. 2011). SNP discovery and primer sequence sources are identified by letter and are listed at bottom.

|  |  |  |  |  |  |  |  |
| --- | --- | --- | --- | --- | --- | --- | --- |
|  |  | inland |  | coastal |  |  |  |
| SNP locus | source | classification | association | classification | association |  | notes |
|  |  |  |  |  |  |  |  |
| Omy_97954-618 | a | **C** | D/P | A | --- |  | Oc |
| Omy_gdh-271 | e | **C** | E | A | --- |  | (2) |
| OMS00014 | h | **C** | P | N | --- |  | --- |
| OMS00062 | h | **C** | P | N | --- |  | --- |
| OMS00151 | h | **C** | T/P | N | --- |  | --- |
| Omy_97660-230 | a | **C** | T/D | N | --- |  | --- |
| Omy_CRBF1-1 | b | **C** | P/D | N | --- |  | link3 |
| Omy_e1-147 | i | **C** | D/T | N | --- |  | --- |
| Omy_GHSR-121 | *NC | **C** | P | N | --- |  | link2 |
| Omy_hsc715-80 | d | **C** | P | N | --- |  | --- |
| Omy_IL6-320 | *JD | **C** | T/P | N | --- |  | --- |
| Omy_metA-161 | *JD | **C** | T/P | N | --- |  | --- |
| Omy_nkef-241 | e | **C** | D | N | --- |  | --- |
| Omy_ntl-27 | e | **C** | D | N | --- |  | --- |
| Omy_SECC22b-88 | *NC | **C** | P | N | --- |  | link5 |
| Omy_u09-53.469 | *SY | **C** | T/P/D | N | --- |  | Oi |
| Omy_UT16_2-173 | *SY | **C** | D | N | --- |  | Oi |
| OMY1011SNP | f | **C** | P | N | --- |  | --- |
| Omy_hsp47-86 | d | **C** | T/D | **A** | --- |  | (1) |
| Omy_OmyP9-180 | e | **C** | T/P | **C** | T |  | (1) |
| Omy_stat3-273 | *JD | **C** | P | **C** | P |  | (2) |
| Omy_tlr5-205 | *JD | **C** | T/P/D | **A** | --- |  | (2) |
| Omy_aldB-165 | e | A | --- | **C** | T |  | (2) |
| Omy_hsf2-146 | d | A | --- | **C** | P/D |  | (2) |
| OMS00008 | h | N | --- | **C** | P |  | --- |
| OMS00058 | h | N | --- | **C** | T/D |  | --- |
| OMS00111 | h | N | --- | **C** | P/D |  | --- |
| Omy_bcAKala-380rd | g | N | --- | **C** | T/D |  | Oc |
| Omy_cox1-221 | e | N | --- | **C** | D |  | --- |
| Omy_anp-17 | g | A | --- | N | --- |  | Oi |
| Omy_b9-164 | i | A | --- | N | --- |  | Oi |
| Omy_IL1b-163 | *JD | A | --- | N | --- |  | Oi |
| Omy_nxt2-273 | e | A | --- | N | --- |  | Oi |
| Omy_star-206 | *JD | A | --- | N | --- |  | Oi |
| Omy_u09-56.119 | *SY | A | --- | N | --- |  | Oi |
| Omy_vatf-406 | g | A | --- | N | --- |  | Oi |
| Omy_LDHB-2_i6 | b | A | --- | A | --- |  | (3) |
| Omy_ndk-152 | *NC | A | --- | A | --- |  | link6; Oi; (3) |
| Omy_Ogo4-212 | *NC | A | --- | A | --- |  | (2) |
| OMS00064 | h | N | --- | A | --- |  | Oc |
| OMS00096 | h | N | --- | A | --- |  | Oc |
| OMS00118 | h | N | --- | A | --- |  | Oc |
| OMS00174 | h | N | --- | A | --- |  | Oc |
| Omy_cd59-206 | *JD | N | --- | A | --- |  | Oc |
| Omy_metB-138 | *JD | N | --- | A | --- |  | Oc |
| M09AAC.055 | *SY | N | --- | N | --- |  | --- |
| M09AAD.076 | *SY | N | --- | N | --- |  | --- |
| M09AAE.082 | *SY | N | --- | N | --- |  | --- |
| M09AAJ.163 | *SY | N | --- | N | --- |  | --- |
| OMGH1PROM1-SNP1 | a | N | --- | N | --- |  | --- |
| OMS00002 | h | N | --- | N | --- |  | --- |
| OMS00003 | h | N | --- | N | --- |  | --- |
| OMS00006 | h | N | --- | N | --- |  | --- |
| OMS00013 | h | N | --- | N | --- |  | --- |
| OMS00015 | h | N | --- | N | --- |  | --- |
| OMS00017 | h | N | --- | N | --- |  | --- |
| OMS00018 | h | N | --- | N | --- |  | --- |
| OMS00024 | h | N | --- | N | --- |  | --- |
| OMS00030 | h | N | --- | N | --- |  | --- |
| OMS00039 | h | N | --- | N | --- |  | --- |
| OMS00048 | h | N | --- | N | --- |  | --- |
| OMS00052 | h | N | --- | N | --- |  | --- |
| OMS00053 | h | N | --- | N | --- |  | --- |
| OMS00056 | h | N | --- | N | --- |  | --- |
| OMS00057 | h | N | --- | N | --- |  | --- |
| OMS00061 | h | N | --- | N | --- |  | --- |
| OMS00068 | h | N | --- | N | --- |  | --- |
| OMS00070 | h | N | --- | N | --- |  | --- |
| OMS00071 | h | N | --- | N | --- |  | --- |
| OMS00072 | h | N | --- | N | --- |  | --- |
| OMS00074 | h | N | --- | N | --- |  | --- |
| OMS00077 | h | N | --- | N | --- |  | --- |
| OMS00078 | h | N | --- | N | --- |  | --- |
| OMS00079 | h | N | --- | N | --- |  | --- |
| OMS00089 | h | N | --- | N | --- |  | --- |
| OMS00090 | h | N | --- | N | --- |  | --- |
| OMS00092 | h | N | --- | N | --- |  | --- |
| OMS00095 | h | N | --- | N | --- |  | --- |
| OMS00101 | h | N | --- | N | --- |  | --- |
| OMS00105 | h | N | --- | N | --- |  | --- |
| OMS00106 | h | N | --- | N | --- |  | --- |
| OMS00112 | h | N | --- | N | --- |  | --- |
| OMS00114 | h | N | --- | N | --- |  | --- |
| OMS00119 | h | N | --- | N | --- |  | --- |
| OMS00120 | h | N | --- | N | --- |  | --- |
| OMS00121 | h | N | --- | N | --- |  | --- |
| OMS00129 | h | N | --- | N | --- |  | --- |
| OMS00132 | h | N | --- | N | --- |  | --- |
| OMS00133 | h | N | --- | N | --- |  | link1 |
| OMS00138 | h | N | --- | N | --- |  | --- |
| OMS00143 | h | N | --- | N | --- |  | --- |
| OMS00149 | h | N | --- | N | --- |  | --- |
| OMS00154 | h | N | --- | N | --- |  | --- |
| OMS00173 | h | N | --- | N | --- |  | --- |
| OMS00175 | h | N | --- | N | --- |  | --- |
| OMS00179 | h | N | --- | N | --- |  | --- |
| OMS00180 | h | N | --- | N | --- |  | --- |
| Omy_101832-195 | a | N | --- | N | --- |  | --- |
| Omy_101993-189 | a | N | --- | N | --- |  | --- |
| Omy_102505-102 | a | N | --- | N | --- |  | --- |
| Omy_103705-558 | a | N | --- | N | --- |  | --- |
| Omy_104519-624 | a | N | --- | N | --- |  | --- |
| Omy_105075-162 | a | N | --- | N | --- |  | --- |
| Omy_105105-448 | a | N | --- | N | --- |  | --- |
| Omy_105385-406 | a | N | --- | N | --- |  | --- |
| Omy_105714-265 | a | N | --- | N | --- |  | --- |
| Omy_107031-704 | a | N | --- | N | --- |  | --- |
| Omy_107285-69 | a | N | --- | N | --- |  | --- |
| Omy_107806-34 | a | N | --- | N | --- |  | --- |
| Omy_108007-193 | a | N | --- | N | --- |  | --- |
| Omy_109243-222 | a | N | --- | N | --- |  | --- |
| Omy_109894-185 | a | N | --- | N | --- |  | --- |
| Omy_110064-419 | a | N | --- | N | --- |  | --- |
| Omy_110201-359 | a | N | --- | N | --- |  | --- |
| Omy_111383-51 | a | N | --- | N | --- |  | --- |
| Omy_113490-159 | a | N | --- | N | --- |  | --- |
| Omy_114315-438 | a | N | --- | N | --- |  | --- |
| Omy_114587-480 | a | N | --- | N | --- |  | --- |
| Omy_116733-349 | a | N | --- | N | --- |  | --- |
| Omy_128923-433 | a | N | --- | N | --- |  | --- |
| Omy_128996-481 | a | N | --- | N | --- |  | --- |
| Omy_129870-756 | a | N | --- | N | --- |  | --- |
| Omy_130524-160 | a | N | --- | N | --- |  | --- |
| Omy_97077-73 | a | N | --- | N | --- |  | --- |
| Omy_97865-196 | a | N | --- | N | --- |  | --- |
| Omy_99300-202 | a | N | --- | N | --- |  | --- |
| Omy_ada10-71 | g | N | --- | N | --- |  | --- |
| Omy_aromat-280 | *JD | N | --- | N | --- |  | --- |
| Omy_arp-630 | e | N | --- | N | --- |  | --- |
| Omy_aspAT-123 | e | N | --- | N | --- |  | --- |
| Omy_b1-266 | i | N | --- | N | --- |  | --- |
| Omy_BAC-B4-324 | *SY | N | --- | N | --- |  | --- |
| Omy_BAC-F5.284 | i | N | --- | N | --- |  | --- |
| Omy_BAMBI2.312 | *SY | N | --- | N | --- |  | --- |
| Omy_ca050-64 | g | N | --- | N | --- |  | --- |
| Omy_carban1-264 | g | N | --- | N | --- |  | --- |
| Omy_cd28-130 | *JD | N | --- | N | --- |  | --- |
| Omy_cd59b-112 | *JD | N | --- | N | --- |  | --- |
| Omy_cin-172 | g | N | --- | N | --- |  | --- |
| Omy_colla1-525 | *JD | N | --- | N | --- |  | --- |
| Omy_cox2-335 | *JD | N | --- | N | --- |  | --- |
| Omy_g1-103 | i | N | --- | N | --- |  | --- |
| Omy_g12-82 | *JD | N | --- | N | --- |  | --- |
| Omy_G3PD_2-371 | *SY | N | --- | N | --- |  | --- |
| Omy_gadd45-332 | *NC | N | --- | N | --- |  | --- |
| Omy_gh-475 | *NC | N | --- | N | --- |  | --- |
| Omy_gluR-79 | *NC | N | --- | N | --- |  | --- |
| Omy_hsf1b-241 | d | N | --- | N | --- |  | --- |
| Omy_hsp70aPro-329 | d | N | --- | N | --- |  | --- |
| Omy_hus1-52 | g | N | --- | N | --- |  | --- |
| Omy_IL17-185 | *JD | N | --- | N | --- |  | --- |
| Omy_Il-1b_.028 | *SY | N | --- | N | --- |  | link4 |
| Omy_impa1-55 | g | N | --- | N | --- |  | --- |
| Omy_inos-97 | *JD | N | --- | N | --- |  | --- |
| Omy_LDHB-1_i2 | b | N | --- | N | --- |  | --- |
| Omy_LDHB-2_e5 | b | N | --- | N | --- |  | --- |
| Omy_lpl-220 | g | N | --- | N | --- |  | --- |
| Omy_mcsf-268 | *JD | N | --- | N | --- |  | --- |
| Omy_myoD-178 | e | N | --- | N | --- |  | --- |
| Omy_nach-200 | *JD | N | --- | N | --- |  | --- |
| Omy_NaKATPa3-50 | e | N | --- | N | --- |  | --- |
| Omy_nips-299 | e | N | --- | N | --- |  | --- |
| Omy_Ots249-227 | e | N | --- | N | --- |  | --- |
| Omy_oxct-85 | *JD | N | --- | N | --- |  | --- |
| Omy_p53-262 | *NC | N | --- | N | --- |  | --- |
| Omy_pad-196 | *NC | N | --- | N | --- |  | --- |
| Omy_ppie-232 | g | N | --- | N | --- |  | --- |
| Omy_rbm4b-203 | g | N | --- | N | --- |  | --- |
| Omy_redd1-410 | g | N | --- | N | --- |  | --- |
| Omy_sast-264 | g | N | --- | N | --- |  | --- |
| Omy_srp09-37 | g | N | --- | N | --- |  | --- |
| Omy_sSOD-1 | c | N | --- | N | --- |  | --- |
| Omy_sys1-188 | g | N | --- | N | --- |  | --- |
| Omy_tlr3-377 | *JD | N | --- | N | --- |  | --- |
| Omy_txnip-343 | g | N | --- | N | --- |  | --- |
| Omy_u07-79-166 | *SY | N | --- | N | --- |  | --- |
| Omy_u09-54-311 | *SY | N | --- | N | --- |  | --- |
| Omy_U11_2b-154 | *SY | N | --- | N | --- |  | --- |
| Omy_vamp5-303 | g | N | --- | N | --- |  | --- |
| Omy_zg57-91 | g | N | --- | N | --- |  | --- |
| OMS00087 | h | omitted | --- | omitted | --- |  | HWE |
| OMS00169 | h | omitted | --- | omitted | --- |  | link5 |
| OMS00176 | h | omitted | --- | omitted | --- |  | link2 |
| Omy_crb-106 | i | omitted | --- | omitted | --- |  | link3 |
| Omy_Il1b-198 | *SY | omitted | --- | omitted | --- |  | link4 |
| Omy_mapK3-103 | *JD | omitted | --- | omitted | --- |  | link2 |
| Omy_rapd-167 | i | omitted | --- | omitted | --- |  | link1 |
| Omy_u09-52.284 | *SY | omitted | --- | omitted | --- |  | link6 |
| Ocl_gshpx-357 | e | N/A | --- | N/A | --- |  | hybrid |
| Omy_myclarp404-111 | *NC | N/A | --- | N/A | --- |  | hybrid |
| Omy_Omyclmk438-96 | i | N/A | --- | N/A | --- |  | hybrid |
|  |  |  |  |  |  |  |  |

(a) Abadía-Cardoso et al. 2011, (b) Aguilar and Garza 2008, (c) Brunelli et al. 2008, (d) Campbell and Narum 2009, (e) Campbell et al. 2009, (f) Hansen et al. 2011, (g) Narum et al. 2010a, (h) Sanchez et al. 2009, (i) Sprowles et al. 2006,

(*JD) J. Dekoning, unpublished: dekoning@comcast.net

(*NC) N. Campbell, unpublished: camn@critfc.org

(*SY) S. Young, unpublished: youngsfy@dfw.wa.gov

Table S4. Values for predictor variables (by population) to control for underlying population structure in association tests (i.e., neutral variation). Refer to Table 1 for population descriptions according to reference number (Ref.#). Variables are: latitude and longitude, the Eigen vectors from PCA (EV1-EV3), and membership coefficients from Bayesian cluster analysis conducted in STRUCTURE version 2.3.4 (Pritchard et al. 2000). The cluster analyses are based on 172 and 170 loci (excluding F_st_ outliers) for coastal and inland lineages respectively. Highest (Q) membership in k=6 cluster are in bold italics for each population. Snake River reference hatchery populations 140-145 were not evaluated in landscape analysis (not listed).

|  |  |  |  |  |  |  |  |  |  |  |  |  |  |
| --- | --- | --- | --- | --- | --- | --- | --- | --- | --- | --- | --- | --- | --- |
|  |  |  |  | PCA | | |  | STRUCTURE | | | | | |
| Ref. # | lat | Long |  | EV1 | EV2 | EV3 |  | (Q1) | (Q2) | (Q3) | (Q4) | (Q5) | (Q6) |
| 1 | 47.3582 | -123.9940 |  | 0.0379 | 0.0027 | -0.0169 |  | ***0.8880*** | 0.0280 | 0.0270 | 0.0170 | 0.0220 | 0.0170 |
| 2 | 45.2417 | -122.2817 |  | -0.0511 | 0.0071 | -0.0019 |  | 0.0490 | 0.0640 | 0.0670 | ***0.6590*** | 0.1220 | 0.0400 |
| 3 | 45.3514 | -122.3840 |  | -0.0292 | 0.0098 | -0.0065 |  | 0.1000 | 0.1730 | 0.1710 | ***0.4350*** | 0.0750 | 0.0470 |
| 4 | 45.3254 | -122.2885 |  | -0.0268 | 0.0059 | -0.0085 |  | 0.1360 | 0.1470 | 0.1200 | ***0.4560*** | 0.1040 | 0.0370 |
| 5 | 45.2417 | -122.2817 |  | 0.0094 | -0.0347 | 0.0098 |  | 0.0240 | 0.0440 | 0.0250 | 0.0230 | ***0.8560*** | 0.0280 |
| 6 | 44.7508 | -122.3967 |  | -0.0506 | -0.0124 | 0.0124 |  | 0.0420 | 0.0480 | 0.0550 | ***0.5090*** | 0.2520 | 0.0940 |
| 7 | 44.7970 | -122.7730 |  | -0.0796 | 0.0082 | -0.0044 |  | 0.0270 | 0.0410 | 0.0360 | ***0.8400*** | 0.0310 | 0.0240 |
| 8 | 44.4136 | -122.6772 |  | -0.0696 | 0.0100 | -0.0055 |  | 0.0260 | 0.0400 | 0.0330 | ***0.7410*** | 0.1380 | 0.0220 |
| 9 | 44.9077 | -123.4194 |  | 0.0374 | 0.0717 | 0.0505 |  | 0.0640 | ***0.7400*** | 0.1010 | 0.0260 | 0.0510 | 0.0190 |
| 10 | 44.7474 | -123.1477 |  | 0.0186 | 0.0617 | -0.0174 |  | 0.0440 | ***0.7100*** | 0.0760 | 0.0750 | 0.0810 | 0.0130 |
| 11 | 45.0784 | -123.4777 |  | -0.0099 | 0.0202 | -0.0240 |  | 0.0550 | ***0.2930*** | 0.1810 | 0.2160 | 0.2250 | 0.0300 |
| 12 | 46.2256 | -123.1481 |  | 0.0253 | -0.0007 | -0.0090 |  | 0.0920 | ***0.5170*** | 0.2050 | 0.0440 | 0.1070 | 0.0350 |
| 13 | 46.1408 | -122.8536 |  | 0.0300 | -0.0063 | -0.0135 |  | 0.1140 | 0.1740 | ***0.4850*** | 0.0280 | 0.1560 | 0.0430 |
| 14 | 46.5026 | -122.5881 |  | 0.0333 | -0.0200 | -0.0124 |  | 0.0620 | 0.0670 | ***0.7740*** | 0.0180 | 0.0530 | 0.0260 |
| 15 | 46.1910 | -123.1240 |  | 0.0288 | 0.0039 | -0.0029 |  | 0.1040 | ***0.4030*** | 0.3190 | 0.0380 | 0.0940 | 0.0430 |
| 16 | 46.0449 | -122.8039 |  | 0.0150 | -0.0170 | -0.0021 |  | 0.0530 | 0.1520 | 0.1630 | 0.0430 | ***0.5470*** | 0.0410 |
| 17 | 46.0449 | -122.8039 |  | 0.0215 | -0.0129 | -0.0113 |  | 0.0690 | 0.1970 | ***0.4600*** | 0.0460 | 0.1700 | 0.0590 |
| 18 | 45.8655 | -122.7184 |  | 0.0057 | -0.0105 | -0.0136 |  | 0.0660 | 0.1980 | ***0.3300*** | 0.0930 | 0.2740 | 0.0390 |
| 19 | 45.9516 | -122.5654 |  | 0.0165 | -0.0123 | -0.0132 |  | 0.0850 | 0.1140 | ***0.6020*** | 0.0500 | 0.0860 | 0.0630 |
| 20 | 46.1901 | -123.1758 |  | 0.0171 | -0.0014 | -0.0061 |  | 0.1380 | 0.2290 | ***0.3430*** | 0.0670 | 0.1740 | 0.0500 |
| 21 | 45.3309 | -121.9158 |  | 0.0210 | -0.0133 | -0.0013 |  | 0.0880 | 0.1340 | ***0.3390*** | 0.0510 | 0.3350 | 0.0520 |
| 22 | 45.5745 | -121.6271 |  | -0.0048 | -0.0134 | 0.0124 |  | 0.0630 | 0.1420 | 0.2350 | 0.1410 | 0.1560 | ***0.2630*** |
| 23 | 45.6047 | -121.6335 |  | 0.0042 | -0.0230 | 0.0110 |  | 0.0900 | 0.0950 | 0.0750 | 0.0750 | ***0.5450*** | 0.1200 |
| 24 | 45.7993 | -121.4846 |  | -0.0003 | -0.0232 | 0.0743 |  | 0.0300 | 0.0370 | 0.0460 | 0.0390 | 0.0390 | ***0.8090*** |
|  |  |  |  |  |  |  |  |  |  |  |  |  |  |
| 25 | 45.8452 | -121.0421 |  | 0.0108 | 0.0459 | 0.0185 |  | 0.0380 | ***0.7816*** | 0.0426 | 0.0600 | 0.0440 | 0.0338 |
| 26 | 45.9420 | -121.1439 |  | 0.0099 | 0.0428 | 0.0164 |  | 0.0394 | ***0.7073*** | 0.0446 | 0.0714 | 0.0818 | 0.0556 |
| 27 | 45.9876 | -121.1255 |  | 0.0065 | 0.0353 | 0.0158 |  | 0.0681 | ***0.7022*** | 0.0476 | 0.0785 | 0.0542 | 0.0494 |
| 28 | 46.0378 | -121.1994 |  | 0.0085 | 0.0351 | 0.0163 |  | 0.0690 | ***0.6336*** | 0.0448 | 0.0904 | 0.1044 | 0.0578 |
| 29 | 46.0133 | -121.1500 |  | 0.0101 | 0.0381 | 0.0179 |  | 0.0580 | ***0.7292*** | 0.0502 | 0.0648 | 0.0546 | 0.0432 |
| 30 | 45.8281 | -121.1724 |  | 0.0023 | 0.0332 | 0.0140 |  | 0.0478 | ***0.6220*** | 0.0566 | 0.1666 | 0.0596 | 0.0474 |
| 31 | 46.1957 | -121.2557 |  | 0.0094 | 0.0383 | 0.0241 |  | 0.0748 | ***0.6724*** | 0.0530 | 0.0666 | 0.0712 | 0.0620 |
| 32 | 45.8091 | -121.0652 |  | 0.0090 | 0.0498 | 0.0210 |  | 0.0228 | ***0.8341*** | 0.0288 | 0.0616 | 0.0320 | 0.0206 |
| 33 | 46.0774 | -121.2122 |  | -0.0129 | 0.0243 | 0.0513 |  | ***0.4034*** | 0.3286 | 0.1194 | 0.0788 | 0.0412 | 0.0286 |
| 34 | 45.8434 | -121.0605 |  | 0.0078 | 0.0449 | 0.0141 |  | 0.0434 | ***0.7552*** | 0.0426 | 0.0664 | 0.0378 | 0.0546 |
| 35 | 45.2507 | -121.0230 |  | 0.0057 | -0.0004 | -0.0050 |  | 0.1144 | 0.1140 | 0.0956 | 0.1256 | 0.2255 | ***0.3247*** |
| 36 | 44.7260 | -121.2478 |  | 0.0136 | 0.0138 | -0.0011 |  | 0.0580 | ***0.4091*** | 0.0626 | 0.1850 | 0.0819 | 0.2033 |
| 37 | 44.6942 | -121.2312 |  | 0.0090 | 0.0009 | -0.0042 |  | 0.1533 | 0.1751 | 0.0576 | 0.1486 | 0.1929 | ***0.2725*** |
| 38 | 44.7615 | -121.2288 |  | 0.0103 | -0.0022 | -0.0047 |  | 0.1778 | 0.1412 | 0.0582 | 0.0994 | 0.1948 | ***0.3287*** |
| 39 | 44.8217 | -121.0858 |  | 0.0062 | 0.0006 | -0.0082 |  | 0.0926 | 0.1156 | 0.0828 | 0.1348 | 0.2386 | ***0.3356*** |
| 40 | 45.6251 | -121.0656 |  | 0.0133 | 0.0182 | 0.0002 |  | 0.0678 | ***0.4592*** | 0.0440 | 0.1268 | 0.1124 | 0.1897 |
| 41 | 44.3629 | -119.7700 |  | 0.0100 | -0.0083 | -0.0186 |  | 0.1066 | 0.0424 | 0.0634 | 0.1040 | 0.1431 | ***0.5406*** |
| 42 | 44.4733 | -119.0332 |  | 0.0146 | -0.0019 | -0.0179 |  | 0.0842 | 0.0538 | 0.0458 | 0.0939 | 0.1455 | ***0.5768*** |
| 43 | 44.7358 | -120.3071 |  | 0.0043 | -0.0041 | -0.0116 |  | 0.0766 | 0.0598 | 0.0866 | 0.1244 | 0.2558 | ***0.3967*** |
| 44 | 44.3945 | -118.5764 |  | 0.0147 | 0.0043 | -0.0169 |  | 0.0632 | 0.0706 | 0.0424 | 0.0704 | 0.0820 | ***0.6715*** |
| 45 | 44.6195 | -118.5679 |  | 0.0089 | -0.0027 | -0.0083 |  | 0.1496 | 0.0616 | 0.0680 | 0.0864 | 0.1332 | ***0.5012*** |
| 46 | 44.8833 | -119.4121 |  | 0.0149 | -0.0040 | -0.0081 |  | 0.1308 | 0.0598 | 0.0416 | 0.0802 | 0.2425 | ***0.4451*** |
| 47 | 44.8383 | -118.4770 |  | 0.0199 | -0.0112 | -0.0041 |  | 0.2927 | 0.0394 | 0.0598 | 0.0624 | 0.1226 | ***0.4231*** |
| 48 | 45.0213 | -118.9905 |  | 0.0145 | -0.0032 | -0.0131 |  | 0.1214 | 0.0438 | 0.0400 | 0.1140 | 0.1554 | ***0.5255*** |
| 49 | 44.1956 | -119.4716 |  | 0.0189 | 0.0030 | -0.0211 |  | 0.0646 | 0.0544 | 0.0372 | 0.0546 | 0.1070 | ***0.6821*** |
| 50 | 44.2646 | -119.2857 |  | 0.0124 | -0.0003 | -0.0176 |  | 0.0856 | 0.0554 | 0.0464 | 0.0710 | 0.1393 | ***0.6022*** |
| 51 | 45.8066 | -120.5104 |  | -0.0001 | -0.0013 | -0.0054 |  | 0.1064 | 0.1110 | 0.0822 | 0.1938 | 0.2312 | ***0.2755*** |
| 52 | 45.8191 | -120.4890 |  | 0.0012 | -0.0020 | -0.0031 |  | 0.1054 | 0.1148 | 0.0908 | 0.1937 | ***0.2563*** | 0.2389 |
| 53 | 45.6997 | -118.3971 |  | 0.0075 | -0.0020 | -0.0093 |  | 0.1064 | 0.0952 | 0.0622 | 0.1176 | 0.1802 | ***0.4385*** |
| 54 | 45.7240 | -118.1879 |  | 0.0059 | 0.0039 | -0.0157 |  | 0.0696 | 0.1084 | 0.0872 | 0.1351 | 0.1246 | ***0.4751*** |
| 55 | 46.0470 | -118.6770 |  | 0.0110 | -0.0054 | -0.0079 |  | 0.1794 | 0.0526 | 0.0484 | 0.1124 | 0.1058 | ***0.5015*** |
| 56 | 47.0897 | -121.2881 |  | 0.0200 | 0.0061 | -0.0257 |  | 0.0502 | 0.0706 | 0.0400 | 0.1236 | 0.0516 | ***0.6641*** |
| 57 | 46.8338 | -120.9451 |  | 0.0175 | 0.0094 | -0.0145 |  | 0.0678 | 0.1366 | 0.0370 | 0.1574 | 0.0826 | ***0.5186*** |
| 58 | 47.0448 | -121.1829 |  | 0.0214 | 0.0080 | -0.0197 |  | 0.0750 | 0.1036 | 0.0312 | 0.1304 | 0.0662 | ***0.5935*** |
| 59 | 47.0178 | -121.1338 |  | 0.0236 | 0.0014 | -0.0193 |  | 0.0508 | 0.0486 | 0.0338 | 0.1142 | 0.0844 | ***0.6682*** |
| 60 | 46.8201 | -120.9307 |  | 0.0200 | 0.0059 | -0.0208 |  | 0.0606 | 0.1181 | 0.0326 | 0.1527 | 0.0718 | ***0.5641*** |
| 61 | 46.2621 | -120.1124 |  | 0.0122 | 0.0014 | -0.0195 |  | 0.0720 | 0.0448 | 0.0550 | 0.1394 | 0.0960 | ***0.5928*** |
| 62 | 46.3240 | -120.1697 |  | 0.0232 | 0.0139 | -0.0174 |  | 0.0652 | 0.2062 | 0.0290 | 0.1498 | 0.0276 | ***0.5223*** |
| 63 | 47.6881 | -120.7407 |  | 0.0064 | 0.0083 | -0.0050 |  | 0.0990 | 0.2145 | 0.0816 | 0.1434 | 0.1330 | ***0.3285*** |
| 64 | 47.5591 | -120.6742 |  | 0.0001 | -0.0004 | -0.0034 |  | 0.1112 | 0.0980 | 0.1152 | 0.1534 | 0.2104 | ***0.3117*** |
| 65 | 47.5591 | -120.6742 |  | 0.0082 | -0.0044 | -0.0110 |  | 0.0684 | 0.0814 | 0.0500 | 0.1755 | ***0.3997*** | 0.2250 |
| 66 | 47.8018 | -120.7146 |  | 0.0050 | -0.0048 | -0.0137 |  | 0.1144 | 0.0706 | 0.0786 | 0.1434 | 0.0850 | ***0.5079*** |
| 67 | 47.4923 | -120.6378 |  | 0.0012 | 0.0055 | -0.0061 |  | 0.1136 | 0.1396 | 0.1154 | 0.1385 | 0.1548 | ***0.3381*** |
| 68 | 47.7107 | -120.8051 |  | 0.0008 | 0.0056 | -0.0007 |  | 0.1490 | 0.1680 | 0.1130 | 0.1552 | 0.1166 | ***0.2982*** |
| 69 | 47.6964 | -120.3227 |  | 0.0019 | 0.0020 | -0.0050 |  | 0.1182 | 0.1284 | 0.1080 | 0.1398 | 0.1618 | ***0.3437*** |
| 70 | 48.4756 | -120.1819 |  | 0.0038 | 0.0013 | -0.0035 |  | 0.1036 | 0.1429 | 0.0962 | 0.1158 | 0.2461 | ***0.2953*** |
| 71 | 48.6998 | -119.4399 |  | 0.0059 | 0.0021 | -0.0034 |  | 0.0878 | 0.1686 | 0.0694 | 0.1404 | ***0.3109*** | 0.2230 |
| 72 | 48.3957 | -119.5043 |  | 0.0052 | 0.0057 | -0.0049 |  | 0.0762 | 0.1644 | 0.0800 | 0.1243 | 0.2432 | ***0.3120*** |
| 73 | 48.3747 | -119.5911 |  | 0.0087 | 0.0211 | 0.0010 |  | 0.0374 | ***0.4434*** | 0.0542 | 0.1748 | 0.1347 | 0.1555 |
| 74 | 46.3097 | -117.6572 |  | 0.0058 | -0.0029 | -0.0076 |  | 0.1064 | 0.0798 | 0.0780 | 0.1366 | 0.1958 | ***0.4033*** |
| 75 | 46.4076 | -117.2198 |  | 0.0054 | -0.0026 | -0.0059 |  | 0.1022 | 0.1038 | 0.0762 | 0.1496 | 0.2340 | ***0.3343*** |
| 76 | 46.3228 | -117.1368 |  | 0.0054 | -0.0017 | -0.0058 |  | 0.1232 | 0.1195 | 0.0908 | 0.1292 | 0.2477 | ***0.2897*** |
| 77 | 46.3442 | -117.0551 |  | 0.0034 | -0.0031 | -0.0051 |  | 0.1080 | 0.1002 | 0.0882 | 0.1418 | ***0.3013*** | 0.2605 |
| 78 | 46.1515 | -116.9340 |  | 0.0011 | -0.0059 | -0.0037 |  | 0.1423 | 0.0706 | 0.0930 | 0.1411 | 0.2429 | ***0.3101*** |
| 79 | 46.3029 | -117.1168 |  | 0.0024 | -0.0018 | -0.0060 |  | 0.1120 | 0.0836 | 0.0944 | 0.1316 | ***0.2935*** | 0.2849 |
| 80 | 46.3672 | -116.7360 |  | 0.0056 | -0.0045 | -0.0066 |  | 0.1158 | 0.0538 | 0.0744 | 0.1423 | 0.1991 | ***0.4146*** |
| 81 | 46.6306 | -116.6562 |  | -0.0062 | 0.0007 | -0.0075 |  | 0.0652 | 0.0916 | 0.1214 | ***0.2800*** | 0.1822 | 0.2596 |
| 82 | 46.7984 | -116.4194 |  | -0.0123 | -0.0032 | -0.0052 |  | 0.0900 | 0.0726 | 0.1874 | ***0.3237*** | 0.1336 | 0.1926 |
| 83 | 46.6372 | -116.6780 |  | -0.0068 | -0.0024 | -0.0055 |  | 0.0820 | 0.0750 | 0.1336 | ***0.2766*** | 0.1784 | 0.2545 |
| 84 | 46.8054 | -116.4182 |  | -0.0093 | -0.0030 | -0.0080 |  | 0.0978 | 0.0572 | 0.1337 | ***0.3487*** | 0.1333 | 0.2293 |
| 85 | 46.0191 | -114.8378 |  | -0.0455 | 0.0021 | 0.0034 |  | 0.0294 | 0.0342 | ***0.8342*** | 0.0446 | 0.0240 | 0.0336 |
| 86 | 46.0583 | -115.3141 |  | -0.0423 | -0.0030 | -0.0026 |  | 0.0364 | 0.0240 | ***0.7268*** | 0.1373 | 0.0384 | 0.0370 |
| 87 | 45.7441 | -114.7895 |  | -0.0424 | -0.0045 | -0.0012 |  | 0.0342 | 0.0296 | ***0.7974*** | 0.0756 | 0.0298 | 0.0334 |
| 88 | 45.6921 | -114.7175 |  | -0.0455 | -0.0006 | -0.0008 |  | 0.0344 | 0.0222 | ***0.8192*** | 0.0612 | 0.0286 | 0.0344 |
| 89 | 46.1634 | -114.9006 |  | -0.0392 | -0.0042 | 0.0016 |  | 0.0556 | 0.0220 | ***0.7341*** | 0.1201 | 0.0330 | 0.0352 |
| 90 | 46.0809 | -115.5179 |  | -0.0358 | -0.0012 | -0.0019 |  | 0.0502 | 0.0350 | ***0.5055*** | 0.2981 | 0.0576 | 0.0536 |
| 91 | 46.0981 | -115.0728 |  | -0.0486 | -0.0069 | 0.0004 |  | 0.0348 | 0.0152 | ***0.8598*** | 0.0406 | 0.0244 | 0.0252 |
| 92 | 45.8689 | -114.7205 |  | -0.0449 | -0.0025 | -0.0011 |  | 0.0328 | 0.0230 | ***0.8408*** | 0.0446 | 0.0278 | 0.0310 |
| 93 | 46.2161 | -115.5559 |  | -0.0409 | 0.0033 | 0.0027 |  | 0.0364 | 0.0404 | ***0.6006*** | 0.2472 | 0.0408 | 0.0346 |
| 94 | 46.4311 | -114.5395 |  | -0.0482 | 0.0034 | -0.0025 |  | 0.0256 | 0.0206 | ***0.8466*** | 0.0570 | 0.0252 | 0.0250 |
| 95 | 46.5251 | -114.6786 |  | -0.0439 | -0.0017 | 0.0014 |  | 0.0302 | 0.0272 | ***0.7035*** | 0.1752 | 0.0352 | 0.0288 |
| 96 | 46.3336 | -115.3471 |  | -0.0450 | -0.0008 | 0.0022 |  | 0.0392 | 0.0218 | ***0.7868*** | 0.0844 | 0.0336 | 0.0342 |
| 97 | 46.4632 | -114.9965 |  | -0.0433 | -0.0008 | -0.0004 |  | 0.0396 | 0.0196 | ***0.8123*** | 0.0588 | 0.0272 | 0.0426 |
| 98 | 46.4607 | -114.5467 |  | -0.0469 | 0.0048 | -0.0006 |  | 0.0264 | 0.0176 | ***0.8371*** | 0.0664 | 0.0264 | 0.0260 |
| 99 | 46.0486 | -115.7814 |  | -0.0327 | 0.0055 | -0.0013 |  | 0.0510 | 0.0386 | 0.3080 | ***0.5149*** | 0.0406 | 0.0468 |
| 100 | 45.8211 | -115.5272 |  | -0.0340 | 0.0006 | -0.0027 |  | 0.0272 | 0.0190 | 0.2290 | ***0.6636*** | 0.0300 | 0.0312 |
| 101 | 45.8224 | -115.8887 |  | -0.0319 | 0.0026 | -0.0107 |  | 0.0302 | 0.0480 | 0.3042 | ***0.5062*** | 0.0444 | 0.0670 |
| 102 | 45.8057 | -115.6833 |  | -0.0365 | 0.0033 | -0.0081 |  | 0.0434 | 0.0372 | 0.3933 | ***0.4279*** | 0.0460 | 0.0522 |
| 103 | 45.6380 | -116.2828 |  | 0.0057 | -0.0081 | 0.0044 |  | 0.2358 | 0.0808 | 0.0800 | 0.1166 | ***0.3137*** | 0.1730 |
| 104 | 45.7523 | -116.3198 |  | 0.0009 | -0.0077 | 0.0036 |  | 0.1974 | 0.0646 | 0.0994 | 0.1332 | ***0.3569*** | 0.1484 |
| 105 | 45.9770 | -117.5550 |  | 0.0088 | -0.0003 | -0.0061 |  | 0.1245 | 0.1076 | 0.0566 | 0.1238 | 0.2067 | ***0.3808*** |
| 106 | 45.7053 | -117.1529 |  | -0.0009 | -0.0043 | -0.0163 |  | 0.0962 | 0.0330 | 0.1050 | 0.1608 | 0.1312 | ***0.4738*** |
| 107 | 46.0278 | -117.0177 |  | 0.0046 | -0.0008 | -0.0099 |  | 0.1338 | 0.0720 | 0.0902 | 0.1158 | 0.1762 | ***0.4119*** |
| 108 | 45.4004 | -117.6722 |  | 0.0139 | -0.0012 | -0.0187 |  | 0.0782 | 0.0674 | 0.0364 | 0.1060 | 0.1178 | ***0.5943*** |
| 109 | 45.5521 | -117.4898 |  | 0.0135 | 0.0067 | -0.0144 |  | 0.0642 | 0.1525 | 0.0384 | 0.1271 | 0.1705 | ***0.4472*** |
| 110 | 46.0075 | -117.3651 |  | 0.0082 | -0.0018 | -0.0040 |  | 0.0901 | 0.1151 | 0.0498 | 0.1273 | ***0.3570*** | 0.2607 |
| 111 | 45.9453 | -117.4513 |  | 0.0056 | -0.0029 | -0.0111 |  | 0.1156 | 0.0604 | 0.0688 | 0.1010 | 0.1870 | ***0.4673*** |
| 112 | 45.5574 | -116.8345 |  | 0.0078 | -0.0142 | -0.0009 |  | 0.2217 | 0.0388 | 0.0734 | 0.1098 | 0.2735 | ***0.2827*** |
| 113 | 45.5572 | -116.8352 |  | 0.0194 | -0.0053 | -0.0051 |  | 0.1980 | 0.0676 | 0.0332 | 0.0798 | ***0.3261*** | 0.2953 |
| 114 | 45.7681 | -116.7496 |  | 0.0017 | -0.0068 | -0.0040 |  | 0.1402 | 0.0654 | 0.1196 | 0.1162 | ***0.2867*** | 0.2719 |
| 115 | 45.6554 | -116.7265 |  | 0.0016 | -0.0082 | -0.0029 |  | 0.1770 | 0.0736 | 0.1106 | 0.1388 | 0.1998 | ***0.3001*** |
| 116 | 45.2019 | -116.3114 |  | 0.0013 | -0.0116 | 0.0049 |  | ***0.3251*** | 0.0406 | 0.0914 | 0.1324 | 0.3065 | 0.1042 |
| 117 | 45.1836 | -116.2995 |  | -0.0045 | -0.0100 | 0.0038 |  | 0.1751 | 0.0514 | 0.1591 | 0.1427 | ***0.3623*** | 0.1094 |
| 118 | 45.3737 | -116.3569 |  | 0.0091 | -0.0186 | 0.0091 |  | ***0.4341*** | 0.0456 | 0.0484 | 0.0722 | 0.2579 | 0.1418 |
| 119 | 45.0127 | -115.7129 |  | 0.0143 | -0.0143 | 0.0183 |  | ***0.6743*** | 0.0382 | 0.0450 | 0.0458 | 0.0648 | 0.1319 |
| 120 | 45.0692 | -115.8140 |  | 0.0038 | -0.0141 | 0.0164 |  | ***0.6100*** | 0.0464 | 0.0676 | 0.0562 | 0.0594 | 0.1604 |
| 121 | 45.0268 | -115.7082 |  | 0.0027 | -0.0162 | 0.0187 |  | ***0.6104*** | 0.0418 | 0.0692 | 0.0770 | 0.1210 | 0.0808 |
| 122 | 44.6070 | -115.6810 |  | 0.0049 | -0.0191 | 0.0192 |  | ***0.7109*** | 0.0378 | 0.0622 | 0.0612 | 0.0410 | 0.0868 |
| 123 | 45.4523 | -114.9310 |  | 0.0101 | -0.0205 | 0.0149 |  | ***0.5337*** | 0.0554 | 0.0436 | 0.0720 | 0.1484 | 0.1468 |
| 124 | 45.5716 | -115.1919 |  | 0.0156 | -0.0145 | 0.0113 |  | ***0.4686*** | 0.0610 | 0.0468 | 0.0534 | 0.2577 | 0.1125 |
| 125 | 44.8918 | -114.7222 |  | 0.0107 | -0.0216 | 0.0241 |  | ***0.8163*** | 0.0328 | 0.0396 | 0.0382 | 0.0348 | 0.0384 |
| 126 | 44.5976 | -114.8123 |  | 0.0113 | -0.0250 | 0.0318 |  | ***0.8238*** | 0.0188 | 0.0308 | 0.0290 | 0.0650 | 0.0326 |
| 127 | 45.0941 | -114.7343 |  | 0.0127 | -0.0217 | 0.0277 |  | ***0.7690*** | 0.0310 | 0.0346 | 0.0530 | 0.0662 | 0.0462 |
| 128 | 44.4493 | -115.2301 |  | 0.0096 | -0.0166 | 0.0405 |  | ***0.8780*** | 0.0256 | 0.0262 | 0.0236 | 0.0220 | 0.0246 |
| 129 | 44.7217 | -115.1488 |  | 0.0172 | -0.0225 | 0.0362 |  | ***0.8933*** | 0.0202 | 0.0238 | 0.0210 | 0.0220 | 0.0196 |
| 130 | 44.6790 | -115.1490 |  | 0.0179 | -0.0196 | 0.0328 |  | ***0.8476*** | 0.0294 | 0.0254 | 0.0272 | 0.0368 | 0.0336 |
| 131 | 44.5526 | -115.2974 |  | 0.0153 | -0.0254 | 0.0335 |  | ***0.8782*** | 0.0186 | 0.0346 | 0.0222 | 0.0204 | 0.0260 |
| 132 | 45.1523 | -115.2975 |  | 0.0141 | -0.0192 | 0.0233 |  | ***0.8225*** | 0.0246 | 0.0288 | 0.0326 | 0.0484 | 0.0432 |
| 133 | 44.8616 | -113.6319 |  | 0.0049 | 0.0007 | 0.0060 |  | 0.1154 | 0.1745 | 0.0624 | 0.1158 | ***0.4557*** | 0.0762 |
| 134 | 44.6135 | -114.1641 |  | 0.0116 | -0.0019 | 0.0011 |  | 0.0690 | 0.1820 | 0.0474 | 0.0864 | ***0.5264*** | 0.0888 |
| 135 | 45.4094 | -113.9918 |  | 0.0012 | -0.0110 | 0.0044 |  | 0.1661 | 0.0546 | 0.0952 | 0.0902 | ***0.4636*** | 0.1303 |
| 136 | 44.6844 | -114.0403 |  | 0.0085 | -0.0052 | 0.0033 |  | 0.0760 | 0.1200 | 0.0416 | 0.0960 | ***0.5856*** | 0.0808 |
| 137 | 44.1506 | -114.8851 |  | 0.0051 | -0.0081 | -0.0017 |  | 0.0660 | 0.0498 | 0.0424 | 0.0786 | ***0.6769*** | 0.0862 |
| 138 | 44.2231 | -114.9272 |  | 0.0040 | -0.0089 | -0.0002 |  | 0.0894 | 0.0430 | 0.0752 | 0.0890 | ***0.6120*** | 0.0912 |
| 139 | 44.3514 | -114.7297 |  | 0.0039 | -0.0113 | -0.0013 |  | 0.1078 | 0.0366 | 0.0636 | 0.0824 | ***0.6112*** | 0.0984 |
|  |  |  |  |  |  |  |  |  |  |  |  |  |  |
|  |  |  |  |  |  |  |  |  |  |  |  |  |  |

Table S5. Values for environmental predictor variables used in association tests to evaluate landscape genetics (see Supplemental 4). Snake River (reference) hatchery populations 140-145 were not evaluated in association tests to determine non-neutrality. All values were gathered based on population specific coordinates of latitude and longitude. Origin for migration distance (rkm) is the Columbia River estuary. Seasonal variable are: W – winter, SP – spring, SU – summer, and F – fall.

|  |  |  |  |  |  |  |  |  |  |  |  |  |  |  |  |  |  |  |  |  |
| --- | --- | --- | --- | --- | --- | --- | --- | --- | --- | --- | --- | --- | --- | --- | --- | --- | --- | --- | --- | --- |
|  | Tmax (^o^ C) | | | | |  | Tmin (^o^ C) | | | | |  | Precip. (cm) | | | | |  |  |  |
| Ref. # | mean | W | SP | SU | F |  | mean | W | SP | SU | F |  | total | W | SP | SU | F |  | Elev. (m) | (rkm) |
| 1 | 15.0 | 9.7 | 17.0 | 22.1 | 11.3 |  | 5.4 | 1.9 | 6.3 | 9.9 | 3.4 |  | 108.1 | 13.2 | 5.6 | 3.1 | 14.1 |  | 32.8 | 49.0 |
| 2 | 16.1 | 9.4 | 18.9 | 24.9 | 11.1 |  | 5.4 | 1.3 | 6.6 | 10.4 | 3.1 |  | 62.8 | 7.2 | 4.3 | 1.8 | 7.6 |  | 244.5 | 176.0 |
| 3 | 17.2 | 10.6 | 19.8 | 25.9 | 12.3 |  | 6.5 | 2.4 | 7.8 | 11.7 | 4.1 |  | 48.6 | 5.8 | 3.0 | 1.4 | 6.1 |  | 224.4 | 156.0 |
| 4 | 16.2 | 9.7 | 19.0 | 24.8 | 11.2 |  | 5.8 | 1.8 | 7.0 | 10.9 | 3.6 |  | 60.5 | 7.1 | 4.0 | 1.7 | 7.4 |  | 234.2 | 156.0 |
| 5 | 16.1 | 9.4 | 18.9 | 24.9 | 11.1 |  | 5.4 | 1.3 | 6.6 | 10.4 | 3.1 |  | 62.8 | 7.2 | 4.3 | 1.8 | 7.6 |  | 244.5 | 176.0 |
| 6 | 15.8 | 9.4 | 18.2 | 24.6 | 11.3 |  | 4.9 | 1.1 | 6.2 | 9.8 | 2.8 |  | 67.1 | 7.9 | 4.5 | 1.7 | 8.3 |  | 414.0 | 293.0 |
| 7 | 16.8 | 10.6 | 19.0 | 25.6 | 12.2 |  | 5.5 | 1.7 | 6.7 | 10.2 | 3.3 |  | 54.8 | 6.5 | 3.5 | 1.4 | 6.9 |  | 378.1 | 142.0 |
| 8 | 16.9 | 10.6 | 19.0 | 25.7 | 12.3 |  | 5.4 | 1.7 | 6.6 | 9.9 | 3.2 |  | 55.3 | 6.5 | 3.8 | 1.3 | 6.8 |  | 407.5 | 163.0 |
| 9 | 16.4 | 9.9 | 18.3 | 25.6 | 11.8 |  | 5.1 | 1.4 | 6.1 | 9.8 | 3.0 |  | 77.5 | 10.7 | 3.3 | 1.2 | 10.7 |  | 334.5 | 177.0 |
| 10 | 17.3 | 10.8 | 19.7 | 26.4 | 12.5 |  | 5.7 | 2.0 | 6.9 | 10.4 | 3.4 |  | 44.1 | 5.5 | 2.4 | 1.0 | 5.8 |  | 331.8 | 51.0 |
| 11 | 17.6 | 10.7 | 19.9 | 26.9 | 12.7 |  | 5.6 | 2.2 | 6.7 | 10.2 | 3.6 |  | 50.8 | 6.8 | 2.5 | 0.9 | 6.8 |  | 332.5 | 66.0 |
| 12 | 15.9 | 10.2 | 18.2 | 23.7 | 11.7 |  | 5.0 | 1.3 | 6.1 | 9.7 | 2.9 |  | 69.3 | 8.4 | 3.7 | 1.8 | 9.1 |  | 87.9 | 52.0 |
| 13 | 15.9 | 9.9 | 18.5 | 23.8 | 11.5 |  | 5.1 | 1.2 | 6.3 | 9.9 | 2.9 |  | 60.3 | 7.2 | 3.6 | 1.8 | 7.5 |  | 117.5 | 7.0 |
| 14 | 15.9 | 9.6 | 18.8 | 24.4 | 10.9 |  | 4.8 | 0.9 | 5.9 | 9.6 | 2.5 |  | 58.1 | 6.7 | 4.0 | 1.8 | 7.0 |  | 191.2 | 131.0 |
| 15 | 15.8 | 10.3 | 17.8 | 23.2 | 11.7 |  | 5.4 | 1.7 | 6.6 | 10.2 | 3.3 |  | 56.4 | 6.9 | 3.0 | 1.5 | 7.4 |  | 107.7 | 7.0 |
| 16 | 16.3 | 10.2 | 18.9 | 24.4 | 11.8 |  | 5.7 | 1.7 | 6.9 | 10.6 | 3.3 |  | 57.4 | 6.8 | 3.5 | 1.7 | 7.2 |  | 121.4 | 12.0 |
| 17 | 16.3 | 10.2 | 18.9 | 24.4 | 11.8 |  | 5.7 | 1.7 | 6.9 | 10.6 | 3.3 |  | 57.4 | 6.8 | 3.5 | 1.7 | 7.2 |  | 121.4 | 12.0 |
| 18 | 16.9 | 10.5 | 19.5 | 25.6 | 12.2 |  | 6.1 | 2.0 | 7.4 | 11.2 | 3.6 |  | 47.3 | 5.6 | 2.9 | 1.3 | 5.9 |  | 141.7 | 3.0 |
| 19 | 16.6 | 10.2 | 19.2 | 25.3 | 11.9 |  | 5.6 | 1.6 | 6.9 | 10.7 | 3.3 |  | 68.2 | 8.1 | 4.0 | 1.9 | 8.8 |  | 165.7 | 17.0 |
| 20 | 15.8 | 10.2 | 17.9 | 23.2 | 11.8 |  | 5.3 | 1.6 | 6.4 | 9.9 | 3.1 |  | 59.6 | 7.3 | 3.2 | 1.5 | 7.9 |  | 82.9 | 6.0 |
| 21 | 14.2 | 7.2 | 17.0 | 23.6 | 9.2 |  | 3.7 | -0.7 | 5.1 | 9.1 | 1.2 |  | 85.1 | 10.4 | 5.4 | 2.3 | 10.2 |  | 260.0 | 484.0 |
| 22 | 15.8 | 7.7 | 19.2 | 26.1 | 10.0 |  | 3.7 | -0.8 | 5.6 | 9.3 | 0.8 |  | 40.9 | 5.7 | 1.8 | 0.8 | 5.4 |  | 289.8 | 304.0 |
| 23 | 15.9 | 7.8 | 19.4 | 26.3 | 10.2 |  | 3.9 | -0.6 | 5.8 | 9.5 | 0.9 |  | 39.9 | 5.5 | 1.7 | 0.7 | 5.4 |  | 286.1 | 238.0 |
| 24 | 16.4 | 8.6 | 19.9 | 26.4 | 10.9 |  | 4.9 | 0.1 | 7.1 | 10.9 | 1.8 |  | 31.4 | 4.1 | 1.2 | 0.7 | 4.5 |  | 277.7 | 128.0 |
|  |  |  |  |  |  |  |  |  |  |  |  |  |  |  |  |  |  |  |  |  |
| 25 | 17.4 | 8.4 | 21.4 | 28.8 | 11.0 |  | 4.3 | -0.7 | 6.4 | 10.6 | 0.9 |  | 14.8 | 1.8 | 0.7 | 0.4 | 2.0 |  | 319.9 | 200.0 |
| 26 | 16.8 | 7.9 | 20.7 | 27.9 | 10.6 |  | 3.4 | -1.4 | 5.5 | 9.6 | 0.1 |  | 25.8 | 3.5 | 1.1 | 0.6 | 3.6 |  | 334.7 | 288.0 |
| 27 | 16.1 | 7.3 | 19.9 | 27.2 | 9.8 |  | 2.6 | -2.1 | 4.6 | 8.6 | -0.7 |  | 22.8 | 3.0 | 1.0 | 0.5 | 3.1 |  | 343.3 | 319.0 |
| 28 | 15.6 | 7.1 | 19.2 | 26.4 | 9.6 |  | 1.9 | -2.6 | 3.9 | 7.8 | -1.4 |  | 31.5 | 4.5 | 1.2 | 0.6 | 4.3 |  | 353.2 | 391.0 |
| 29 | 15.8 | 7.3 | 19.6 | 26.7 | 9.8 |  | 2.3 | -2.2 | 4.2 | 8.1 | -0.9 |  | 28.2 | 3.8 | 1.1 | 0.5 | 3.9 |  | 347.2 | 345.0 |
| 30 | 15.6 | 6.9 | 19.3 | 26.6 | 9.6 |  | 2.8 | -2.1 | 4.7 | 8.9 | -0.5 |  | 20.2 | 2.5 | 0.9 | 0.5 | 2.8 |  | 309.1 | 247.0 |
| 31 | 13.9 | 5.7 | 17.3 | 24.8 | 7.8 |  | 0.6 | -4.0 | 2.3 | 6.4 | -2.4 |  | 34.8 | 4.8 | 1.4 | 0.8 | 4.7 |  | 379.6 | 756.0 |
| 32 | 17.2 | 8.3 | 21.2 | 28.6 | 10.8 |  | 4.0 | -0.9 | 6.1 | 10.2 | 0.6 |  | 15.0 | 1.8 | 0.8 | 0.4 | 2.0 |  | 315.1 | 216.0 |
| 33 | 14.9 | 6.5 | 18.5 | 25.9 | 8.7 |  | 1.3 | -3.1 | 3.1 | 7.1 | -1.7 |  | 31.6 | 4.5 | 1.2 | 0.6 | 4.3 |  | 359.2 | 590.0 |
| 34 | 17.6 | 8.6 | 21.7 | 28.9 | 11.2 |  | 4.6 | -0.5 | 6.8 | 10.9 | 1.2 |  | 14.2 | 1.7 | 0.7 | 0.4 | 1.9 |  | 315.7 | 175.0 |
| 35 | 17.2 | 8.4 | 21.2 | 28.4 | 10.7 |  | 3.9 | -1.4 | 6.1 | 10.7 | 0.2 |  | 11.9 | 1.7 | 0.6 | 0.3 | 1.5 |  | 393.2 | 234.0 |
| 36 | 18.8 | 10.0 | 22.6 | 30.2 | 12.6 |  | 2.7 | -2.2 | 4.7 | 8.9 | -0.7 |  | 10.9 | 1.2 | 0.8 | 0.4 | 1.2 |  | 483.1 | 428.0 |
| 37 | 18.2 | 9.4 | 21.9 | 29.7 | 11.9 |  | 2.4 | -2.4 | 4.4 | 8.7 | -0.9 |  | 10.9 | 1.2 | 0.8 | 0.5 | 1.2 |  | 487.2 | 449.0 |
| 38 | 18.8 | 9.9 | 22.5 | 30.1 | 12.4 |  | 2.6 | -2.2 | 4.7 | 8.8 | -0.7 |  | 10.6 | 1.2 | 0.8 | 0.4 | 1.2 |  | 478.0 | 418.0 |
| 39 | 19.1 | 10.2 | 23.0 | 30.6 | 12.7 |  | 2.8 | -2.1 | 4.8 | 9.1 | -0.6 |  | 11.0 | 1.1 | 0.9 | 0.4 | 1.2 |  | 462.6 | 394.0 |
| 40 | 17.8 | 9.0 | 21.9 | 28.8 | 11.5 |  | 5.1 | 0.0 | 7.4 | 11.5 | 1.6 |  | 15.7 | 2.0 | 0.7 | 0.4 | 2.1 |  | 310.7 | 109.0 |
| 41 | 11.2 | 3.2 | 13.4 | 22.5 | 5.7 |  | 0.5 | -5.2 | 1.3 | 8.3 | -2.6 |  | 28.9 | 3.6 | 2.1 | 0.8 | 3.1 |  | 706.3 | 1698.0 |
| 42 | 16.4 | 7.3 | 19.8 | 28.4 | 10.3 |  | 1.2 | -4.1 | 3.6 | 7.5 | -2.4 |  | 16.2 | 1.4 | 1.6 | 0.9 | 1.5 |  | 736.0 | 993.0 |
| 43 | 19.3 | 10.4 | 23.1 | 30.8 | 13.0 |  | 3.6 | -1.3 | 5.9 | 9.9 | -0.3 |  | 11.6 | 1.0 | 1.2 | 0.5 | 1.2 |  | 560.5 | 456.0 |
| 44 | 14.5 | 5.7 | 17.7 | 26.2 | 8.4 |  | -0.9 | -6.6 | 1.8 | 5.7 | -4.7 |  | 20.6 | 1.9 | 1.8 | 1.1 | 2.0 |  | 775.7 | 1217.0 |
| 45 | 14.2 | 5.2 | 17.5 | 26.0 | 8.0 |  | -1.6 | -7.1 | 1.3 | 4.8 | -5.2 |  | 20.5 | 2.1 | 1.6 | 0.9 | 2.2 |  | 789.5 | 1215.0 |
| 46 | 18.5 | 9.4 | 22.0 | 30.2 | 12.3 |  | 2.3 | -2.6 | 4.8 | 8.7 | -1.6 |  | 15.4 | 1.6 | 1.4 | 0.7 | 1.5 |  | 674.6 | 630.0 |
| 47 | 12.2 | 3.3 | 15.2 | 24.0 | 6.1 |  | -2.3 | -8.4 | 0.5 | 4.4 | -5.4 |  | 23.3 | 2.6 | 1.9 | 0.9 | 2.4 |  | 786.8 | 1341.0 |
| 48 | 15.1 | 6.6 | 17.9 | 26.3 | 9.4 |  | 0.5 | -4.4 | 2.8 | 6.4 | -2.7 |  | 18.0 | 1.7 | 1.6 | 0.7 | 1.9 |  | 730.1 | 864.0 |
| 49 | 14.5 | 5.8 | 17.5 | 26.1 | 8.6 |  | 0.2 | -5.1 | 2.2 | 6.7 | -3.3 |  | 16.3 | 1.6 | 1.4 | 0.8 | 1.6 |  | 729.5 | 1221.0 |
| 50 | 14.3 | 5.7 | 17.2 | 25.8 | 8.4 |  | -1.2 | -6.4 | 1.0 | 4.7 | -4.2 |  | 15.7 | 1.4 | 1.5 | 0.7 | 1.6 |  | 733.4 | 1331.0 |
| 51 | 17.1 | 8.0 | 21.2 | 28.6 | 10.6 |  | 3.6 | -1.4 | 5.7 | 10.2 | 0.1 |  | 14.1 | 1.7 | 0.7 | 0.4 | 1.9 |  | 381.2 | 258.0 |
| 52 | 16.9 | 7.9 | 20.9 | 28.3 | 10.3 |  | 3.5 | -1.6 | 5.6 | 10.1 | -0.1 |  | 14.0 | 1.7 | 0.7 | 0.4 | 1.9 |  | 380.8 | 252.0 |
| 53 | 17.0 | 8.2 | 20.8 | 28.5 | 10.4 |  | 3.7 | -1.5 | 6.3 | 10.3 | -0.2 |  | 27.5 | 3.0 | 2.3 | 0.9 | 3.0 |  | 578.7 | 514.0 |
| 54 | 15.0 | 6.2 | 18.7 | 26.6 | 8.6 |  | 1.9 | -3.3 | 4.3 | 8.1 | -1.6 |  | 32.0 | 3.5 | 2.6 | 1.1 | 3.5 |  | 599.1 | 719.0 |
| 55 | 18.3 | 9.3 | 22.9 | 29.8 | 11.3 |  | 5.2 | -0.8 | 8.2 | 12.5 | 0.8 |  | 11.8 | 1.2 | 1.0 | 0.5 | 1.3 |  | 530.6 | 135.0 |
| 56 | 11.2 | 3.4 | 13.8 | 21.6 | 5.7 |  | -0.3 | -5.4 | 1.1 | 6.0 | -3.1 |  | 45.3 | 5.7 | 2.1 | 1.3 | 6.1 |  | 816.0 | 997.0 |
| 57 | 14.6 | 5.6 | 18.7 | 26.1 | 7.9 |  | 1.6 | -4.3 | 4.2 | 8.8 | -2.6 |  | 19.7 | 2.3 | 1.0 | 0.6 | 2.7 |  | 768.9 | 611.0 |
| 58 | 12.1 | 4.0 | 15.2 | 23.0 | 6.4 |  | -0.3 | -5.8 | 1.7 | 6.3 | -3.6 |  | 38.3 | 4.8 | 1.7 | 1.0 | 5.3 |  | 804.9 | 878.0 |
| 59 | 12.7 | 4.3 | 16.1 | 23.9 | 6.7 |  | 0.2 | -5.4 | 2.4 | 6.9 | -3.1 |  | 36.2 | 4.6 | 1.6 | 0.9 | 5.0 |  | 799.6 | 831.0 |
| 60 | 14.3 | 5.4 | 18.4 | 25.8 | 7.7 |  | 1.4 | -4.6 | 4.1 | 8.6 | -2.7 |  | 18.7 | 2.2 | 0.9 | 0.6 | 2.5 |  | 766.9 | 601.0 |
| 61 | 18.3 | 8.9 | 23.4 | 29.9 | 11.1 |  | 4.6 | -1.5 | 7.8 | 12.0 | -0.2 |  | 7.5 | 0.8 | 0.5 | 0.3 | 0.9 |  | 643.5 | 200.0 |
| 62 | 18.4 | 8.9 | 23.5 | 29.9 | 11.1 |  | 4.4 | -1.7 | 7.6 | 11.9 | -0.4 |  | 7.5 | 0.7 | 0.5 | 0.3 | 0.9 |  | 661.0 | 208.0 |
| 63 | 14.6 | 5.2 | 19.5 | 26.7 | 7.2 |  | 1.4 | -4.5 | 3.9 | 8.5 | -2.3 |  | 33.2 | 4.3 | 1.2 | 0.7 | 4.9 |  | 801.2 | 554.0 |
| 64 | 16.2 | 6.2 | 21.5 | 28.7 | 8.6 |  | 2.9 | -3.4 | 5.8 | 10.4 | -1.2 |  | 22.0 | 2.8 | 0.9 | 0.5 | 3.1 |  | 785.3 | 346.0 |
| 65 | 16.2 | 6.2 | 21.5 | 28.7 | 8.6 |  | 2.9 | -3.4 | 5.8 | 10.4 | -1.2 |  | 22.0 | 2.8 | 0.9 | 0.5 | 3.1 |  | 785.3 | 346.0 |
| 66 | 14.4 | 5.1 | 19.3 | 26.2 | 7.1 |  | 1.2 | -4.8 | 3.8 | 8.2 | -2.6 |  | 29.8 | 3.8 | 1.2 | 0.7 | 4.3 |  | 829.3 | 573.0 |
| 67 | 15.2 | 5.4 | 20.3 | 27.4 | 7.7 |  | 2.4 | -4.0 | 5.3 | 10.2 | -1.9 |  | 29.9 | 3.9 | 1.1 | 0.6 | 4.4 |  | 790.5 | 451.0 |
| 68 | 11.6 | 3.2 | 15.6 | 22.9 | 4.8 |  | 0.7 | -5.1 | 2.6 | 8.1 | -3.1 |  | 49.4 | 6.4 | 1.9 | 1.2 | 7.1 |  | 807.6 | 854.0 |
| 69 | 16.1 | 5.9 | 21.7 | 28.7 | 8.2 |  | 3.1 | -3.8 | 6.4 | 11.2 | -1.6 |  | 13.4 | 1.6 | 0.8 | 0.4 | 1.7 |  | 777.4 | 309.0 |
| 70 | 14.9 | 4.3 | 20.8 | 27.8 | 6.6 |  | 0.3 | -6.9 | 4.1 | 8.4 | -4.6 |  | 14.6 | 1.5 | 1.0 | 0.7 | 1.7 |  | 914.4 | 531.0 |
| 71 | 16.1 | 5.9 | 22.4 | 28.3 | 7.9 |  | 3.4 | -3.4 | 6.9 | 11.4 | -1.2 |  | 12.3 | 1.0 | 1.3 | 0.7 | 1.2 |  | 938.5 | 287.0 |
| 72 | 16.1 | 5.4 | 22.3 | 29.1 | 7.7 |  | 2.7 | -4.3 | 6.4 | 11.0 | -2.3 |  | 12.7 | 1.2 | 1.1 | 0.7 | 1.3 |  | 899.1 | 286.0 |
| 73 | 16.1 | 5.3 | 22.2 | 29.0 | 7.6 |  | 2.7 | -4.4 | 6.4 | 11.0 | -2.3 |  | 12.4 | 1.2 | 1.1 | 0.6 | 1.3 |  | 889.7 | 322.0 |
| 74 | 15.2 | 6.1 | 19.2 | 27.0 | 8.6 |  | 2.9 | -2.3 | 5.3 | 9.5 | -0.8 |  | 25.6 | 2.8 | 2.0 | 1.0 | 2.8 |  | 672.3 | 678.0 |
| 75 | 18.6 | 9.2 | 23.2 | 30.8 | 10.9 |  | 5.3 | -0.3 | 8.0 | 12.4 | 1.1 |  | 15.6 | 1.5 | 1.4 | 0.7 | 1.6 |  | 720.7 | 251.0 |
| 76 | 17.6 | 8.6 | 22.1 | 29.7 | 10.2 |  | 4.6 | -0.9 | 7.2 | 11.4 | 0.5 |  | 15.8 | 1.6 | 1.4 | 0.7 | 1.6 |  | 749.6 | 318.0 |
| 77 | 18.6 | 9.2 | 23.2 | 31.0 | 10.9 |  | 5.2 | -0.4 | 7.9 | 12.4 | 1.0 |  | 13.4 | 1.3 | 1.2 | 0.6 | 1.4 |  | 741.7 | 227.0 |
| 78 | 18.7 | 9.3 | 23.3 | 31.0 | 11.1 |  | 5.1 | -0.5 | 7.9 | 12.0 | 0.9 |  | 18.8 | 1.7 | 1.8 | 0.9 | 1.9 |  | 769.6 | 246.0 |
| 79 | 17.7 | 8.6 | 22.1 | 29.8 | 10.2 |  | 4.6 | -0.9 | 7.2 | 11.4 | 0.5 |  | 15.8 | 1.6 | 1.4 | 0.7 | 1.6 |  | 749.7 | 333.0 |
| 80 | 17.4 | 8.2 | 21.9 | 29.7 | 9.8 |  | 4.3 | -1.3 | 6.9 | 11.2 | 0.2 |  | 21.9 | 2.1 | 2.0 | 1.0 | 2.2 |  | 766.2 | 405.0 |
| 81 | 16.9 | 7.6 | 21.3 | 29.1 | 9.6 |  | 3.9 | -1.6 | 6.6 | 10.5 | -0.1 |  | 22.6 | 2.2 | 2.0 | 1.0 | 2.4 |  | 780.5 | 398.0 |
| 82 | 13.6 | 4.4 | 17.7 | 25.5 | 6.6 |  | 0.6 | -4.5 | 3.2 | 6.6 | -3.0 |  | 33.8 | 3.7 | 2.5 | 1.4 | 3.6 |  | 820.7 | 822.0 |
| 83 | 16.8 | 7.5 | 21.1 | 28.9 | 9.4 |  | 3.9 | -1.5 | 6.6 | 10.7 | -0.1 |  | 22.8 | 2.2 | 2.0 | 1.0 | 2.4 |  | 782.6 | 453.0 |
| 84 | 13.7 | 4.6 | 17.8 | 25.6 | 6.7 |  | 0.7 | -4.4 | 3.3 | 6.7 | -2.9 |  | 33.8 | 3.7 | 2.5 | 1.4 | 3.6 |  | 821.1 | 827.0 |
| 85 | 15.8 | 6.2 | 20.7 | 28.2 | 7.9 |  | 0.9 | -5.0 | 4.1 | 7.7 | -3.2 |  | 29.4 | 2.7 | 2.8 | 1.6 | 2.8 |  | 969.7 | 763.0 |
| 86 | 16.1 | 6.7 | 20.8 | 28.7 | 8.2 |  | 2.2 | -3.4 | 5.2 | 8.8 | -1.6 |  | 36.2 | 3.6 | 3.2 | 1.5 | 3.7 |  | 916.8 | 521.0 |
| 87 | 13.1 | 4.2 | 17.2 | 24.9 | 5.9 |  | -1.8 | -7.8 | 1.1 | 5.1 | -5.6 |  | 30.3 | 2.6 | 3.0 | 1.7 | 2.7 |  | 1011.1 | 1178.0 |
| 88 | 13.4 | 4.1 | 17.7 | 25.7 | 6.0 |  | -2.3 | -8.7 | 1.2 | 4.7 | -6.6 |  | 29.4 | 2.6 | 2.9 | 1.7 | 2.6 |  | 1020.0 | 1268.0 |
| 89 | 16.1 | 6.3 | 21.1 | 28.7 | 8.0 |  | 1.7 | -3.9 | 4.6 | 8.2 | -2.2 |  | 31.8 | 3.0 | 3.0 | 1.7 | 3.0 |  | 958.1 | 700.0 |
| 90 | 16.1 | 6.7 | 20.9 | 28.5 | 8.3 |  | 2.6 | -3.1 | 5.3 | 9.1 | -1.1 |  | 36.6 | 3.7 | 3.2 | 1.6 | 3.8 |  | 899.8 | 482.0 |
| 91 | 15.9 | 6.3 | 20.7 | 28.6 | 8.0 |  | 1.4 | -4.2 | 4.4 | 8.0 | -2.4 |  | 31.5 | 3.0 | 3.0 | 1.5 | 3.0 |  | 939.1 | 583.0 |
| 92 | 13.7 | 4.5 | 18.3 | 25.9 | 6.3 |  | -1.1 | -6.9 | 2.1 | 5.8 | -5.0 |  | 34.5 | 3.4 | 3.0 | 1.6 | 3.5 |  | 997.1 | 964.0 |
| 93 | 15.3 | 6.2 | 19.8 | 27.5 | 7.8 |  | 2.2 | -3.6 | 5.0 | 9.2 | -1.9 |  | 34.8 | 3.4 | 3.1 | 1.6 | 3.5 |  | 901.5 | 591.0 |
| 94 | 12.4 | 3.8 | 16.3 | 23.9 | 5.5 |  | -2.4 | -8.4 | 0.7 | 4.2 | -6.2 |  | 43.7 | 4.7 | 3.6 | 1.7 | 4.6 |  | 1018.6 | 1308.0 |
| 95 | 13.5 | 4.3 | 17.8 | 25.7 | 6.3 |  | -1.1 | -7.0 | 2.0 | 5.7 | -5.1 |  | 40.6 | 4.2 | 3.5 | 1.6 | 4.2 |  | 1000.7 | 1084.0 |
| 96 | 15.6 | 6.4 | 20.2 | 27.9 | 8.0 |  | 1.9 | -3.7 | 4.8 | 8.4 | -1.9 |  | 36.4 | 3.6 | 3.2 | 1.6 | 3.7 |  | 926.7 | 616.0 |
| 97 | 13.9 | 4.6 | 18.2 | 25.9 | 6.7 |  | -0.7 | -6.5 | 2.3 | 6.0 | -4.6 |  | 37.1 | 3.8 | 3.1 | 1.6 | 3.9 |  | 966.2 | 875.0 |
| 98 | 12.0 | 3.4 | 15.9 | 23.6 | 5.1 |  | -2.2 | -8.1 | 0.7 | 4.6 | -6.1 |  | 43.8 | 4.8 | 3.6 | 1.7 | 4.6 |  | 1014.5 | 1280.0 |
| 99 | 14.6 | 5.6 | 18.5 | 26.6 | 7.7 |  | 1.8 | -3.9 | 4.7 | 8.8 | -2.4 |  | 30.5 | 2.7 | 3.3 | 1.5 | 2.7 |  | 877.2 | 785.0 |
| 100 | 13.7 | 4.9 | 17.1 | 25.6 | 6.9 |  | -2.3 | -7.9 | 0.6 | 4.2 | -6.1 |  | 32.5 | 3.0 | 3.1 | 1.8 | 3.0 |  | 945.5 | 1170.0 |
| 101 | 15.3 | 6.5 | 19.3 | 27.3 | 8.2 |  | 1.4 | -4.2 | 4.2 | 8.1 | -2.6 |  | 26.9 | 2.1 | 3.3 | 1.5 | 2.1 |  | 907.4 | 740.0 |
| 102 | 13.9 | 5.1 | 17.7 | 25.9 | 7.1 |  | -1.1 | -6.7 | 1.7 | 5.4 | -4.9 |  | 31.5 | 2.9 | 3.1 | 1.7 | 2.8 |  | 926.1 | 1054.0 |
| 103 | 18.4 | 9.0 | 22.6 | 30.8 | 11.2 |  | 4.4 | -0.9 | 7.0 | 11.3 | 0.4 |  | 18.0 | 1.2 | 2.3 | 1.3 | 1.3 |  | 916.4 | 472.0 |
| 104 | 17.6 | 8.3 | 21.6 | 29.8 | 10.4 |  | 3.8 | -1.5 | 6.3 | 10.5 | -0.2 |  | 17.3 | 1.2 | 2.2 | 1.2 | 1.2 |  | 896.9 | 441.0 |
| 105 | 16.8 | 7.5 | 20.9 | 28.8 | 9.8 |  | 2.6 | -2.5 | 5.2 | 8.8 | -1.1 |  | 22.5 | 2.2 | 1.9 | 0.8 | 2.6 |  | 862.9 | 594.0 |
| 106 | 15.2 | 6.0 | 18.9 | 27.2 | 8.8 |  | -0.8 | -5.9 | 1.9 | 5.2 | -4.5 |  | 19.1 | 1.5 | 2.0 | 1.2 | 1.7 |  | 865.3 | 1002.0 |
| 107 | 18.7 | 9.5 | 22.9 | 30.9 | 11.7 |  | 4.8 | -0.6 | 7.4 | 11.7 | 0.8 |  | 16.3 | 1.3 | 1.7 | 0.9 | 1.5 |  | 790.2 | 307.0 |
| 108 | 14.6 | 5.7 | 18.0 | 26.1 | 8.4 |  | -0.1 | -5.1 | 2.5 | 5.8 | -3.6 |  | 28.4 | 2.8 | 2.7 | 1.4 | 2.7 |  | 955.0 | 1039.0 |
| 109 | 15.5 | 6.2 | 19.4 | 27.4 | 9.0 |  | 0.3 | -4.8 | 3.1 | 6.2 | -3.4 |  | 17.4 | 1.5 | 1.5 | 1.0 | 1.7 |  | 950.8 | 918.0 |
| 110 | 17.6 | 8.6 | 21.7 | 29.5 | 10.7 |  | 3.7 | -1.7 | 6.3 | 10.2 | -0.2 |  | 20.5 | 2.0 | 1.8 | 0.9 | 2.2 |  | 836.9 | 437.0 |
| 111 | 17.5 | 8.3 | 21.7 | 29.5 | 10.6 |  | 3.3 | -1.8 | 6.0 | 9.7 | -0.6 |  | 21.1 | 2.2 | 1.8 | 0.8 | 2.3 |  | 852.0 | 486.0 |
| 112 | 17.7 | 8.7 | 21.4 | 29.4 | 11.4 |  | 3.2 | -2.2 | 6.0 | 9.9 | -0.8 |  | 16.4 | 1.1 | 1.9 | 1.3 | 1.2 |  | 854.5 | 598.0 |
| 113 | 17.7 | 8.7 | 21.4 | 29.4 | 11.4 |  | 3.2 | -2.2 | 6.0 | 9.9 | -0.8 |  | 16.4 | 1.1 | 1.9 | 1.3 | 1.2 |  | 856.5 | 623.0 |
| 114 | 19.2 | 9.7 | 23.2 | 31.4 | 12.3 |  | 4.7 | -0.7 | 7.3 | 11.3 | 0.7 |  | 15.7 | 1.1 | 2.0 | 1.1 | 1.1 |  | 823.7 | 365.0 |
| 115 | 16.1 | 6.6 | 20.0 | 28.1 | 9.4 |  | 2.7 | -2.6 | 5.4 | 9.5 | -1.4 |  | 17.7 | 1.2 | 2.0 | 1.4 | 1.3 |  | 837.3 | 727.0 |
| 116 | 15.4 | 5.3 | 19.8 | 28.1 | 8.7 |  | 0.8 | -6.0 | 4.2 | 8.6 | -3.3 |  | 24.0 | 2.4 | 2.3 | 1.1 | 2.3 |  | 976.6 | 938.0 |
| 117 | 15.5 | 5.3 | 19.8 | 28.2 | 8.7 |  | 0.9 | -6.0 | 4.2 | 8.6 | -3.4 |  | 24.0 | 2.5 | 2.2 | 1.1 | 2.3 |  | 978.9 | 976.0 |
| 118 | 17.7 | 8.3 | 22.1 | 29.8 | 10.7 |  | 3.4 | -2.4 | 6.3 | 10.1 | -0.4 |  | 16.1 | 1.3 | 1.9 | 0.9 | 1.3 |  | 955.1 | 601.0 |
| 119 | 14.6 | 4.5 | 18.9 | 27.5 | 7.3 |  | -1.2 | -7.7 | 2.3 | 5.8 | -5.3 |  | 22.0 | 2.3 | 1.9 | 1.0 | 2.2 |  | 1082.2 | 1122.0 |
| 120 | 12.4 | 3.1 | 16.2 | 24.7 | 5.8 |  | -2.9 | -9.5 | 0.4 | 4.1 | -6.8 |  | 30.0 | 3.1 | 2.6 | 1.4 | 2.9 |  | 1093.5 | 1361.0 |
| 121 | 14.7 | 4.7 | 19.2 | 27.7 | 7.4 |  | -1.1 | -7.5 | 2.5 | 5.9 | -5.1 |  | 21.8 | 2.2 | 1.9 | 1.0 | 2.1 |  | 1080.9 | 1107.0 |
| 122 | 11.8 | 2.5 | 15.4 | 24.2 | 5.1 |  | -4.3 | -10.8 | -0.8 | 2.5 | -8.2 |  | 30.0 | 3.4 | 2.2 | 1.1 | 3.2 |  | 1146.1 | 1619.0 |
| 123 | 15.2 | 5.1 | 20.0 | 28.4 | 7.1 |  | -0.1 | -6.6 | 3.3 | 7.4 | -4.4 |  | 26.1 | 2.2 | 2.7 | 1.6 | 2.2 |  | 1089.0 | 846.0 |
| 124 | 15.3 | 5.5 | 20.1 | 28.4 | 7.4 |  | 0.1 | -6.3 | 3.6 | 7.2 | -4.0 |  | 23.4 | 2.0 | 2.3 | 1.4 | 2.1 |  | 1062.7 | 781.0 |
| 125 | 15.2 | 4.8 | 20.3 | 28.6 | 7.2 |  | -0.4 | -7.3 | 3.3 | 7.6 | -5.1 |  | 16.3 | 1.5 | 1.6 | 0.9 | 1.5 |  | 1182.7 | 1153.0 |
| 126 | 12.7 | 3.3 | 16.6 | 25.1 | 5.7 |  | -3.6 | -10.5 | -0.1 | 4.2 | -7.9 |  | 26.2 | 2.8 | 2.2 | 1.2 | 2.6 |  | 1232.1 | 1646.0 |
| 127 | 14.8 | 4.5 | 19.9 | 28.2 | 6.5 |  | -0.3 | -7.2 | 3.3 | 7.4 | -4.9 |  | 16.4 | 1.2 | 1.9 | 1.1 | 1.3 |  | 1209.0 | 1621.0 |
| 128 | 10.7 | 1.9 | 14.0 | 22.7 | 4.4 |  | -5.6 | -12.4 | -2.6 | 2.0 | -9.5 |  | 41.6 | 5.2 | 2.8 | 1.3 | 4.6 |  | 1294.6 | 1890.0 |
| 129 | 13.9 | 4.3 | 17.9 | 26.5 | 6.8 |  | -2.2 | -8.8 | 1.3 | 5.2 | -6.4 |  | 21.6 | 2.2 | 1.8 | 1.1 | 2.2 |  | 1243.6 | 1455.0 |
| 130 | 13.2 | 3.6 | 17.2 | 25.8 | 6.2 |  | -2.5 | -9.2 | 0.9 | 5.0 | -6.7 |  | 23.9 | 2.2 | 2.1 | 1.2 | 2.4 |  | 1249.6 | 1504.0 |
| 131 | 12.3 | 3.3 | 15.7 | 24.4 | 5.6 |  | -4.8 | -11.4 | -1.3 | 2.2 | -8.8 |  | 33.9 | 4.0 | 2.4 | 1.2 | 3.7 |  | 1275.8 | 1702.0 |
| 132 | 12.0 | 3.1 | 15.3 | 24.1 | 5.6 |  | -5.2 | -11.4 | -1.7 | 0.8 | -8.7 |  | 29.5 | 3.2 | 2.4 | 1.5 | 2.8 |  | 1209.0 | 1621.0 |
| 133 | 13.6 | 3.6 | 18.2 | 26.3 | 6.2 |  | -2.2 | -9.6 | 1.8 | 6.1 | -7.2 |  | 10.4 | 0.6 | 1.3 | 0.9 | 0.7 |  | 1274.2 | 1589.0 |
| 134 | 15.1 | 4.4 | 20.3 | 28.4 | 7.4 |  | -1.3 | -8.6 | 2.8 | 7.1 | -6.4 |  | 8.1 | 0.4 | 1.0 | 0.8 | 0.6 |  | 1312.4 | 1457.0 |
| 135 | 15.6 | 4.8 | 21.0 | 29.1 | 7.3 |  | -0.4 | -7.4 | 3.7 | 7.6 | -5.2 |  | 12.1 | 0.8 | 1.4 | 0.9 | 0.9 |  | 1188.6 | 1109.0 |
| 136 | 15.3 | 4.7 | 20.3 | 28.6 | 7.7 |  | -1.3 | -8.6 | 2.7 | 6.8 | -6.3 |  | 8.8 | 0.4 | 1.2 | 0.8 | 0.6 |  | 1298.5 | 1423.0 |
| 137 | 11.3 | 2.2 | 14.7 | 23.4 | 4.9 |  | -6.6 | -14.3 | -2.3 | 1.1 | -10.9 |  | 20.7 | 2.3 | 1.6 | 0.9 | 2.1 |  | 1439.0 | 1979.0 |
| 138 | 11.7 | 2.4 | 15.3 | 23.9 | 5.1 |  | -6.6 | -14.2 | -2.2 | 1.0 | -10.9 |  | 17.5 | 1.8 | 1.4 | 0.8 | 1.8 |  | 1427.4 | 1900.0 |
| 139 | 11.8 | 3.1 | 14.8 | 23.7 | 5.4 |  | -6.0 | -13.4 | -1.9 | 1.7 | -10.4 |  | 22.3 | 2.3 | 1.9 | 1.0 | 2.2 |  | 1418.3 | 1918.0 |
|  |  |  |  |  |  |  |  |  |  |  |  |  |  |  |  |  |  |  |  |  |
|  |  |  |  |  |  |  |  |  |  |  |  |  |  |  |  |  |  |  |  |  |

Table S6. List of final locus classifications based on significance in association tests. The list includes only loci that met at least one of the four candidate criteria (see methods section), and neutral loci listed are only those that were initially flagged as possible candidates. Outlier loci are marker by “X”, and numbers in parentheses refer to landscape association precedence (referenced in Table 2). Regression significance (*) is adjusted (P<0.00029), and “-” indicates non-significant. Candidate loci were used in analysis of non-neutral differentiation, neutral loci were added to the existing panel of neutral loci for analysis of neutral differentiation, and “ambiguous” loci were not included in either analysis. Seasonal variable are: W – winter, SP – spring, SU – summer, and F – fall.

|  |  |  |  |  |  |  |  |  |  |  |  |  |  |  |  |  |  |  |  |  |  |  |  |
| --- | --- | --- | --- | --- | --- | --- | --- | --- | --- | --- | --- | --- | --- | --- | --- | --- | --- | --- | --- | --- | --- | --- | --- |
| Linear Regression |  |  |  |  |  |  |  |  |  |  |  |  |  |  |  |  |  |  |  |  |  |  |  |
|  |  | Tmax | |  |  |  |  | Tmin |  |  |  |  |  | Precip. | |  |  |  |  |  |  |  |  |
| SNP |  | mean | W | SP | SU | F |  | mean | W | SP | SU | F |  | total | W | SP | SU | F |  | Elev. | (rkm) | lat | Long |
|  |  |  |  |  |  |  |  |  |  |  |  |  |  |  |  |  |  |  |  |  |  |  |  |
| **INLAND** |  |  |  |  |  |  |  |  |  |  |  |  |  |  |  |  |  |  |  |  |  |  |  |
|  |  |  |  |  |  |  |  |  |  |  |  |  |  |  |  |  |  |  |  |  |  |  |  |
| **Candidate Loci** |  |  |  |  |  |  |  |  |  |  |  |  |  |  |  |  |  |  |  |  |  |  |  |
| Omy_97660-230 |  | - | - | - | - | - |  | - | ******* | - | - | ******* |  | - | - | - | - | - |  | - | ******* | - | ******* |
| Omy_e1-147 |  | - | - | - | - | - |  | - | - | - | - | - |  | - | - | - | - | - |  | - | ******* | - | ******* |
| Omy_hsc715-80 |  | - | - | - | - | - |  | ******* | - | - | ******* | - |  | - | - | ******* | ******* | - |  | ******* | ******* | - | ******* |
| Omy_hsp47-86 | (1) | - | - | - | - | ******* |  | ******* | ******* | - | - | ******* |  | - | - | - | - | - |  | ******* | ******* | - | ******* |
| Omy_ntl-27 |  | - | - | - | - | - |  | - | - | - | - | - |  | - | - | - | - | - |  | - | ******* | - | ******* |
| Omy_SECC22b-88 |  | - | - | ******* | - | - |  | - | - | - | - | - |  | ******* | - | ******* | - | - |  | - | - | ******* | - |
| OMS00014 |  | - | - | - | - | - |  | - | - | - | - | - |  | ******* | - | ******* | - | - |  | - | - | - | - |
| OMS00062 |  | - | - | - | - | - |  | - | - | - | - | - |  | ******* | ******* | - | - | ******* |  | - | - | - | - |
| Omy_97954-618 |  | - | - | - | - | - |  | - | - | - | - | - |  | - | - | - | ******* | - |  | - | ******* | - | ******* |
| Omy_CRBF1-1 |  | - | - | - | - | - |  | - | - | - | - | - |  | - | - | ******* | ******* | - |  | - | ******* | - | ******* |
| Omy_gdh-271 | (2) | - | - | - | - | - |  | - | - | - | - | - |  | - | - | - | - | - |  | - | - | - | - |
| Omy_GHSR-121 |  | - | - | - | - | - |  | - | - | - | - | - |  | ******* | - | ******* | ******* | - |  | - | - | - | ******* |
| Omy_IL6-320 |  | - | - | - | - | - |  | - | - | - | - | - |  | ******* | ******* | - | - | ******* |  | - | - | - | - |
| Omy_nkef-241 |  | - | - | - | - | - |  | - | - | - | - | - |  | - | - | - | - | - |  | - | ******* | - | ******* |
| Omy_OmyP9-180 | (1) | ******* | - | ******* | ******* | - |  | ******* | - | ******* | ******* | - |  | - | - | - | - | - |  | ******* | - | - | - |
| Omy_tlr5-205 | (2) | - | - | - | - | - |  | - | ******* | - | - | ******* |  | - | - | - | ******* | - |  | ******* | ******* | - | ******* |
| Omy_u09-53.469 | X | - | ******* | - | - | ******* |  | ******* | ******* | ******* | ******* | ******* |  | - | - | ******* | ******* | - |  | ******* | ******* | - | ******* |
| Omy_UT16_2-173 | X | - | - | - | - | - |  | - | - | - | - | - |  | - | - | - | - | - |  | - | ******* | - | - |
| OMY1011SNP |  | - | - | - | - | - |  | - | - | - | - | - |  | - | - | ******* | - | - |  | - | - | - | ******* |
| OMS00151 |  | ******* | - | - | - | ******* |  | - | - | ******* | - | - |  | - | - | - | - | - |  | - | - | - | - |
| Omy_metA-161 |  | - | - | - | - | - |  | - | - | - | - | - |  | ******* | - | - | - | - |  | - | - | ******* | - |
| Omy_stat3-273 | (2) | - | - | - | - | - |  | - | - | - | - | - |  | - | - | - | - | - |  | - | - | - | - |
|  |  |  |  |  |  |  |  |  |  |  |  |  |  |  |  |  |  |  |  |  |  |  |  |
| **"Ambiguous"** |  |  |  |  |  |  |  |  |  |  |  |  |  |  |  |  |  |  |  |  |  |  |  |
| Omy_anp-17 | X | - | - | - | - | - |  | - | - | - | - | - |  | ******* | - | - | - | - |  | - | - | - | - |
| Omy_b9-164 | X | - | - | - | - | - |  | - | - | - | - | - |  | ******* | - | - | - | - |  | - | - | - | ******* |
| Omy_IL1b-163 | X | - | - | - | - | - |  | - | - | - | - | - |  | - | ******* | - | ******* | - |  | - | ******* | - | - |
| Omy_ndk-152 | X; (3) | - | - | - | - | - |  | - | - | - | - | - |  | - | - | ******* | ******* | - |  | ******* | ******* | - | ******* |
| Omy_nxt2-273 | X | - | - | - | - | - |  | - | - | - | - | - |  | - | - | - | - | - |  | - | ******* | - | - |
| Omy_star-206 | X | ******* | ******* | ******* | - | ******* |  | ******* | ******* | ******* | ******* | ******* |  | ******* | - | ******* | ******* | - |  | ******* | ******* | - | ******* |
| Omy_u09-56.119 | X | - | - | - | - | - |  | - | - | - | - | - |  | - | - | ******* | ******* | - |  | ******* | ******* | - | ******* |
| Omy_vatf-406 | X | ******* | ******* | - | - | ******* |  | - | ******* | - | - | ******* |  | ******* | - | ******* | ******* | - |  | - | ******* | - | ******* |
| Omy_LDHB-2_i6 | (3) | - | - | - | - | - |  | - | - | - | - | - |  | - | - | - | - | - |  | - | ******* | - | ******* |
| Omy_hsf2-146 | (4) | - | - | - | - | - |  | - | - | - | - | - |  | - | - | - | ******* | - |  | - | - | ******* | - |
| Omy_aldB-165 | (2) | - | - | - | - | - |  | - | - | - | - | - |  | - | - | - | - | - |  | - | - | - | - |
| Omy_Ogo4-212 | (2) | - | - | - | - | - |  | - | - | - | - | - |  | - | - | - | - | - |  | - | - | ******* | ******* |
|  |  |  |  |  |  |  |  |  |  |  |  |  |  |  |  |  |  |  |  |  |  |  |  |
| **Neutral Loci** |  |  |  |  |  |  |  |  |  |  |  |  |  |  |  |  |  |  |  |  |  |  |  |
| OMS00002 |  | - | - | - | - | - |  | - | - | - | - | - |  | - | - | - | - | - |  | - | ******* | - | ******* |
| OMS00013 |  | - | - | - | - | ******* |  | ******* | ******* | - | - | ******* |  | - | - | - | - | - |  | ******* | ******* | - | ******* |
| OMS00024 |  | - | - | - | - | - |  | - | - | - | - | - |  | ******* | - | - | - | - |  | - | - | - | ******* |
| OMS00053 |  | - | - | - | - | - |  | - | - | - | - | - |  | - | - | - | - | - |  | - | ******* | - | ******* |
| OMS00074 |  | - | ******* | - | - | ******* |  | - | ******* | - | - | ******* |  | - | - | - | - | - |  | - | ******* | - | ******* |
| OMS00089 |  | - | - | - | - | - |  | - | - | - | - | - |  | ******* | ******* | - | - | ******* |  | - | - | - | - |
| OMS00092 |  | - | - | - | - | - |  | - | - | - | - | - |  | ******* | ******* | - | - | ******* |  | - | - | - | - |
| OMS00111 |  | - | - | - | - | - |  | ******* | ******* | ******* | ******* | ******* |  | - | - | - | - | - |  | ******* | - | - | ******* |
| OMS00179 |  | - | - | - | - | - |  | - | - | - | - | - |  | - | - | - | - | ******* |  | - | ******* | - | - |
| OMS00180 |  | - | - | - | - | - |  | - | ******* | - | - | - |  | - | - | - | - | - |  | - | ******* | - | - |
| Omy_105385-406 |  | - | - | - | - | - |  | - | - | - | - | - |  | - | - | - | - | - |  | - | ******* | - | ******* |
| Omy_107806-34 |  | - | - | - | - | - |  | - | - | - | - | - |  | ******* | - | - | - | - |  | - | - | - | ******* |
| Omy_128996-481 |  | - | ******* | - | - | ******* |  | - | ******* | - | - | ******* |  | - | - | ******* | ******* | - |  | ******* | ******* | - | ******* |
| Omy_BAC-B4-324 |  | - | - | - | - | - |  | - | - | - | - | - |  | - | - | - | - | - |  | - | ******* | - | ******* |
| Omy_bcAKala-380rd |  | - | - | - | - | - |  | - | - | - | - | - |  | - | - | - | - | - |  | - | ******* | - | ******* |
| Omy_carban1-264 |  | - | - | - | - | - |  | - | - | - | - | - |  | ******* | - | - | - | - |  | - | - | - | ******* |
| Omy_cd28-130 |  | - | - | - | - | - |  | - | - | - | - | - |  | - | - | ******* | ******* | - |  | ******* | ******* | - | ******* |
| Omy_gadd45-332 |  | - | - | - | - | - |  | ******* | ******* | - | - | ******* |  | - | - | - | - | - |  | ******* | ******* | - | - |
| Omy_hus1-52 |  | ******* | ******* | - | - | ******* |  | ******* | ******* | ******* | ******* | ******* |  | ******* | - | ******* | ******* | - |  | ******* | ******* | - | ******* |
| Omy_Il-1b_.028 |  | ******* | ******* | - | - | ******* |  | ******* | ******* | ******* | ******* | ******* |  | - | - | - | - | - |  | ******* | ******* | - | ******* |
| Omy_LDHB-2_e5 |  | - | - | - | - | - |  | - | - | - | - | - |  | - | - | ******* | - | - |  | - | ******* | - | ******* |
| Omy_myoD-178 |  | - | - | - | - | - |  | - | - | - | - | - |  | ******* | - | - | - | ******* |  | - | - | - | - |
| Omy_NaKATPa3-50 |  | - | - | - | - | - |  | - | - | - | - | - |  | ******* | ******* | - | - | - |  | - | - | - | - |
| Omy_Ots249-227 |  | - | - | - | - | - |  | - | ******* | - | - | ******* |  | - | - | - | - | - |  | - | ******* | - | - |
| Omy_p53-262 |  | - | - | - | - | - |  | ******* | ******* | - | ******* | ******* |  | - | - | - | ******* | - |  | ******* | ******* | - | ******* |
| Omy_sSOD-1 |  | - | - | - | - | - |  | - | - | - | - | - |  | - | - | ******* | ******* | - |  | - | ******* | - | ******* |
| Omy_u07-79-166 |  | - | - | - | - | - |  | - | - | - | - | - |  | - | - | - | ******* | - |  | ******* | ******* | - | - |
| Omy_vamp5-303 |  | - | - | - | - | - |  | - | - | - | - | - |  | - | - | - | - | - |  | - | ******* | - | ******* |
|  |  |  |  |  |  |  |  |  |  |  |  |  |  |  |  |  |  |  |  |  |  |  |  |
| **COASTAL** |  |  |  |  |  |  |  |  |  |  |  |  |  |  |  |  |  |  |  |  |  |  |  |
|  |  |  |  |  |  |  |  |  |  |  |  |  |  |  |  |  |  |  |  |  |  |  |  |
| **Candidate Loci** |  |  |  |  |  |  |  |  |  |  |  |  |  |  |  |  |  |  |  |  |  |  |  |
| Omy_hsf2-146 | (4) | - | - | - | - | - |  | - | - | - | - | - |  | - | - | - | - | - |  | - | - | - | - |
| Omy_stat3-273 | (2) | - | - | - | - | - |  | - | - | - | - | - |  | ******* | - | - | ******* | ******* |  | - | - | - | - |
| OMS00058 |  | - | - | - | - | - |  | - | - | - | - | - |  | - | - | - | - | - |  | - | ******* | ******* | - |
| OMS00111 |  | - | - | - | - | - |  | - | - | - | - | - |  | - | - | - | - | - |  | - | - | ******* | - |
| OMS00008 |  | - | - | - | - | - |  | - | - | - | - | - |  | - | - | ******* | - | - |  | - | - | - | - |
| Omy_aldB-165 | (2) | - | - | - | - | - |  | - | - | - | - | - |  | - | - | - | - | - |  | - | - | - | - |
| Omy_OmyP9-180 | (1) | - | - | - | - | - |  | - | - | - | - | - |  | - | - | - | - | - |  | - | - | - | - |
| Omy_bcAKala-380rd | X | - | - | - | - | - |  | - | - | - | - | - |  | - | - | - | - | - |  | - | - | - | - |
| Omy_cox1-221 |  | - | - | - | - | - |  | - | - | - | - | - |  | - | - | - | - | - |  | - | ******* | ******* | - |
|  |  |  |  |  |  |  |  |  |  |  |  |  |  |  |  |  |  |  |  |  |  |  |  |
| **"Ambiguous"** |  |  |  |  |  |  |  |  |  |  |  |  |  |  |  |  |  |  |  |  |  |  |  |
| Omy_tlr5-205 | (2) | - | - | - | - | - |  | - | - | - | - | - |  | - | - | - | - | - |  | - | - | - | - |
| Omy_hsp47-86 | (1) | - | - | - | - | - |  | - | - | - | - | - |  | - | - | - | - | - |  | - | - | - | - |
| Omy_ndk-152 | (3) | - | - | - | - | - |  | - | - | - | - | - |  | - | - | - | - | - |  | - | - | - | - |
| Omy_gdh-271 | (2) | - | - | - | - | - |  | - | - | - | - | - |  | - | - | - | - | - |  | - | - | - | - |
| OMS00064 | X | - | - | - | - | - |  | - | - | - | - | - |  | - | - | - | - | - |  | - | - | - | - |
| OMS00096 | X | - | - | - | - | - |  | - | - | - | - | - |  | - | - | - | - | - |  | - | - | - | - |
| OMS00118 | X | - | - | - | - | - |  | - | - | - | - | - |  | - | - | - | - | - |  | - | - | - | - |
| OMS00174 | X | - | - | - | - | - |  | - | - | - | - | - |  | - | - | - | - | - |  | - | - | - | - |
| Omy_97954-618 | X | - | - | - | - | - |  | - | - | - | - | - |  | - | - | - | - | - |  | - | - | - | - |
| Omy_cd59-206 | X | - | - | - | - | - |  | - | - | - | - | - |  | - | - | - | - | - |  | - | - | - | - |
| Omy_metB-138 | X | - | - | - | - | - |  | - | - | - | - | - |  | - | - | - | - | - |  | - | - | - | - |
| Omy_Ogo4-212 | (2) | - | - | - | - | - |  | - | - | - | - | - |  | - | - | - | - | - |  | - | - | - | - |
| Omy_LDHB-2_i6 | (3) | - | - | - | - | - |  | - | - | - | - | - |  | - | - | - | - | - |  | - | - | - | ******* |
|  |  |  |  |  |  |  |  |  |  |  |  |  |  |  |  |  |  |  |  |  |  |  |  |
| **Neutral Loci** |  |  |  |  |  |  |  |  |  |  |  |  |  |  |  |  |  |  |  |  |  |  |  |
| Omy_colla1-525 |  | - | - | - | - | - |  | - | - | - | - | - |  | - | - | - | - | - |  | - | - | - | ******* |
| Omy_b1-266 |  | - | - | - | - | - |  | - | - | - | - | - |  | - | - | - | - | - |  | - | - | ******* | - |
| OMS00057 |  | - | - | - | - | - |  | - | - | - | - | - |  | - | - | - | - | - |  | - | - | ******* | - |
| Omy_metA-161 |  | - | - | - | - | - |  | - | - | - | - | - |  | - | - | - | - | - |  | ******* | - | - | ******* |
|  |  |  |  |  |  |  |  |  |  |  |  |  |  |  |  |  |  |  |  |  |  |  |  |
|  |  |  |  |  |  |  |  |  |  |  |  |  |  |  |  |  |  |  |  |  |  |  |  |

Table S7. List of final locus classifications based on significance in association tests. The list includes only loci that met at least one of the four candidate criteria (see methods section), and neutral loci listed are only those that were initially flagged as possible candidates. Outlier loci are marker by “X”, and numbers in parentheses refer to landscape association precedence (referenced in Table 2). Candidate loci were used in analysis of non-neutral differentiation, neutral loci were added to the existing panel of neutral loci for analysis of neutral differentiation, and “ambiguous” loci were not included in either analysis. DISTLM results are listed for individual predictor variables and sets of variables. The “1^st^ rank” significant results identify the variable responsible for the greatest sum of squares proportion (prop. SS). Seasonal variable are: W – winter, SP – spring, SU – summer, and F – fall.

|  |  |  |  |  |  |  |  |  |
| --- | --- | --- | --- | --- | --- | --- | --- | --- |
| DISTLM *forward* |  |  |  |  |  |  |  |  |
|  |  | conditional (individual) | |  |  | conditional (sets) | |  |
| SNP |  | 1st rank predictor | P-value | prop. (SS) |  | 1st rank predictor | P-value | prop. (SS) |
|  |  |  |  |  |  |  |  |  |
| **INLAND** |  |  |  |  |  |  |  |  |
|  |  |  |  |  |  |  |  |  |
| **Candidate Loci** |  |  |  |  |  |  |  |  |
| Omy_97660-230 |  | Migration | 0.0002 | 0.3404 |  | Tmin | 0.0004 | 0.4056 |
| Omy_e1-147 |  | Longitude | 0.0002 | 0.5077 |  | Tmax | 0.0002 | 0.5878 |
| Omy_hsc715-80 |  | Precip. (SU) | 0.0002 | 0.5700 |  | Precipitation | 0.0002 | 0.5905 |
| Omy_hsp47-86 | (1) | Migration | 0.0002 | 0.3038 |  | Migration | 0.0002 | 0.3038 |
| Omy_ntl-27 |  | Longitude | 0.0002 | 0.4939 |  | Lat/Long | 0.0002 | 0.4926 |
| Omy_SECC22b-88 |  | Precip. (SP) | 0.0004 | 0.3152 |  | Precipitation | 0.0006 | 0.4043 |
| OMS00014 |  | Precip. (SP) | 0.0002 | 0.3693 |  | EV | 0.0002 | 0.5580 |
| OMS00062 |  | Precip. (F) | 0.0002 | 0.3186 |  | EV | 0.0002 | 0.3961 |
| Omy_97954-618 |  | Longitude | 0.0002 | 0.2982 |  | *Q* | 0.0002 | 0.5680 |
| Omy_CRBF1-1 |  | Longitude | 0.0002 | 0.6295 |  | *Q* | 0.0002 | 0.7883 |
| Omy_gdh-271 | (2) | Elevation | 0.0026 | 0.1479 |  | *Q* | 0.0048 | 0.2545 |
| Omy_GHSR-121 |  | Precip. (SP) | 0.0002 | 0.4948 |  | *Q* | 0.0002 | 0.7193 |
| Omy_IL6-320 |  | Tmin (W) | 0.0028 | 0.0911 |  | EV | 0.0002 | 0.3906 |
| Omy_nkef-241 |  | Longitude | 0.0002 | 0.4728 |  | *Q* | 0.0002 | 0.6887 |
| Omy_OmyP9-180 | (1) | Precip. (SP) | 0.0002 | 0.2778 |  | *Q* | 0.0002 | 0.4049 |
| Omy_tlr5-205 | (2) | Longitude | 0.0002 | 0.6354 |  | EV | 0.0002 | 0.6740 |
| Omy_u09-53.469 | X | Longitude | 0.0002 | 0.5548 |  | *Q* | 0.0002 | 0.7615 |
| Omy_UT16_2-173 | X | Migration | 0.0002 | 0.2955 |  | EV | 0.0002 | 0.5579 |
| OMY1011SNP |  | Precip. (SP) | 0.0002 | 0.5022 |  | EV | 0.0002 | 0.7633 |
| OMS00151 |  | *Q1* | 0.0002 | 0.1895 |  | Precipitation | 0.0006 | 0.4085 |
| Omy_metA-161 |  | EV1 | 0.0002 | 0.3707 |  | Tmax | 0.0002 | 0.4276 |
| Omy_stat3-273 | (2) | *Q3* | 0.0002 | 0.3501 |  | Precipitation | 0.0004 | 0.3551 |
|  |  |  |  |  |  |  |  |  |
| **"Ambiguous"** |  |  |  |  |  |  |  |  |
| Omy_anp-17 | X | EV1 | 0.0002 | 0.8250 |  | EV | 0.0002 | 0.9259 |
| Omy_b9-164 | X | *Q3* | 0.0002 | 0.8250 |  | *Q* | 0.0002 | 0.8887 |
| Omy_IL1b-163 | X | EV2 | 0.0002 | 0.6394 |  | *Q* | 0.0002 | 0.9250 |
| Omy_ndk-152 | X; (3) | *Q2* | 0.0002 | 0.9452 |  | *Q* | 0.0002 | 0.9385 |
| Omy_nxt2-273 | X | EV2 | 0.0002 | 0.6790 |  | EV | 0.0002 | 0.7452 |
| Omy_star-206 | X | *Q2* | 0.0002 | 0.5669 |  | *Q* | 0.0002 | 0.6971 |
| Omy_u09-56.119 | X | *Q2* | 0.0002 | 0.9402 |  | EV | 0.0002 | 0.9544 |
| Omy_vatf-406 | X | *Q1* | 0.0002 | 0.1386 |  | *Q* | 0.0002 | 0.5856 |
| Omy_LDHB-2_i6 | (3) | *Q2* | 0.0002 | 0.7776 |  | *Q* | 0.0002 | 0.7554 |
| Omy_hsf2-146 | (4) | EV1 | 0.0002 | 0.5501 |  | EV | 0.0002 | 0.8971 |
| Omy_aldB-165 | (2) | EV2 | 0.0002 | 0.2510 |  | EV | 0.0018 | 0.2530 |
| Omy_Ogo4-212 | (2) | EV3 | 0.0002 | 0.5589 |  | EV | 0.0002 | 0.7163 |
|  |  |  |  |  |  |  |  |  |
| **Neutral Loci** |  |  |  |  |  |  |  |  |
| OMS00002 |  | *Q6* | 0.0002 | 0.5298 |  | EV | 0.0002 | 0.6662 |
| OMS00013 |  | *Q2* | 0.0002 | 0.6708 |  | *Q* | 0.0002 | 0.7097 |
| OMS00024 |  | EV1 | 0.0002 | 0.5341 |  | EV | 0.0002 | 0.5983 |
| OMS00053 |  | EV2 | 0.0002 | 0.4462 |  | *Q* | 0.0002 | 0.5900 |
| OMS00074 |  | *Q1* | 0.0002 | 0.5542 |  | EV | 0.0002 | 0.9071 |
| OMS00089 |  | *Q6* | 0.0002 | 0.5585 |  | EV | 0.0002 | 0.7738 |
| OMS00092 |  | *Q3* | 0.0002 | 0.9013 |  | *Q* | 0.0002 | 0.8892 |
| OMS00111 |  | *Q6* | 0.0002 | 0.2997 |  | EV | 0.0002 | 0.4036 |
| OMS00179 |  | *Q5* | 0.0002 | 0.3101 |  | *Q* | 0.0002 | 0.4637 |
| OMS00180 |  | *Q2* | 0.0002 | 0.7477 |  | *Q* | 0.0002 | 0.6989 |
| Omy_105385-406 |  | *Q2* | 0.0002 | 0.5256 |  | EV | 0.0002 | 0.8126 |
| Omy_107806-34 |  | EV1 | 0.0002 | 0.7555 |  | EV | 0.0002 | 0.8785 |
| Omy_128996-481 |  | EV2 | 0.0002 | 0.7227 |  | EV | 0.0002 | 0.7593 |
| Omy_BAC-B4-324 |  | *Q2* | 0.0002 | 0.7237 |  | EV | 0.0002 | 0.7821 |
| Omy_bcAKala-380rd |  | *Q2* | 0.0002 | 0.4220 |  | *Q* | 0.0002 | 0.5841 |
| Omy_carban1-264 |  | EV1 | 0.0002 | 0.7805 |  | EV | 0.0002 | 0.8458 |
| Omy_cd28-130 |  | *Q2* | 0.0002 | 0.8530 |  | *Q* | 0.0002 | 0.8899 |
| Omy_gadd45-332 |  | *Q1* | 0.0002 | 0.8500 |  | EV | 0.0002 | 0.8414 |
| Omy_hus1-52 |  | *Q2* | 0.0024 | 0.0256 |  | EV | 0.0002 | 0.9793 |
| Omy_Il-1b_.028 |  | EV2 | 0.0002 | 0.5152 |  | *Q* | 0.0002 | 0.7801 |
| Omy_LDHB-2_e5 |  | *Q2* | 0.0002 | 0.4617 |  | *Q* | 0.0002 | 0.5002 |
| Omy_myoD-178 |  | EV2 | 0.0016 | 0.0946 |  | EV | 0.0002 | 0.9582 |
| Omy_NaKATPa3-50 |  | EV1 | 0.0002 | 0.4419 |  | *Q* | 0.0002 | 0.6739 |
| Omy_Ots249-227 |  | EV2 | 0.0002 | 0.3611 |  | *Q* | 0.0002 | 0.4354 |
| Omy_p53-262 |  | *Q1* | 0.0002 | 0.5077 |  | *Q* | 0.0002 | 0.6373 |
| Omy_sSOD-1 |  | *Q2* | 0.0002 | 0.9285 |  | *Q* | 0.0002 | 0.9289 |
| Omy_u07-79-166 |  | EV2 | 0.0002 | 0.9007 |  | *Q* | 0.0002 | 0.9127 |
| Omy_vamp5-303 |  | EV2 | 0.0002 | 0.0967 |  | EV | 0.0002 | 0.9094 |
|  |  |  |  |  |  |  |  |  |
| **COASTAL** |  |  |  |  |  |  |  |  |
|  |  |  |  |  |  |  |  |  |
| **Candidate Loci** |  |  |  |  |  |  |  |  |
| Omy_hsf2-146 | (4) | Migration | 0.0038 | 0.6793 |  | Precipitation | 0.0142 | 0.9109 |
| Omy_stat3-273 | (2) | Precip. (SU) | 0.0004 | 1.1776 |  | Precipitation | 0.0102 | 1.0553 |
| OMS00058 |  | Latitude | 0.0002 | 0.9858 |  | Tmax | 0.0006 | 1.2857 |
| OMS00111 |  | Latitude | 0.0004 | 0.8839 |  | Precipitation | 0.0090 | 0.8719 |
| OMS00008 |  | Precip. (SU) | 0.0004 | 0.9384 |  | *Q* | 0.0070 | 0.8915 |
| Omy_aldB-165 | (2) | Tmax (SP) | 0.0056 | 0.4985 |  | *Q* | 0.0498 | 0.3803 |
| Omy_OmyP9-180 | (1) | *Q3* | 0.0266 | 0.3042 |  | Tmin | 0.0176 | 0.2382 |
| Omy_bcAKala-380rd | X | Longitude | 0.0004 | 0.7550 |  | Tmin | 0.0010 | 0.9084 |
| Omy_cox1-221 |  | Latitude | 0.0002 | 0.8240 |  | Lat/Long | 0.0056 | 0.7102 |
|  |  |  |  |  |  |  |  |  |
| **"Ambiguous"** |  |  |  |  |  |  |  |  |
| Omy_hsp47-86 | (1) | Precip. (SP) | 0.1188 | 0.1889 |  | Precipitation | 0.0768 | 0.3663 |
| Omy_tlr5-205 | (2) | EV3 | 0.1460 | 0.1350 |  | Elevation | 0.4088 | 0.0253 |
| Omy_ndk-152 | (3) | *Q6* | 0.0014 | 0.7572 |  | *Q* | 0.0082 | 0.8757 |
| Omy_gdh-271 | (2) | EV2 | 0.0136 | 0.4600 |  | EV | 0.0404 | 0.5390 |
| OMS00064 | X | EV1 | 0.0002 | 1.1278 |  | *Q* | 0.0002 | 1.1172 |
| OMS00096 | X | *Q2* | 0.0020 | 0.5578 |  | *Q* | 0.0002 | 1.1004 |
| OMS00118 | X | Longitude | 0.0006 | 0.7063 |  | EV | 0.0002 | 1.2182 |
| OMS00174 | X | EV1 | 0.0002 | 0.9197 |  | EV | 0.0002 | 0.9173 |
| Omy_97954-618 | X | *Q1* | 0.0054 | 0.8800 |  | *Q* | 0.0006 | 1.0254 |
| Omy_cd59-206 | X | EV1 | 0.0002 | 0.8827 |  | *Q* | 0.0002 | 1.0404 |
| Omy_metB-138 | X | *Q6* | 0.0008 | 0.9757 |  | *Q* | 0.0004 | 1.0661 |
| Omy_Ogo4-212 | (2) | EV1 | 0.0002 | 0.7727 |  | EV | 0.0002 | 1.0510 |
| Omy_LDHB-2_i6 | (3) | Longitude | 0.0002 | 1.1512 |  | Lat/Long | 0.0002 | 1.1473 |
|  |  |  |  |  |  |  |  |  |
| **Neutral Loci** |  |  |  |  |  |  |  |  |
| Omy_colla1-525 |  | Longitude | 0.0008 | 0.7152 |  | Lat/Long | 0.0004 | 0.9255 |
| Omy_b1-266 |  | Latitude | 0.0010 | 0.7356 |  | *Q* | 0.0008 | 1.0831 |
| OMS00057 |  | Latitude | 0.0002 | 0.7483 |  | *Q* | 0.0002 | 1.0264 |
| Omy_metA-161 |  | *Q2* | 0.0012 | 0.7623 |  | *Q* | 0.0122 | 0.8538 |
|  |  |  |  |  |  |  |  |  |
